# Polymerization of Myrcene in Both Conventional and Renewable Solvents: Postpolymerization Modification via Regioselective Photoinduced Thiol–Ene Chemistry for Use as Carbon Renewable Dispersants

**DOI:** 10.1021/acssuschemeng.2c03755

**Published:** 2022-07-11

**Authors:** Jirui Zhang, Cansu Aydogan, Georgios Patias, Timothy Smith, Lucas Al-Shok, Huizhe Liu, Ahmed M. Eissa, David M. Haddleton

**Affiliations:** † Department of Chemistry, University of Warwick, Gibbet Hill, Coventry CV4 7AL, United Kingdom; ‡ Lubrizol, Ltd., Nether Lane, Hazelwood, Derbyshire DE56 4AN, United Kingdom

**Keywords:** Biorenewable monomer, Anionic polymersation, Photoinduced thiol−ene, Regioselective functionalization, Thermal stability

## Abstract

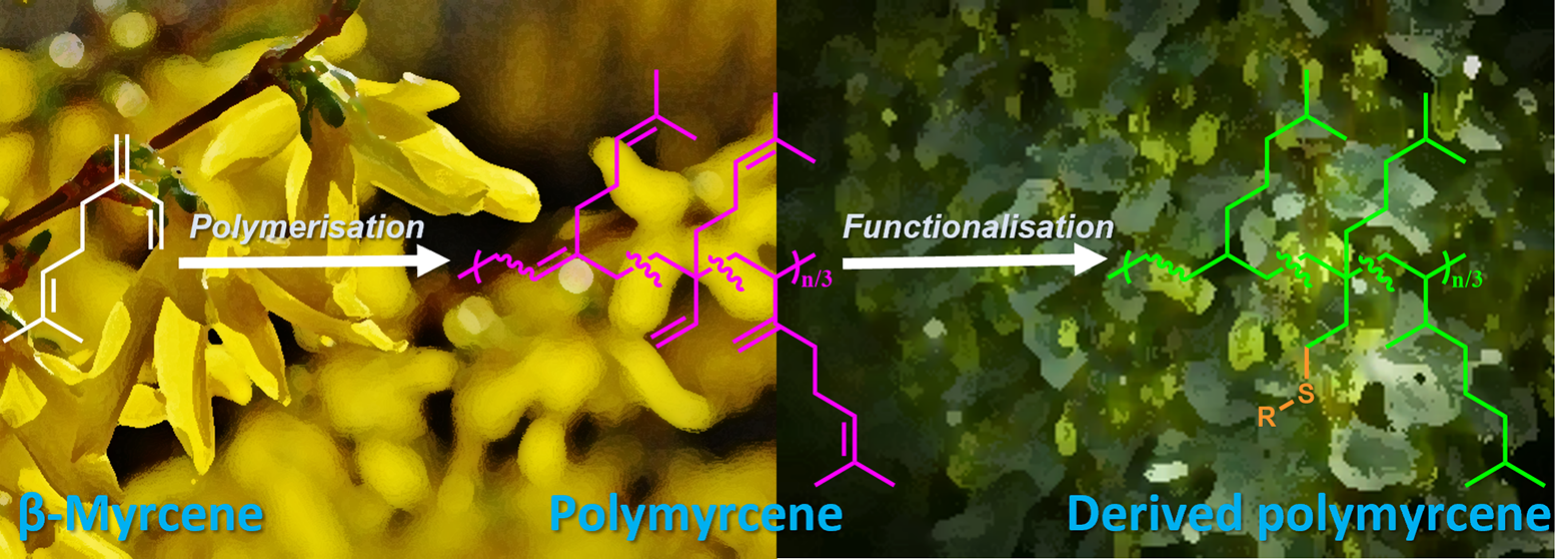

Polymeric dispersants are useful materials used in many different industries and often derived from oil-based chemicals, for example, in automotive fluids so as to prevent particulates from precipitation and causing potential damage. These are very often polyisobutene derivatives, and there is a growing need to replace these using chemicals using renewable resources such as the use of naturally occurring myrcene. Polymyrcene (PMy), with an ordered microstructure, has been successfully synthesized via both anionic and radical polymerization in different solvents and subsequently subjected to functionalization via photoinduced thiol–ene click reactions with a number of thiols, methyl thioglycolate, 3-mercaptopropionic acid, 3-mercapto-1-hexanol, 2-mercaptoethanol, and 1-thioglycerol, using 2,2-dimethoxy-2-phenylacetophenone as a photoinitiator under UV irradiation (λ = 365 nm) at ambient temperature. The polarity of the solvent has an important impact on the microstructure of the produced polymyrcene and, in particular, 1,2-unit (∼4%), 3,4-unit (∼41%), and 1,4-unit (∼51%) PMy were obtained via anionic polymerization in a polar solvent (THF) at ambient temperature, while 3,4-unit (∼6%) and 1,4-unit (∼94%, including *cis* and *trans*) PMy were obtained with cyclohexane as the solvent. Subsequently, photochemical thiol–ene reactions were carried out on the resulting PMy with different isomers exhibiting different reactivities of the double bonds. This strategy allows for the introduction of functional/polar groups (−COOH, −OH) into hydrophobic PMy in a controlled process. Hydrogenation of PMy and derivatized PMy was carried out to investigate any effects on the stabilities of the products which are desirable for many applications.

## Abstract 2

Polymerization and functionalization of renewable myrcene for use as an industrial dispersant as a replacement for functionalized oil-derived polyisobutene are discussed.

## Introduction

Sustainable polymers from renewable natural resources are of increasing importance so as to achieve a sustainable planet while continuing to enjoy the excellent material properties synthetic polymers can provide. This is driven by increasingly strict requirements from both regulators and demands by consumers.^1^ In order to continue to meet the ever-increasing demands on material properties at acceptable prices and to overcome the impending scarcity of petrochemicala, materials must focus on being sourced from bioderived and sustainable resources, thus reducing the negative effects on the planet throughout the life cycles of the materials.^2−4^ Indeed, polymers from natural resources have been used for many centuries and actually long before synthetic polymers entered our world, even before the concept of a polymer was introduced just over 100 years ago by Staudinger and Ochiai.^5^ Terpenes are a family of natural hydrocarbons available from many plants found in many of our daily products as “essential oils”.^6,7^ Terpenes are structurally composed of often different arrangements of isoprene (C5) units and include β-myrcene, alloocimene, limonene, farnesene, and α-/β-pinene.^8^ Natural polyisoprene elastomers, harvested from rubber trees, have been used for centuries and are still important in many applications including latex gloves, vehicle tires, children’s toys, and more. It was only the reduction of supply to certain markets in the two world wars of the last century that led to the rapid development of emulsion polymerization for synthetic rubber that is still widely used today.^9,10^ The alkenyl monoterpene, β-myrcene (7-methyl-3-methylene-octa-1,6-diene), is a natural dimer of isoprene and is a versatile monomer with similar reactivity to other petro-based unsaturated hydrocarbons which have already been utilized in commercial synthetic rubbers. Polymyrcene (PMy) has received historical attention as early as 1953 by the ESSO Corporation alongside their development of polyisoprene for use in car tires.^11^ However, the availability of inexpensive petrochemicals and the lack of realization of climatic issues at the time led these to be largely ignored and left underdeveloped. For example, terminally functionalized PMy was described by Stanford et al. as formed by both anionic and free radical polymerization primarily for the production of polyols for subsequent polyurethane synthesis.^12^ Due to a recent growing demand for bioderived polymers, myrcene (My) and polymyrcene (PMy) offer significant possibilities as potential components in sustainable materials for many applications.

Anionic polymerization is a robust method for the polymerization of various monomers including dienes (e.g., isoprene and butadiene), and it is the most widely used and commercialized living polymerization method used for over the last 50 years. It is successfully used for the production of high-performance elastomers in AB and ABA block copolymers of dienes and styrene, terminally functional polymers, and associated hydrogenated forms. Indeed, the commercial success of living anionic polymerization far dwarfs that of living radical polymerization in all of its forms combined. The logistical difficulties concerning the purity of reagents/solvents and the absence of water/protic impurities often seen in an academic laboratory are not always transposed to the commercial world when carrying out polymerizations at large scales. Importantly, the absence of termination and transfer steps allows controlled living polymerization and, ultimately, the formation of well-defined polymers (functional end groups or telechelic block copolymers) with narrow molar mass distributions and quantitative monomer conversions to polymer.

**Scheme 1:**
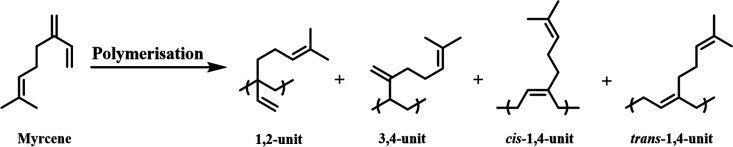
Schematic Representation for Different Potential Isomers/Microstructures of PMy

β-Myrcene (Scheme 1) has been polymerized by radical,^13−16^ anionic,^17−24^ cationic,^25−27^ and rare earth coordinations^28−41^ and emulsion polymerization^42−44^ to give polymers with a range of stereochemistries and microstructures containing four very different repeat units, i.e., *cis-* and *trans-*1,4-, 1,2-, and 3,4-units. The stereochemistry varies depending on the polymerization conditions, as for all diene polymerization, which in turn determines both the physical and chemical properties of the resulting polymers.^45−49^ For example, PMy with 77%–85% 1,4-units and relatively low dispersity (*Đ* ∼ 1.35) was obtained by free radical polymerization in *n*-butanol at ca. 100 °C and also found to exhibit branching and cross-linked side products due to the reactivity of the residual double bonds and the reactive radicals.^50−52^ Reversible addition–fragmentation chain-transfer (RAFT) polymerization of myrcene led to PMy with up to 65% monomer conversions and with *Đ* > 1.3. Behr and Johnen cited ∼96% 1,4- and ∼3% 3,4-units with <1% 1,2 stereochemistry,^8^ while a second report showed a temperature-dependent decrease, with the 1,4-polymerization reaching 96%, 90%, and 75% at temperatures of 65, 90, and 130 °C, respectively, along with significant branching at 130 °C and >50% high conversion by controlled radical polymerization.^13,15^ Rare earth coordination polymerization of myrcene, using different rare earth and transition metal catalysts (e.g., Nd, La, and Fe)^32,36^ with a range of different ligands allowed control over the microstructure in PMy with very high stereoregularity.^34^ Gan et. al. reported that the use of a Nd catalyst gives >93% *cis-*1,4 PMy,^41^ while Loughmari et al. showed >88% and up to >98% *cis-*1,4 PMy using Nd catalysts in conjunction with dialkyl magnesium compounds with a switch to 3,4-addition on increasing amounts of the butylethyl magnesium cocatalyst.^32^ Cui et al. reported that the use of a cationic lutetium catalyst led to up to 100% 3,4-addition at temperatures of >25 °C.^31^ Cationic polymerization leads to PMy with ca. 43% *cis-* and 50% *trans-*1,4 addition but often with limited monomer conversions (<80%) and higher dispersity (*Đ* > 2.3).^25^ The formation of 1,2-vinylic units is least favored, and in the polymerization of isoprene, it can be promoted by using anionic polymerization in polar aprotic solvents such as diethyl ether with up to 22% of the formed stereoisomers with a sodium counterion.^54^ Gallei et al. report the formation of PMy-polystyrene block copolymers in THF from −78 °C to room temperature with approximately 25% 1,2-PMy,^53^ and Schlaad et al. reported up to 9% 1,2-PMY in THF and 6% in 2-MeTHF at room temperature.^55^ Polymyrcene with almost exclusively 1,4-units (and low levels of 3,4-units) is attained via anionic polymerization in nonpolar solvents (e.g., cyclohexane), and it was also noticed that as the concentration of the polymer increases, and therefore the hydrophobicity of the solution, the 1,4-addition is favored.^17,18,21^

The very different reactivity of each type of alkenyl group in PMy offers the possibility for selective postpolymerization functionalization, and hence, PMy can be utilized for postmodification, e.g., thiol–ene addition, epoxidation, hydrosilylation, hydrogenation, and even controlled cross-linking. Thiol–ene chemistry^56^ is a facile approach for postmodification of alkenyl functional polymers due to its high efficiency, ambient oxygen or water tolerance, and a wide range of potential applications from biological to surface functional materials.^57−59^ Thiol–ene chemistry has been demonstrated by Meier et al. for the functionalization of polymers derived from limonene and pinene.^60^ In 2015, Cui and co-workers reported the functionalization of highly *cis-*1,4-selective and 1,2-regioselective poly(3-methlenehepta-1,6-diene) with a library of thiols by rapid photoinduced thiol–ene chemistry in order to increase the functionality in thermal and surface properties.^61,62^ Amphiphilic PMy derivatives were obtained by both thiol–ene reaction and epoxidation to yield 3D-printed scaffolds^21^ and epoxy resins,^17^ respectively. Generally, thiol–ene additions are performed under mild conditions and initiated thermally or photochemically with either radical initiators or directly under UV irradiation. In many of these recent reports, PMy has been prepared by anionic polymerization in nonpolar solvents such as cyclohexane^17,18^ or anionic in the absence of solvent (very nonpolar) and free radical polymerization,^21^ none of which lead to significant 1,2 polymer content. Polymers with monosubstituted alkenyl, vinyl double bonds are prone to reacting with the formed thyil radicals which can result in high yields with an excellent regioselective *anti*-Markovnikov addition (Scheme S1, Supporting Information). Conversely, polymers with a high level of substituted alkenes may cause an increase in the reversibility of the thiyl radical addition step.^63,64^

**Scheme 2:**
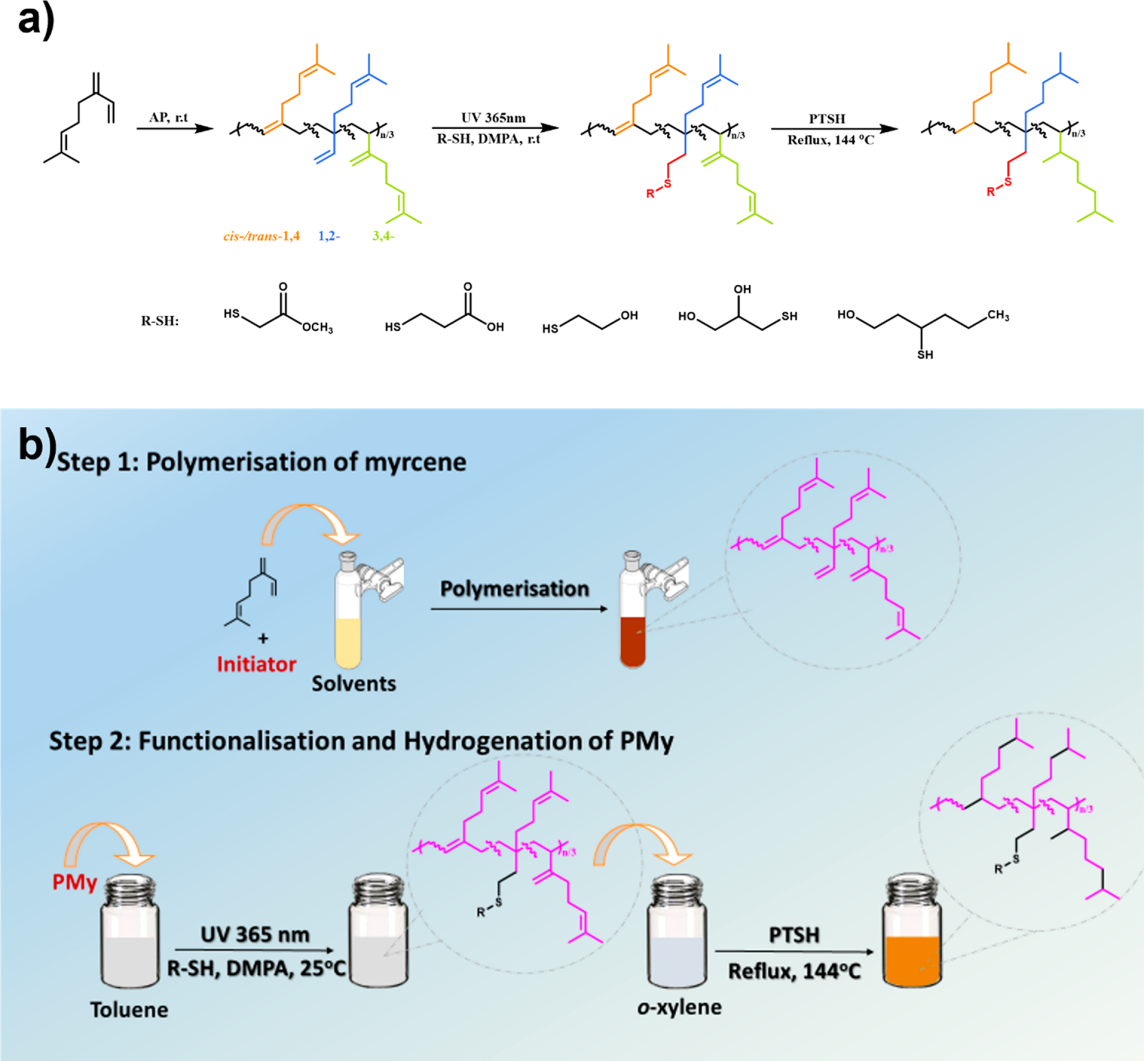
Schematic Representation for Synthesis of PMy and Thiol-Derivatized PMy in This Work

Herein, PMy has been synthesized via both free radical and anionic polymerizations in a range of different solvents and subsequently functionalized by photoinduced thiol–ene click chemistry with a number of hydrophilic functional thiols (methyl thioglycolate, 2-mercaptoethanol, 3-mercaptopropionic acid, 3-mercapto-1-hexanol, and 1-thiyoglycerol) for the purpose of introducing polar groups into hydrophobic PMy in the presence of 2,2-dimethoxy-2-phenylacetophenone (DMPA) as a photoinitiator with UV irradiation (Scheme 2). By utilizing thiol–ene photochemistry, the relative reactivities of the different alkenyl groups of polymyrcene have been investigated. Subsequently, hydrogenation, which is often desirable to increase both thermal and chemically oxidative stabilities and cross-linking, was investigated. The goal of this work was to investigate the potential for biorenewable replacements to replace functionalized polyisobutene, which is commonly and widely used in automotive and industrial dispersants to prevent precipitation of particulates. In addition to using renewable monomers and solvents, we were also interested in exploring lowering the energy requirements of the process by limiting the need for distillation and excessive reagent/solvent purification and for energy for heating or cooling.

## Materials and Methods

### Materials

β-Myrcene (My, technical grade) was purchased from Sigma-Aldrich and transferred and stored in a Schlenk tube over a dried molecular sieve (3 Å, general purpose grade) under a nitrogen atmosphere. Anhydrous tetrahydrofuran, cyclohexane, dioxane, diethyl ether, 2-methyltetrahydrofuran (2-MeTHF), and *n*-butyl lithium were purchased from Sigma-Aldrich. Squalane was dried over molecular sieves (3 Å, general purpose grade) under a nitrogen atmosphere. Methyl thioglycolate (95%), 3-mercaptopropionic acid (≥99%), 3-mercapto-1-hexanol (99%), 1-thiyoglycerol (95%), 2-mercaptoethanol (≥99.0%), and 2,2-dimethoxy-2-phenylacetophenone were used as received and purchased from Sigma-Aldrich.

### Characterization

#### Nuclear Magnetic Resonance

^1^H and ^13^C NMR spectra were obtained from Bruker DPX-400 and DPX-500 spectrometers using perdeuterated solvents purchased from Sigma-Aldrich. Monomer conversion was calculated via integration of the ^1^H NMR using a comparison of vinyl protons from the residual monomer with protons arising from the polymer backbone. An attached proton test ^13^C NMR (APT-^13^C NMR) experiment was used to for the pure polymyrcene to analyze the stereochemistries of the products.

#### Size Exclusion Chromatography

SEC was used for measurement of the molar mass and dispersity of the polymers and collected on an Agilent Infinity II MDS instrument using CHCl_3_ containing 2% TEA (triethylamine) as the mobile phase at 30 °C. The equipment was fitted with a differential refractive index (DRI), dual angle UV and viscometry detectors, 2 PLgel mixed C columns (300 mm × 7.5 mm), and a PLgel 5 μm guard column for separation and an autosampler for sample injection. Narrow molar mass poly(methyl methacrylate) and polystyrene standards (Agilent EasyVials) were used for calibration of the DRI trace. Ethanol was added to the eluent and used as a flow rate marker. Analyte samples were filtered through a GVHP membrane with 0.22 μm pore size before injection, and all samples were passed through 0.2 μm PTFE filter before analysis.

#### Matrix-Assisted Laser Desorption Ionization Time-of-Flight

MALDI-ToF mass spectra were collected in reflectron positive mode with a 21 kV acceleration voltage and a 25 kV reflection voltage. The laser power was kept as low as possible with 10,000 shots being accumulated to create the spectra. Samples of the homopolymyrcene were prepared in CHCl_3_ at a concentration of 10 mg/mL, with an addition of 1 mg/mL of AgTFA as a cationizing agent. The samples were then mixed 1:1 with a 40 mg/mL solution of *trans-*2-[3-(4-*tert-*butylphenyl)-2-methyl-2 propenylidene] malononitrile (DCTB) in CHCl_3_. Here, 0.5 mL of each sample was taken and then spotted on an MTP 384 ground steel target plate and analyzed using a Bruker AutoFlex Speed ToF/ToF analyzer equipped with a 337 nm nitrogen laser.

#### Thermogravimetric Analysis

TGA measurements were carried out on a TA Instruments TGA with an autosampler. N_2_ gas was used with a heating rate of 10 °C/min in alumina pans from 25 to 600 °C.

#### Differential Scanning Calorimetry

DSC measurements were carried out on a TA Instruments DSC with an autosampler. The samples were placed in alumina crucibles (with pierced lids) and heated/cooled from −100 to 250 °C in a flow of N_2_ with a heating rate of 10 °C/min. The results of the second heating cycle are reported in all cases.

#### Oxygen Probe

OP measurements were carried out by a pocket oxygen meter FireStingGO2 (from Pyro Science) to determine the oxygen consumption during polymerization. The solvent-resistant oxygen probe OXSOLV measures oxygen partial pressure in most polar and nonpolar solvents. It is based on optical detection principles (REDFLASH technology) and can be used both in THF. The fiber optic oxygen sensor tip is covered with a stainless-steel tube 1.5 mm in diameter and 150 mm in length. The analysis of the data was conducted with the FireStingGO2Manager software.

#### Gas Chromatography-Flame Ionization Detection Spectrometry

GC-FID measurements were carried out by Shimadzu GC2014 with a Restek Rxi-1 ms column (14.9 m, 0.25 mm I.D., 0.25 μm) and helium as the carrier gas to determine the impurities of green solvents. Injections were 1 μL via robot. The injection temperature was 300 °C with 25 split ratios. The method followed was an initial temperature of 100 °C for 1 min equilibration, then heated to 160 °C held for 2.5 min followed by heating to 320 °C at 25 °C min^–1^ and held for 10.6 min, for a total run time of approximately 24.50 min.

### Synthetic Procedure

#### Procedure for Homopolymerization of Myrcene via Emulsion Polymerization (R3, Minor E)

In an emulsion polymerization of myrcene with ammonium persulfate (APS, 0.0875 g, 0.035%) as the initiator, the emulsifier (sodium dodecyl sulfate, SDS) (0.625 g, 2.5%), buffer (NaHCO_3_, 0.375 g, 1.5%), chain transfer agent (1-dodecanthiol, 2.5 g, 10%), and DI water (62.5 g) were charged into a three-necked round-bottomed flask equipped with a magnetic stirring bar. The mixture was purged with nitrogen and stirred at 400 rpm for 1 h. Subsequently, the mixture was heated to 70 °C. Addition of 25 g of predeoxygenated myrcene monomer was with a deoxygenated syringe and a syringe pump when the addition was finished. The mixture was left overnight.

#### General Procedure for Homopolymerization of Myrcene via Anionic Polymerization (without/with) O_2_ (DP_n_ = 10)

Anhydrous THF (10 mL) and myrcene (My) (2.9 mmol, 5 mL) were charged into a vacuum flame-dried Schlenk tube with a magnetic stirring bar fitted with a rubber septum (under 10% of injected air though glass syringe). *n*-Butyl lithium solution in hexane (1.6 M, 0.29 mmol, 1.8 mL) was subsequently added to the reaction mixture. The reaction was stirred, allowed to exotherm, and subsequently allowed to cool following the initial polymerization exotherm. Following the reaction, the mixture was quenched with methanol, the polymer isolated by precipitation into a large excess of methanol, and the solvent removed under vacuum prior to characterization.

#### General Procedure for Thiol–Ene Photofunctionalization

A solution of PMy (100 mg), methyl thioglycolate (0.075 equiv for per myrcene unit), and photoinitiator (DMPA) (0.05 equiv for per myrcene unit) in 2 mL of toluene was introduced in a Pyrex tube and irradiated at λ = 365 nm under a nitrogen atmosphere with no temperature control. After 1 h, the product was precipitated into an excess of methanol and volatiles removed in a vacuum oven until reaching a constant weight. All of the other thiol–ene photofunctionalization reactions were performed under similar conditions.

#### Hydrogenation^65^

Partially functionalized PMy (100 mg) and 680 mg of *p*-toluenesulphonyl hydrazide (PTSH) (5 equiv for each myrcene unit) were dissolved in 5 mL of *o*-xylene, and the mixture was magnetically stirred under reflux at 144 °C for 6 h. The resulting product was precipitated three times into methanol and volatiles removed in a vacuum oven until reaching a constant weight.

## Results and Discussion

### Polymyrcene Synthesis

A range of different PMy stereoisomers were prepared by anionic and radical polymerizations (Schemes S2–S5). Different stereoisomers (1,4-, 3,4-, and 1,2-units) were obtained via the different polymerization methods.^66^ High 1,4-unit content, ∼94%, and ∼6% 3,4-units were observed with free radical bulk polymerization, aqueous emulsion polymerization, and anionic polymerization in cyclohexane (Figure S1) with similar results to those previously reported,^17,18,21^ while anionic polymerization gave higher monomer conversions over shorter time periods (Table 1). The SEC traces of the PMy products are shown in Figure S2. PMy as synthesized by free radical polymerization has a broader molar mass distribution (*Đ* ∼ 1.99) with limited monomer conversion (∼72%) after 3 days. Emulsion polymerization of myrcene led to undesirable cross-linking and subsequent gelation resulting in very high observed dispersity of the limited amount of soluble material (*Đ* ∼ 8.06) at higher monomer conversions after 24 h, which was undesirable in this current work. Unless there are complications arising from a particular solvent or impurities present, anionic polymerization leads to full monomer conversion (>99%), as determined by ^1^H NMR, and control over the molar mass of polymers with quite narrow molar mass distributions is attained (Figure S2). It is noted that the synthesis reported here was carried out with commercial anhydrous solvents usiing standard Schlenk line techniques and monomers dried by standing over anhydrous molecular sieves overnight for practical application and to reduce energy requirements as opposed to more stringent conditions which would be expected to lead to narrower molar mass distribution.

**Table 1:** Summary of Homopolymerization of Myrcene

						Microstructure^h^ (%)			
						1,4-unit		3,4-unit	1,2-unit
Entry	Sample	Solvent	*M*_n,SEC_^g^ (g/mol)	Conv^h^(%)	*Đ*^g^	*cis*	*trans*		
1^a^	PMy_FRP_	Bulk	10,900	∼72	1.99	50	46	4	–
2	PMy_EP_	H_2_O	3900	>99	8.06	40	56	4	–
3^f^	PMy_10_	THF	2000	>99	1.21	2	54	42	2
4^f^	PMy_25_	THF	4600	>99	1.19	1	53	43	3
5^b^	PMy _25_	THF	4100	>99	1.21	2	52	44	2
6^c^	PMy_25_	THF	3500	>99	1.21	2	51	45	2
7^d^	PMy_25_	THF	3900	>99	1.24	4	52	41	3
8^e^	PMy_25_	THF	3900	>99	1.19	3	51	43	3
9^f^	PMy_50_	THF	7000	>99	1.13	2	50	45	3
10^f^	PMy_100_	THF	13,500	>99	1.24	2	52	42	4
11^f^	PMy_200_	THF	22,800	>99	1.28	2	49	46	3
12^f^	PMy_100_	Cyclohexane	25,800	>99	1.36	84	10	6	–
13^f^	PMy_100_	Diethyl ether	14,400	>99	1.22	28.1	37	34.5	0.4
14^f^	PMy_100_	Dioxane	28,100	∼21	1.72	2	47	48	3
15^f^	PMy_100_	2-MeTHF	16,000	∼97	2.00	3	51	42	2
16^f^	PMy_100_	Squalane	16,000	>99	1.84	83	11	6	–

a Homopolymerization of myrcene by free radical polymerization, at 65 °C, with 2.25 wt % V-601 initiator for 3 days.

b *V*_My_:*V*_THF_ = 2:1.

c *V*_My_:*V*_THF_ = 1:1.

d *V*_My_:*V*_THF_ = 1:3.

e *V*_My_:*V*_THF_ = 1:4.

f *V*_My_:*V*_THF_ = 1:2.

g Determined by CHCl_3_–SEC analysis and expressed as molar mass equivalents to PMMA narrow molar mass standards.

h Conversion was calculated via ^1^H NMR and ^13^C NMR using CHCl_3_-*d* as the solvent (for NMR spectra and calculations, see SI). Reaction time: entries 3–9, left to commence for 30 min; entries 10–15, left to commence for 3 h, and entries 2 and 16, left to commence overnight.

**Figure 1:**
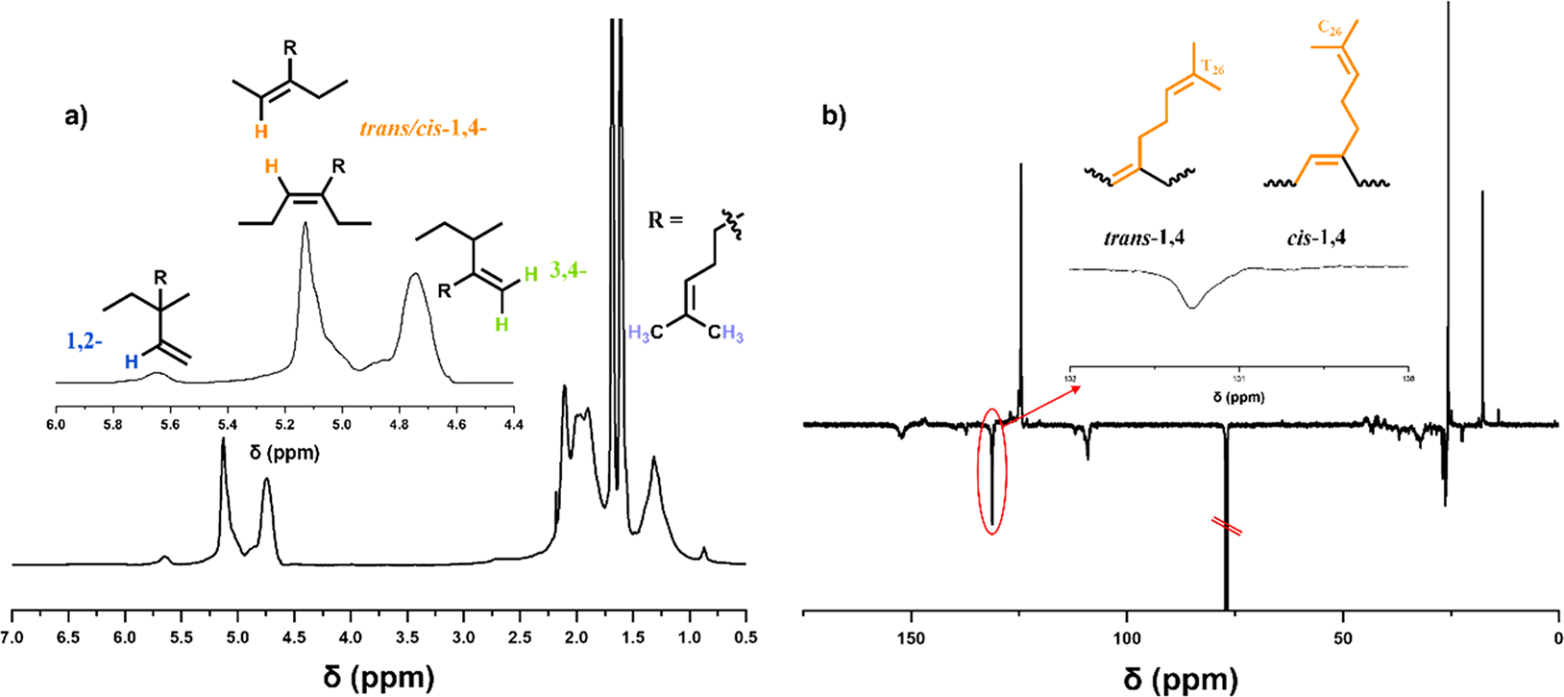
(a) ^1^H and (b) ^13^C NMR spectrum (500 MHz, CDCl_3_-*d*) of PMy (targeted DP_n_ = 100; Table 1, entry 10), synthesized via anionic polymerization in THF, 100 mg mL^–1^ of solution in CDCl_3_-*d*, using ^13^C NMR (APT-^13^C NMR) for the analysis of stereochemistry (R2, Q3).

First, anionic polymerization of My with targeted DP_n_ = 100 in THF gave high monomer conversion (>99%) with ∼4% 1,2-units (vinylic), ∼42% 3,4-units, and ∼54% 1,4-units (including 2% of *cis* and 52% of *trans*) from ^1^H NMR (Figure 1(a); Table 1, entry 10). The protons from the methylene groups (H5, H6, H10, H21, H23, H24, and H30) appear as one broad peak at 1.8–2.4 ppm. The methylene protons from the 1,2-vinyl group appear upfield as a broad peak (δ = 0.8–1.5 ppm, H14 and H15), although the protons of the two methyl groups attached to C3, C17, and C26 in all of the different microstructures appear upfield (δ = 1.5 ppm and δ = 1.7 ppm, H1, H2, H18, H19, H27, and H28) (Figure S3). The highest amount of 1,2-PMy content was attained in THF with a very small amount in diethyl ether with no detectable 1,2-PMy observed in either cyclohexane or squalane (Figure S4).

An “attached proton test ^13^C NMR (APT-^13^C NMR)” was utilized for the analysis of stereochemistry for PMy. In this experiment, modulated peak intensities are produced for methine and methyl carbons appearing with a normal phase and those for methine and methylene type carbons appearing with an inverted phase (R2 Q3). Further ^13^C NMR of the peak at ∼152 ppm (Figure 1(b); Figure S5) suggested the formation of some 3,4-units. The carbons from the main chain of the polymer appear at approximately δ = 139 ppm. The retention of peaks in the polymer (δ = 131 and 124 ppm) show that the unsaturation (C3=C4, C16=C17, and C25=C26) is preserved with polymerization taking place at the C22=C23 and C29=C30 positions with 1,4-PMy produced. The signals of −CH=C– are split into two peaks at 125.6 and 124.5 ppm (C4, C12, C16, C25, and C29) with the alkenyl carbons from 1,2- and 3,4-units appearing as a signal peak at 109 ppm. Different amounts of 1,4*-cis* and 1,4*-trans* contents can be assigned using ^13^C NMR (Figure S5(d)). Both 1,4*-cis-* and 1,4-*trans-*units in PMy were observed at 131.1 ppm (C26) and 131.3 ppm (T26), respectively. All of the other carbons appear between 17.7 and 50.0 ppm with significant overlap (Figure S5).

Solvents with different polarities were utilized for the anionic polymerization of myrcene including cyclohexane, diethyl ether, dioxane, and THF in addition to two biorenewable “green” solvents, 2-methyltetrahydrofuran (2-MeTHF) and squalane, and used without purification so as to limit the energy requirement of the processes. The highest amount of 1,4*-cis* PMy was achieved by anionic polymerization in both cyclohexane and squalane (∼83%), while ∼56% of 1,4*-trans* PMy was obtained by radical emulsion polymerization. The highest amount of 3,4-PMy was observed in dioxane as the solvent in anionic polymerization. The two “green” solvents (2-MeTHF and squalane) gave similar stereochemistries to anionic polymerization in THF and cyclohexane, respectively, as expected and thus could be used interchangeably with the traditional conventional solvents (Table 1; NMR spectra are in the Supporting Information).

Size exclusion chromatography (SEC) revealed a monomodal mass distribution with a tailing/structure which might result from the residual unsaturated alkenyl bonds leading to dimerization/cross-linking during polymerization (Figure S8). The level of reaction decreases with decreasing solvent polarity (e.g., cyclohexane < THF). Anionic polymerization is oxygen/air intolerant, and thus, in order to check whether oxygen is an important parameter for this dimerization/cross-linking, the polymerization of myrcene was carried out with 10% air by preinjection of air into the reaction. The “real” oxygen levels were measured using a FireStingGO2 oxygen probe of both the head space and the liquid phase. The MWt of PMy was higher with broader molar mass distributions when air was present (Figure S9(a)).There was fast consumption of oxygen, complete in 12 s, following initiator injection (Figure S9(b)), suggesting a role for oxygen in polydienes dimerization which is more noticeable as solvent polarity increases. Polar solvents generally have higher amounts of oxygen solubilities and also lead to ion pair separation during propagation. Different SEC narrow calibration standards, poly(methyl methacrylate) (PMMA), polystyrene (PS), and polyisoprene (PIP) were utilized for assessing the molar mass and dispersity of the products (Table S1). Broader molar mass distributions were observed in the two renewable solvents (*Đ* = 1.84 in squalane and *Đ* = 2.00 in 2-MeTHF vs *Đ* = 1.24 in THF) with relatively higher MWts at 16,000 g/mol (vs 13,500 g/mol, targeted DP_n_ = 100). We consider this to be due to the presence of undesirable impurities present in the undistilled solvents which were observed by both ^1^H NMR and GC analyses (Figures S10 and S11, respectively). We note that the use of unpurified solvents was intentional as the target was to useful products using the most undemanding reaction conditions.

Different volume ratios of [My]:[THF] resulted in a change in the amount of 1,2-PMy produced. Decreasing the concentration of myrcene, from *V*_My_:*V*_THF_ 2:1 to *V*_My_:*V*_THF_ 1:4, lead to a slightly high concentration of 1,2 PMy from 2% to 3%, respectively (Figure S12; Table 1, entries 4–8). Following these conditions, kinetics studies were carried out to determine the stereochemistry at as close to full conversion as possible noting that as monomer is converted to polymer the polarity of the reaction medium is constantly changing. Full monomer conversion was obtained ∼10 min (>99% by ^1^H NMR) (Figure S13). Polymerization of myrcene (DP_n_ = 50) at *V*_My_:*V*_THF_ = 1:2 resulted in an allowed reaction exotherm reaching 53 °C prior to cooling to ambient temperature with an accompanying color change from orange to dark brown.

**Figure 2:**
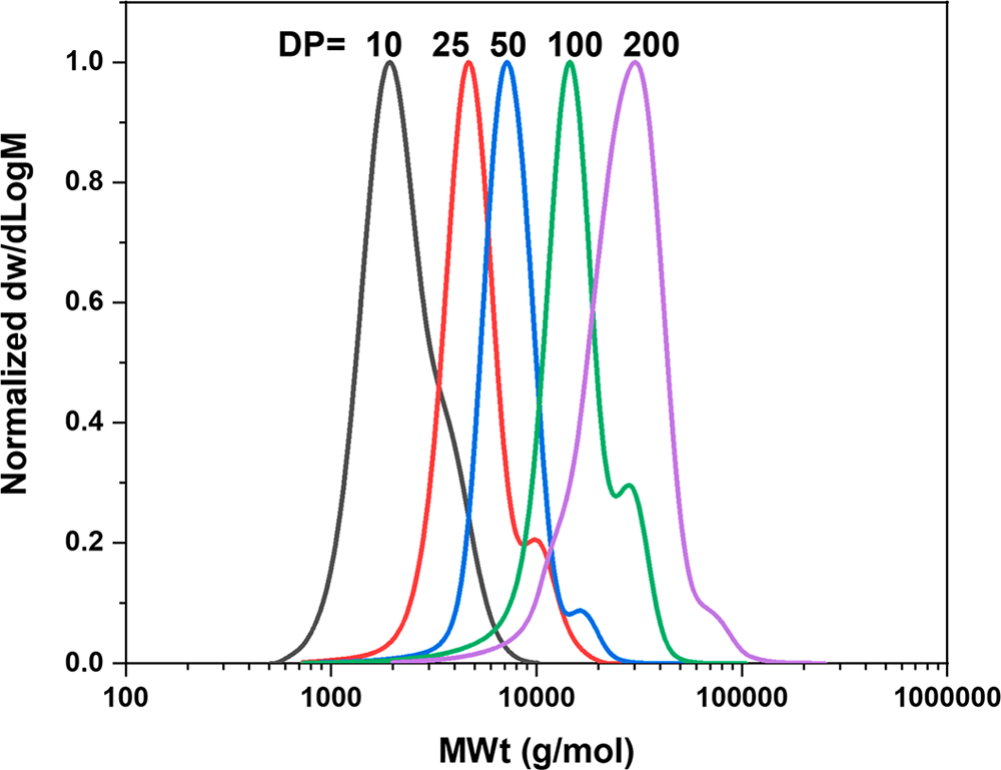
THF-SEC-derived molar mass distributions of polymyrcene for various DP_n_ values (10, 25 50, 100, and 200) synthesized by anionic polymerization in THF. Conditions: [*n*-BuLi]:[myrcene] = 1:DP, *V*_My_:*V*_THF_ = 1:2 (Table 1, entries 3–4 and 9–11) (R2, Q17).

A range of molar masses were targeted using a ratio of [My]:[*n*-BuLi] of [DP]:[1]. Full monomer conversions (>99%) and relatively narrow molar mass distributions (*Đ* < 1.28) were achieved (Figure 2). Noticeably, SEC traces exhibited a shoulder to high mass attributed to some dimerization. Increasing the targeted molar mass resulted in a decrease in the dimerization; however, this is possibly due to decreased resolution in the GPC trace as we move to higher molar mass. This dimerization seems to be due to allowing the reaction to exotherm and was considered to be acceptable for the purposes of this study and the targeted application for use in industrial dispersants where branching can be very desirable as long as synthesis consistently gives identical products. The stereochemistries of the polymers were not affected by these side reactions.

**Figure 3:**
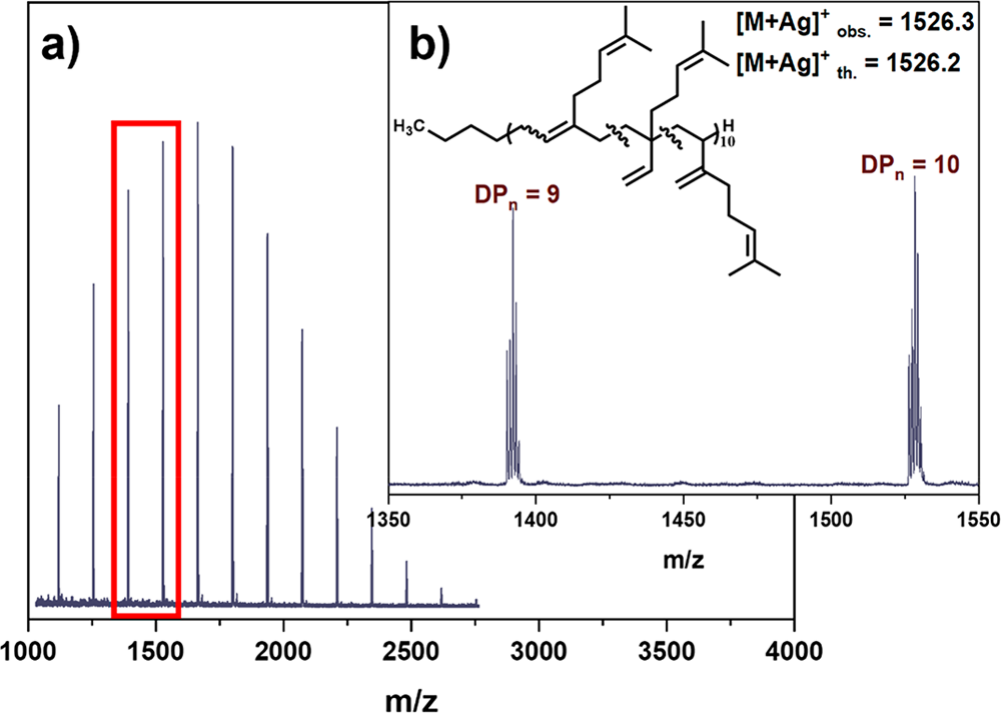
(a, b) MALDI-ToF-MS spectra for methanol quenched (R2, Q10) PMy_10_.

To confirm the polymer end groups, matrix-assisted laser desorption/ionization time-of-flight mass spectroscopy (MALDI-ToF-MS) was employed for PMy_10_, using *trans-*2-[3-(4-*tert*-butylphenyl)-2-methyl-2-propenylidene]malononitrile (DCTB) as the matrix and AgTFA as the cationization agent. A single peak distribution corresponding to H-ω-terminated polymer chains (Figure 3(a) and (b)) with a calculated mass for the butyl initiated and hydrogen terminated polymers with DP_n_ = 10 of 1526.2 Da and an observed mass = 1526.3 Da with the associated isotopic pattern expected.

Thermal analysis was used to determine the effect of different stereochemistries of PMy on the thermal stabilities of the products. The onset of decomposition temperature (*T*_d_) for PMy from all polar solvents was approximately 365 °C, with *T*_d_ approximately 10 °C lower from nonpolar solvents with a high *cis-*1,4 stereochemistry content (Figure S14). High mass loss, >99%, was observed in all PMy products > 500 °C. The glass transition temperature (*T*_g_) of PMy was at −56 °C with high 1,4-unit content, approximately 10 °C lower than the *T*_g_ with high 3,4- and 1,2-units (Figure S15).

**Figure 4:**
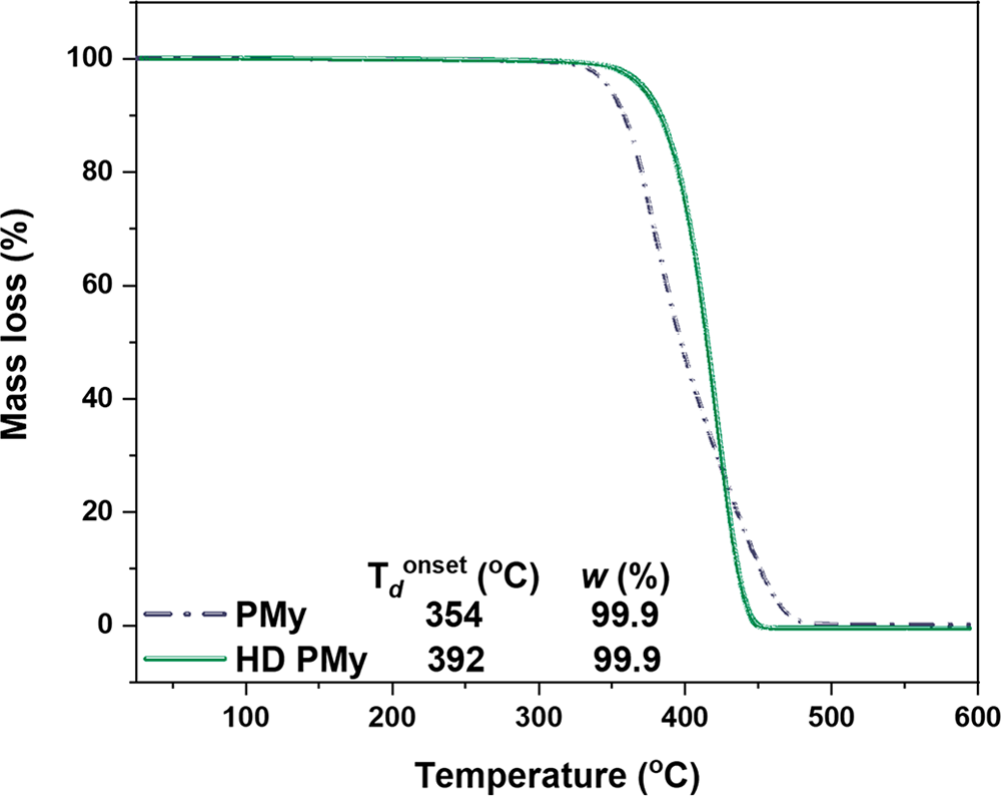
TGA thermograms of PMy_10_ and hydrogenated PMy_10_ (Table 1, entry 3) (R2, Q17).

Hydrogenated PMy was obtained via reaction with *p*-toluenesulfonyl hydrazide (5 mol equiv for each myrcene unit) in an *o*-xylene solution under reflux at 144 °C for 6 h.^67^ The resulting hydrogenation was high (>93%), confirmed by ^1^H NMR (Figure S16). TGA data showed increased thermal stability of the hydrogenated PMy as we expected (Figure 4). The decomposition temperature of the hydrogenated PMy was ∼390 °C, 40 °C higher than the *T*_d_ of the unsaturated PMy (*T*_d_ ∼ 350 °C). Furthermore, the *T*_g_ of hydrogenated PMy is approximately 15 °C higher than unsaturated PMy, *T*_g_ ∼ −64 °C and ∼ −50 °C, respectively (Figure S17).

### Regioselective Photoinduced Postmodification of PMy

The aim of this work was to produce polymers that can act to disperse particulate matter in hydrocarbon-based fluids such as those used in automotive lubrication applications via postpolymerization introduction of polar chemical functionality. Partial functionalization via a thiol–ene click reaction was investigated. It is noted that most previous studies have investigated thiol–ene full functionalization utilizing an excess of thiol with respect to total alkenyl groups resulting in little or no cross-linking as all double bonds react with the thiol. However, as all alkenyl groups are consumed, this leads to completely new materials with very different material properties to the parent polymers and usually much increased polarity. In this work, we were interested in producing functionalized PMy and hydrogenated functionalized PMy while retaining properties (solubility, thermal, and mechanical) of the parent polymers. Thus, thiol–ene radical addition modification reactions were carried out using a molar excess of alkenyl groups with respect to the thiol. Reaction of PMy, as prepared by anionic polymerization with methyl thioglycolate (MTG) in a toluene solution followed a similar procedure to that reported by Matic and Schlaad.^18^  2,2-Dimethoxy-2-phenylacetophenone (DMPA) was used as a photoinitiator for the generation of thiyl radicals under UV exposure (λ = 365 nm) at ambient temperature. PMy was functionalized by a reaction with a range of thiols, 3-mercaptopropionic acid (MAC), 2-mercaptoethanol (MET), 3-mercapto-1-hexanol (MH), and 1-thioglycerol (THG), aimed at selectively targeting the vinyl group from the 1,2-unit. Partially functionalized PMy was further modified by hydrogenation so as to remove all remaining unsaturation from the product to improve stability (Scheme 2).

Different concentrations of MTG (0.025, 0.050, and 0.075 mol equiv with respect to each myrcene repeat unit) were used. The polymers were investigated by ^1^H NMR, and substitution at the 1,2-vinyl groups was more prevalent when compared to the other types of alkenyl groups. The signals from the 1,2-units at 5.4 ppm disappeared, while the integrals from the 1,4- and 3,4-units remained unchanged. The peaks assigned to the methyl thioglycolate adduct appear at 3.4–3.7 ppm, confirming the successful thiol addition to the less substituted 1,2- vinyl groups (Figure S18).

**Figure 5:**
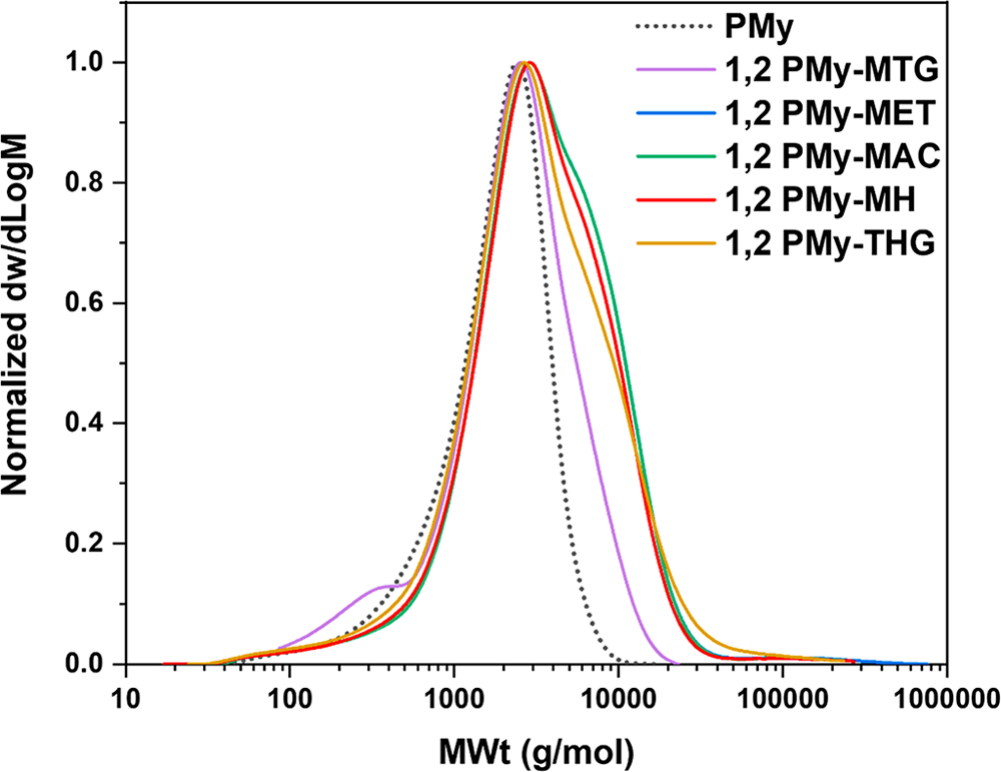
THF-SEC traces of PMy (M_n,PMy_ = 1100 g mol^–1^) (R2, Q6) and thiol–ene derivatives, narrow molar mass PMMA as standards (Table 2, entries 1–5) (R2, Q12).

**Figure 6:**
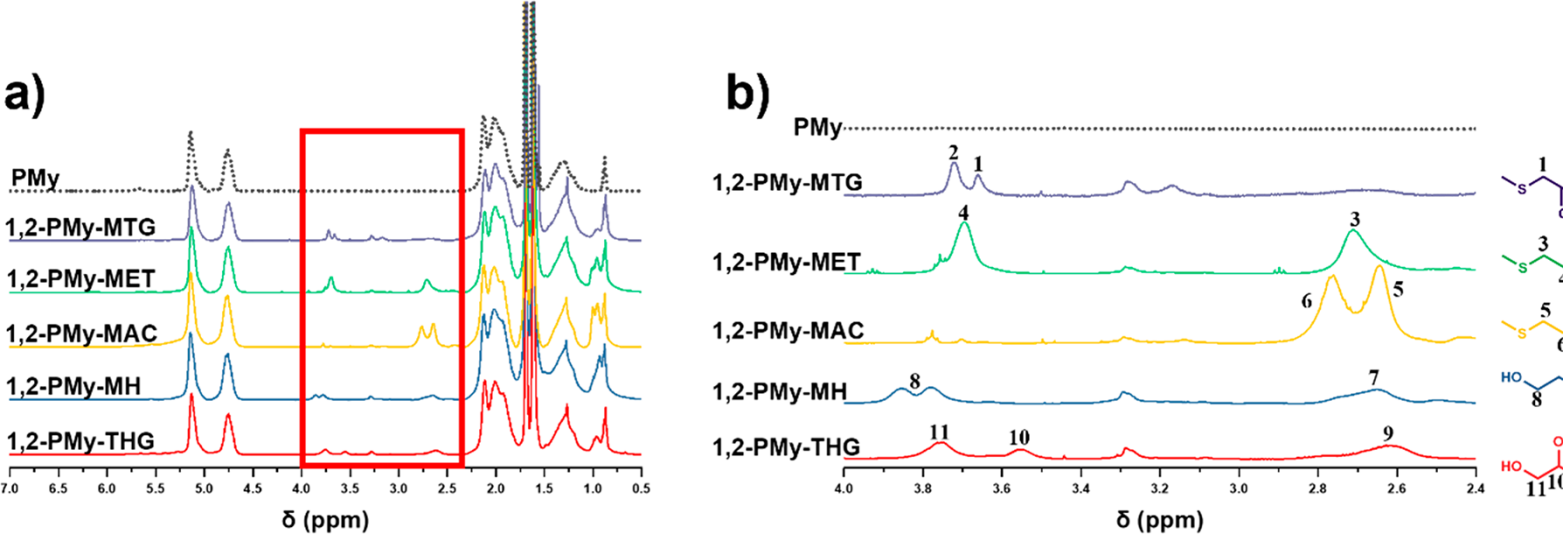
^1^H NMR spectrum (500 MHz, CDCl_3_-*d*) of the 1,2-PMy postmodified with various thiols.

Subsequently, the thiol was changed with reactions proceeding differently in each case (Table S2). Methyl thioglycolate (MTG) (Table S2, entry 1) is rather active, achieving 100% functionalization of the 1,2-vinyl bonds. Conversely, under identical conditions (Table S2, entries 2, 6, 11, and 16), successful hydrophilic thiol addition on the 1,2-vinyl groups was observed even more than 48 h. It has been previously reported that these coupling reactions are less favorable when the higher, and indeed stoichiometric excess, amount of thiols are used.^18^ Increasing the molar equivalents of the thiols with respect to the unsaturation resulted in full conversion of alkenyl groups over a shorter time period minimizing coupling (Table S2, entries 5, 10, 15, and 19). In that case, the change of integral of the peaks from the 1,4-units shows that hydrophilic thiols react with the trisubstituted vinyl groups of PMy in addition to the vinyl groups from the 1,2-units. In all instances, the peaks corresponding to two substituted alkenyl groups (3,4-units) and main chain double bonds remain similar which is in accordance with the literature.^18^ However, SEC traces showed shoulders appearing for functionalized PMy at higher molar mass indicating a degree of chain coupling (Figure 5). This is ascribed to dimerization of the initial polymer during the functionalization (Figure S19) to compare two batches of functionalized PMy. The thiol addition to the vinyl groups occurred under different conditions (Table S2, entries 1 and 11).^68^ The complete disappearance of the peaks attributable to the pendent vinyl group of 1,2-PMy and the successful attachment of the different types of thiols was verified by ^1^H NMR. The characteristic signals of methylene protons of thiols were observed as −SCH_2_COOCH_3_ (3.20 ppm), −SCH_2_CH_2_OH (2.68–3.60 ppm), −SCH_2_CH_2_COOH (2.60–2.80 ppm), −SCH(CH_2_CH_2_CH_3_) (CH_2_CH_2_OH) (3.80 ppm), and −SCH_2_CH(OH)CH_2_OH (2.67–3.87 ppm) (Figure 6). The products of the functionalized PMy were investigated by ^13^C NMR. New peaks appeared assigned to the carbonyl from the addition of methyl thioglycolate and the methyl group at 170–174 ppm (Figure S20, m) and 52 ppm (Figure S20, k), respectively. Peaks attributed to the methylene carbon of 2-mercaptoethanol were identified at 56 ppm (Figure S20, n) indicative of the desired product. The peak at 178 ppm (Figure S20, o) is assigned to C=O originating from 3-mercaptopropionic acid, while the peak at 58 ppm (t) is assigned to the methylene carbon from 3-mercapto-1-hexanol. The resonance of methine and methylene carbon of 1-thioglycerol are seen at 62 and 73 ppm, respectively (Figure S20).

The structure of the functionalized PMy was also consistent with the FTIR spectra. The stretching ν_OH_ is observed at 3365 cm^–1^ following the incorporation of MET, MH, and THG into PMy. Similarly, the characteristic ν_CO_ from the carbonyl groups of MTG and MAC are observed at 1735 and 1710 cm^–1^ respectively, indicating the addition of the thiols to PMy. Moreover, the ν_OH_ peaks at 3490 cm^–1^ and ν_CO_ from the acid at 1710 cm^–1^, which are higher when compared in Figure S21 and support the reaction of the trisubstituted vinyl groups with thiols via addition to the monosubstituted 1,2-vinyl groups (Figure S22).

It is noted that although thiol glycolate functionalized PMy (*M*_n_ = 1200 g/mol) molar mass and molar mass distribution are similar to the precursor (*M*_n_ = 1100 g/mol), the hydrophilic thiol functionalized PMy molar masses appear higher than expected with a broadening of dispersity and evidence of for example, dimer and trimer formations accompanying the PMy thiol addition (Table 2). Thus, although polymer–polymer reactions with a buildup of molar mass were observed, cross-linking into insoluble products was avoided. This has been previously observed with substoichiometric amounts of thiol relative to alkenyl groups. Partially thiol–ene functionalized PMy was further modified by hydrogenation via reaction with *p*-toluenesulphonyl hydrazide. Nearly all of the unreacted vinyl groups are consumed, whereas the proton signals from the thiol–ene functionalization remain at approximately 3.6 and 1.8 ppm for MET, THG, and MTA and at 2.69 ppm for MAC (Figure S23), thus leading to branched saturated hydrocarbons with a range of polar functionalization.

**Table 2:** THF-SEC Results of the Thiol-Functionalized PMy

Entry	Thiols	[PMy]:[thiol]:[PI]	*M*_n,SEC_^a^ (g/mol)	*Đ*^a^	F.D. of 1,2-unit^b^ (%, NMR)	F.D. of 1,4-unit^b^ (%, NMR)
1	MTG	1:0.075:0.05	1200	2.60	>99	–
2	MET	1:1:0.05	1660	3.90	>99	3.2
3	MAC	1:0.5:0.05	1750	3.10	>99	9.7
4	MH	1:1:0.05	1660	3.30	>99	2.4
5	THG	1:1:0.05	1530	3.80	>99	0.8

a Determined by THF-SEC analysis and expressed as molar mass equivalents to PMMA narrow molar mass standards, *M*_n,PMy_ = 1100 g mol^–1^.

b Conversion was calculated via ^1^H NMR using CDCl_3_-*d* as the solvent.

## Conclusions

In summary, PMy has been prepared with different stereochemistries using both radical and anionic polymerizations. The effect of changing the solvent on stereochemistry in anionic polymerization has been investigated and utilized to give up to 4% 1,2-addition with relatively high reactive vinylic groups with regard to reaction with thiols. Both polar and nonpolar renewable solvents have been used and compared with conventional petrochemical-derived solvents. This has allowed for varying the type and amounts of the different pendent double bonds. The different alkenyl groups are shown to have different reactivities toward postpolymerization modification, with the vinylic 1,2-units, which are presented at low levels via anionic polymerization in polar solvents, being available for selective reaction by a photochemical thiol–ene click reaction. Several different hydrophilic/functional thiols were effectively added to PMy by using substoichiometric amounts to introduce polarity and heteroatoms, and although polymer–polymer reactions were observed, cross-linking and formation of nonsoluble gels were avoided. Subsequently, methyl thioglycolate was introduced into PMy with a combination of *cis-/trans-*1,4-, 1,2-, and 3,4-units to investigate the relative reactivity of the alkenyl groups; then, PMy thiol derivatives were further modified by hydrogenation in order to improve their thermal stabilities toward oxidation.

## Supporting Information Available

More detailed figures, schemes, and tables including NMR, GPC, TGA, DSC, FTIR, and GC-FID (PDF)
