# Supplementary material for: Polymerization of Myrcene in Both Conventional and Renewable Solvents: Postpolymerization Modification via Regioselective Photoinduced Thiol–Ene Chemistry for Use as Carbon Renewable Dispersants
Source: ACS Sustain Chem Eng. 2022 Jul 11;10(29):9654–64. doi: 10.1021/acssuschemeng.2c03755 (PMC9344384; doi:10.1021/acssuschemeng.2c03755)
Supplement: Supplementary file 1 — sc2c03755_si_001.pdf [file sc2c03755_si_001.pdf]

Supplementary Information

Polymerization of Myrcene in Both Conventional and Renewable Solvents: Postpolymerization Modification via Regioselective Photoinduced Thiol–Ene Chemistry for Use as Carbon Renewable Dispersants

Jirui Zhang,[a] Cansu Aydogan,[a] Georgios Patias,[a] Timothy Smith[b], Lucas Al-Shok,[a] Huizhe Liu,[a] Ahmed M. Eissa,[a] and David M. Haddleton[a]*

[a] Department of Chemistry, University of Warwick, Gibbet Hill, Coventry, CV4 7AL, UK

[b] Lubrizol Ltd, Nether Lane, Hazelwood, Derbyshire DE56 4AN, UK
Email: d.m.haddleton@warwick.ac.uk

Number of pages: 49

Number of figures: 54

Number of tables: 2

Number of schemes: 5

Additional experimental details, schemes, spectra and chromatograms.

Supporting Figures:

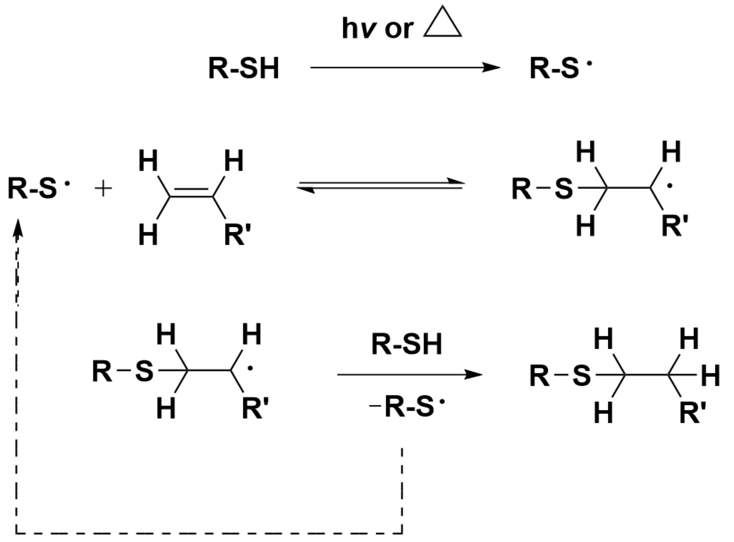

Scheme S1. General mechanism for the radical thiol-ene reaction.

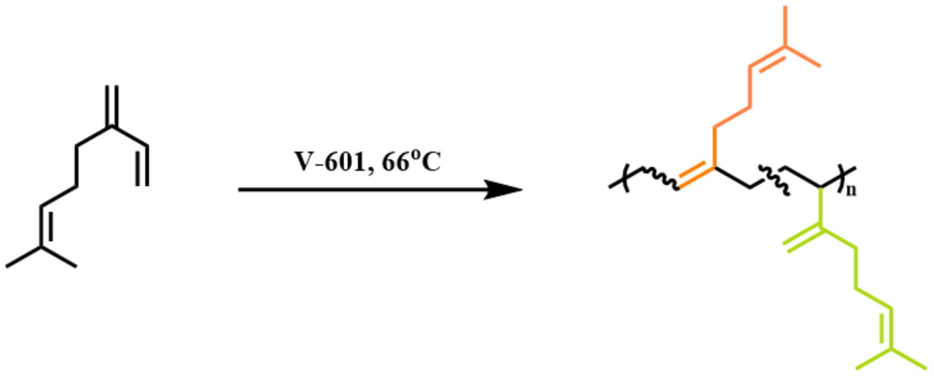

Scheme S2. Reaction scheme of free radical polymerisation of myrcene, 2.5 wt% of V-601 as initiator in bulk.

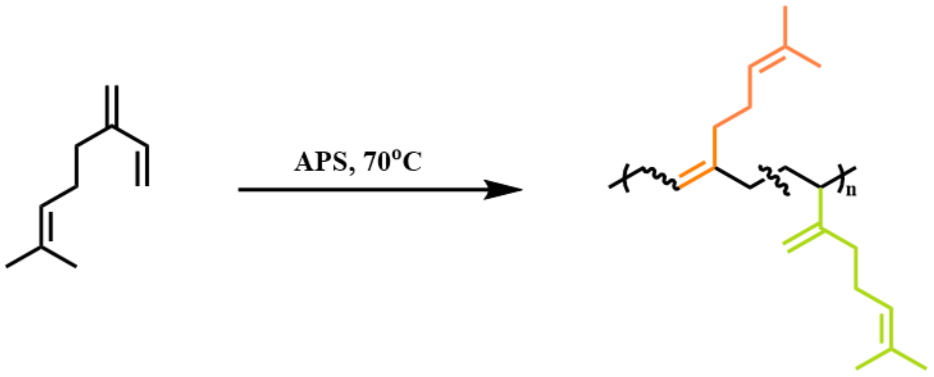

Scheme S3. Reaction scheme of the emulsion polymerisation of myrcene. Conditions: 28.5 wt% of myrcene in aqueous, APS, SDS and 1-dodecanthiol used as initiator, surfactant and chain transfer agent, respectively.

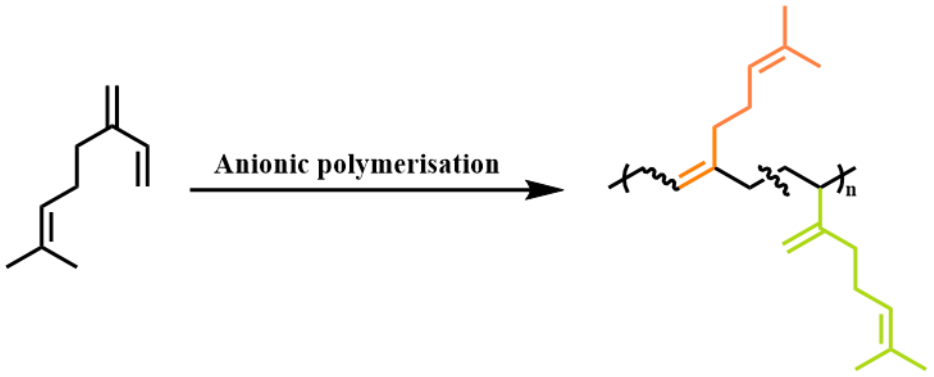

Scheme S4. Reaction scheme of the anionic polymerisation in cyclohexane or squalane of myrcene. Conditions: [My]:[*n*-BuLi] = [100]:[1] in different solvents (1:2 v/v monomer/solvent) at ambient temperature.

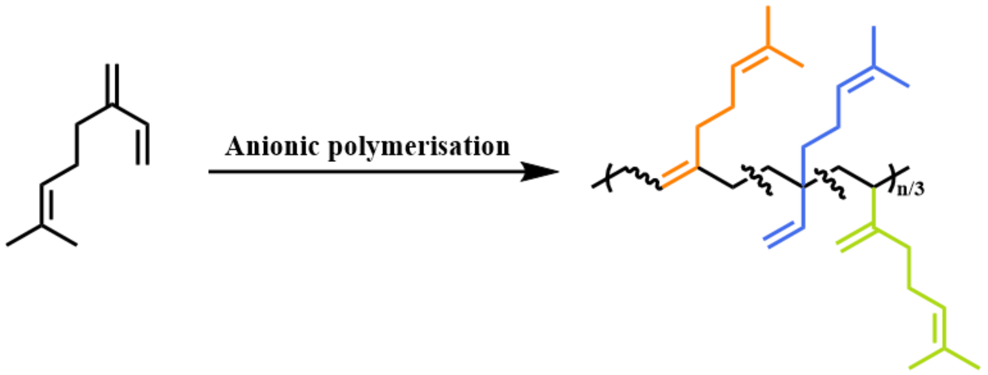

Scheme S5. Reaction scheme of anionic polymerisation in THF, 2-MeTHF, dioxane and diethyl ether. Conditions: [My]:[*n*-BuLi] = [100]:[1] in different solvents (1:2 v/v monomer/solvent) at ambient temperature.

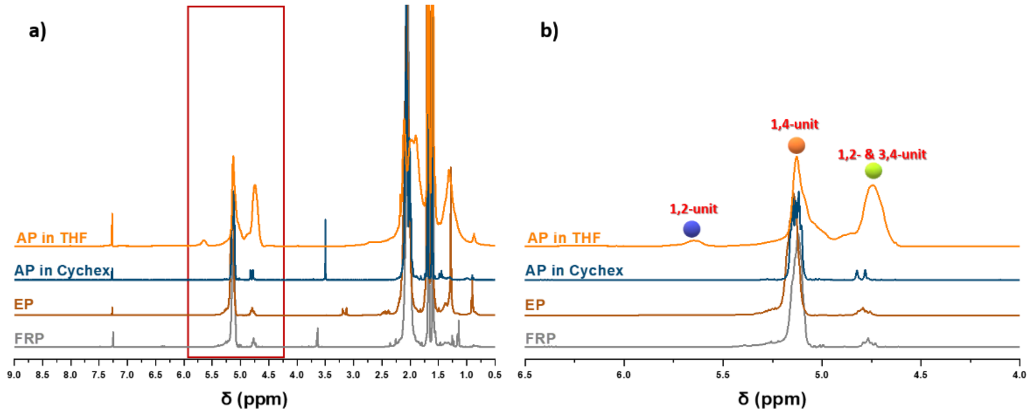

Figure S1. a). ^{1}H NMR spectrum (500 MHz, $CDCl_3$-*d*) of PMy, synthesized *via* anionic polymerisation and radical polymerisation. b) Expanded ^{1}H NMR spectrum of a) in the range of 4.0-6.5 ppm (**Table 1, entries 1, 2, 10 &12**).

*AP = anionic polymerisation; EP = emulsion polymerisation; FRP = free radical polymerisation

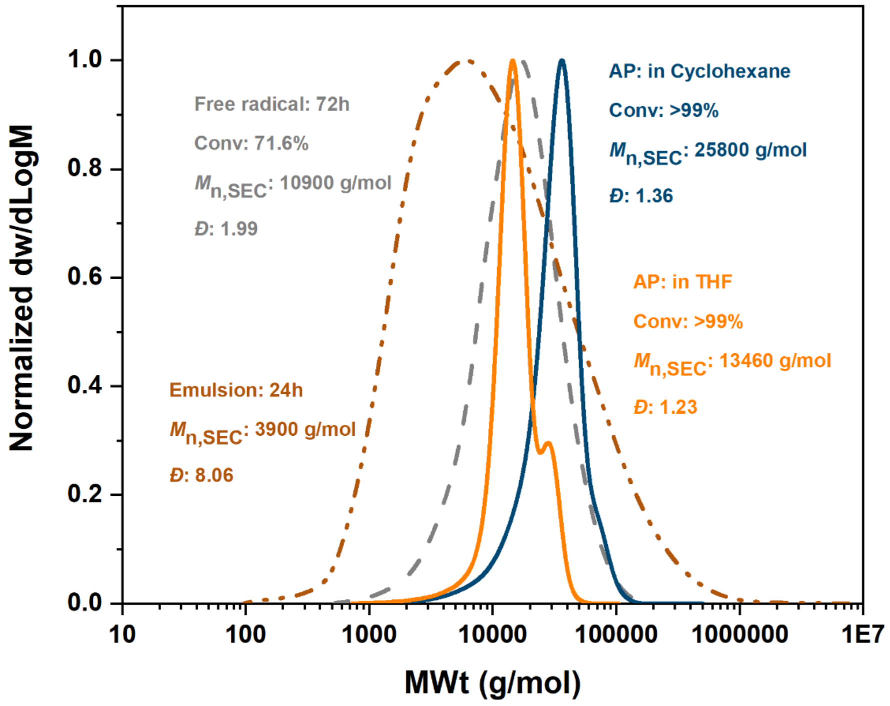

Figure S2. SEC traces of PMy from both anionic polymerisation and radical polymerisation as determined by $CHCl_3$-SEC with narrow PMMA as calibration standards (**Table 1, entries 1, 2, 10 &12**).

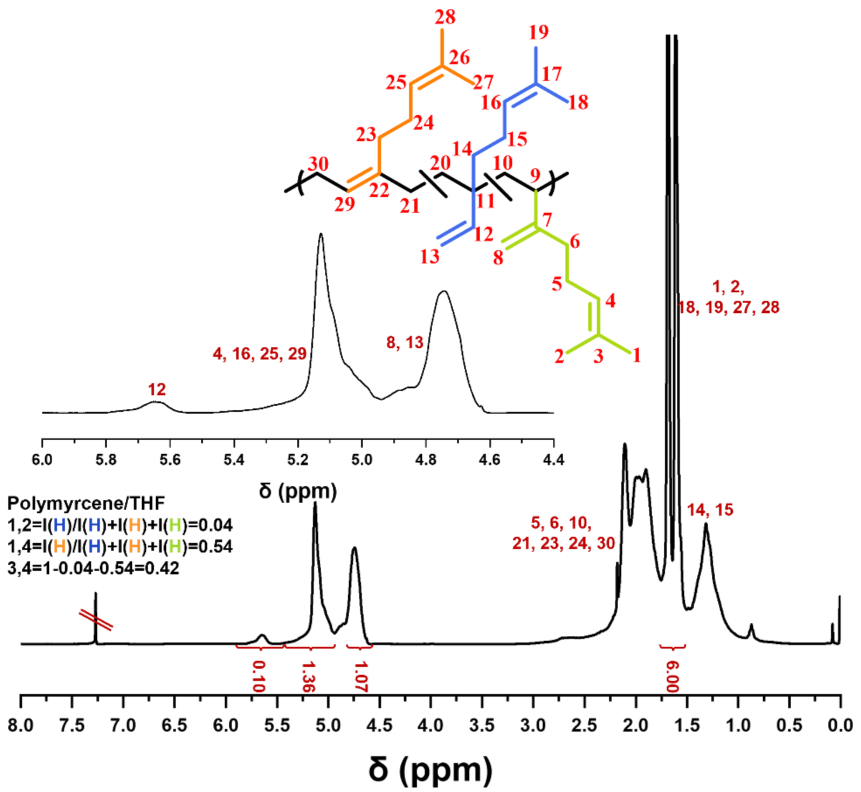

Figure S3. ^{1}H NMR spectra (500 MHz, $CDCl_3$-*d*) of PMy synthesized *via* anionic polymerisation in THF with targeted DP_n = 100, V_{My}:V_{THF} = 1:2; determination of stereochemistry (**Table 1, entry 10**).

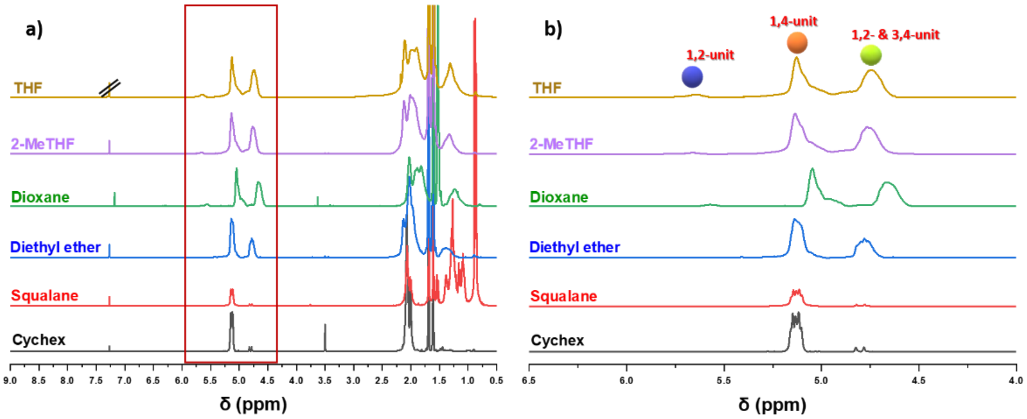

Figure S4. a). ^{1}H NMR spectrum (500 MHz, $CDCl_3$-*d*) of PMy, synthesized *via* anionic polymerisation in different solvents as marked. b) Expanded ^{1}H NMR spectrum of a) in the range 4.0-6.5 ppm (**Table 1, entries 10, 12 - 16**).

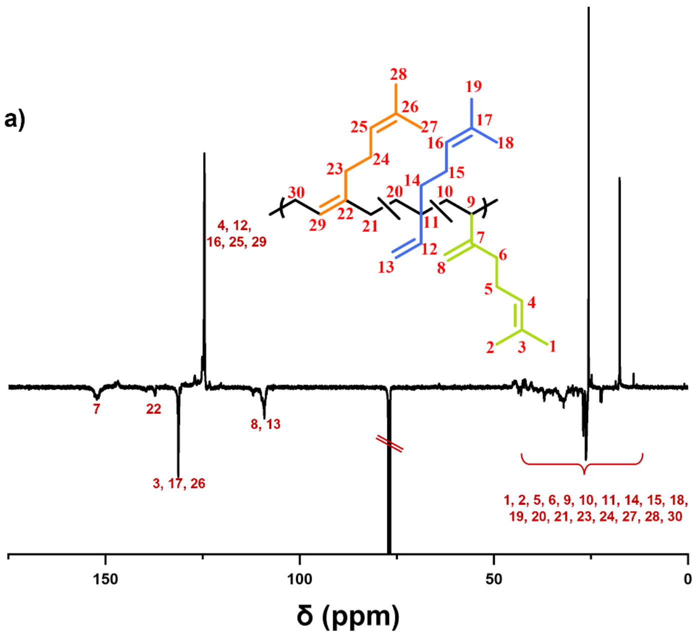
a)
4, 12,
16, 25, 29
7
22
3, 17, 26
8, 13
1, 2, 5, 6, 9, 10, 11, 14, 15, 18,
19, 20, 21, 23, 24, 27, 28, 30
150
100
50
0
δ (ppm)

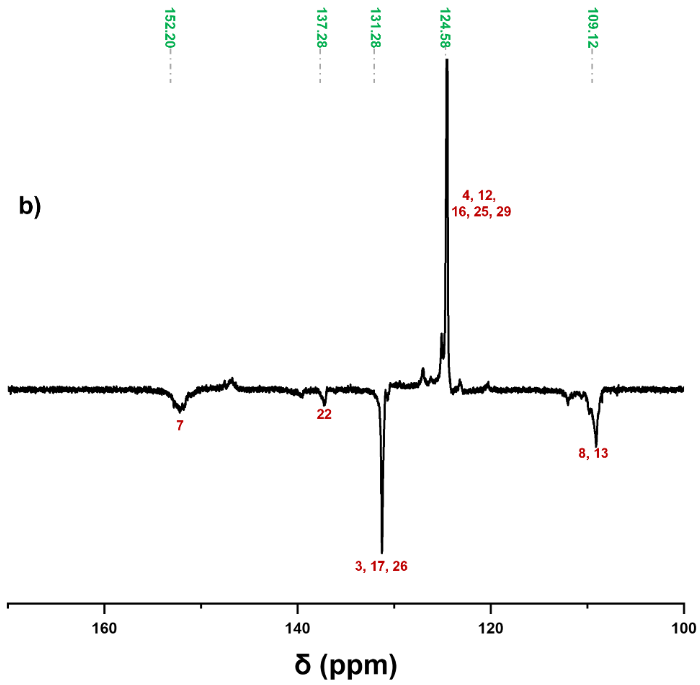
152.20
137.28
131.28
124.58
109.12
b)
4, 12,
16, 25, 29
7
22
3, 17, 26
8, 13
160
140
120
100
δ (ppm)

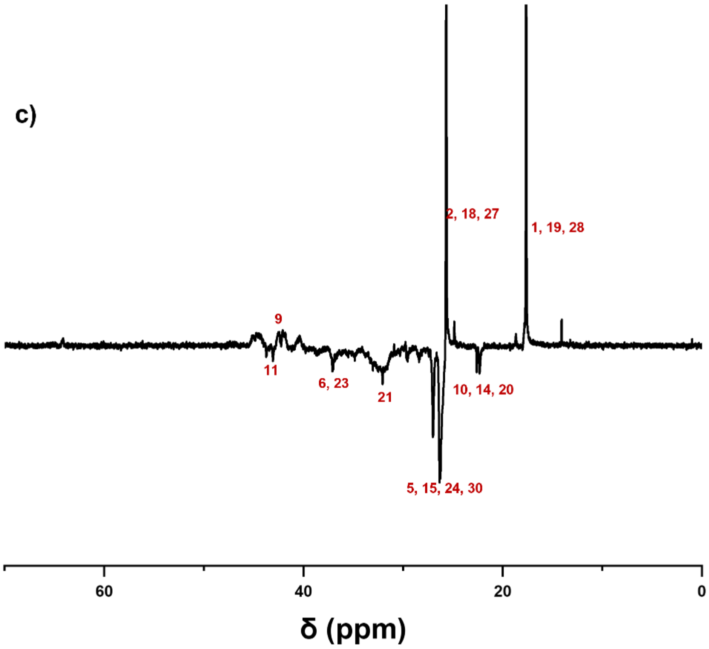

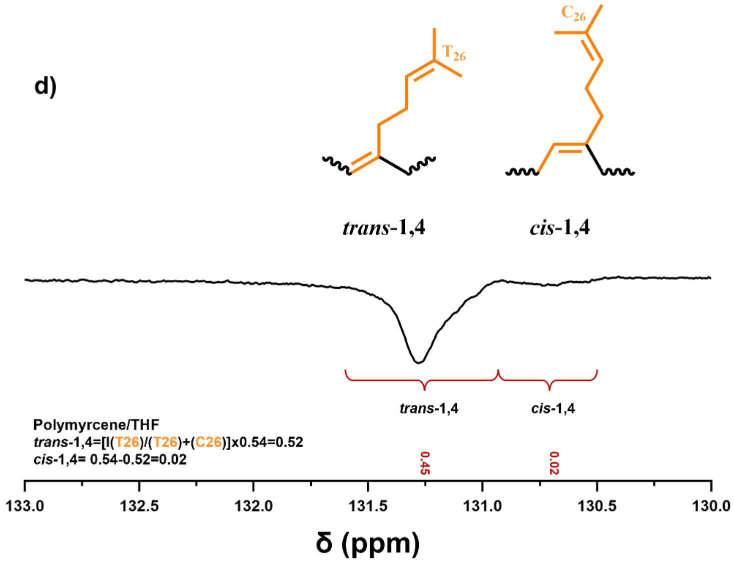

Figure S5. a). ^{13}C NMR spectrum (500 MHz, $CDCl_3$-d) of PMy synthesized *via* anionic polymerisation in THF with targeted DP_n = 100, V_{My}:V_{THF} = 1:2; determination of stereochemistry. b) Expanded ^{13}C NMR spectrum of a) in the range of 170-100 ppm. c) Expanded ^{13}C NMR spectrum of a) in the range of 70-0 ppm. d) Expanded ^{13}C NMR spectrum of a) in the range of 133-130 ppm (**Table 1, entry 10**).

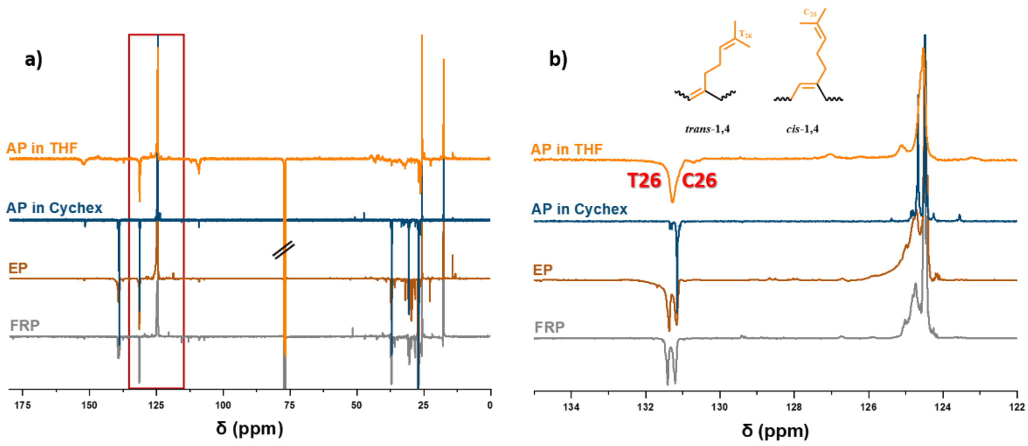

Figure S6. a). ^{13}C NMR spectrum (500 MHz, $CDCl_3$-*d*) of PMy, synthesized *via* anionic polymerisation and radical polymerisation. b) Expanded ^{13}C NMR spectrum of a) in the range of 122-135 ppm (**Table 1, entries 1, 2, 10 &12**).

*AP = anionic polymerisation; EP = emulsion polymerisation; FRP = free radical polymerisation in solution

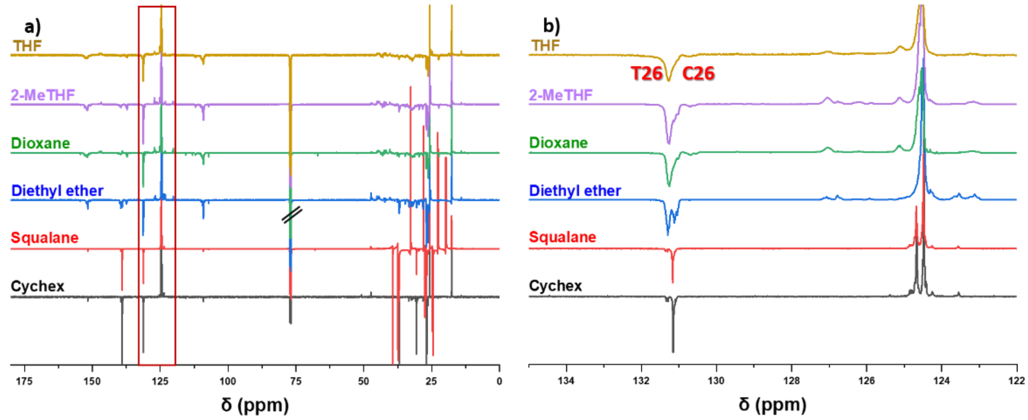

Figure S7 ^{13}C NMR spectrum (500 MHz, $CDCl_3$-*d*) of PMy, synthesized *via* anionic polymerisation and radical polymerisation. b) Expanded ^{13}C NMR spectrum of a) in the range of 122-135 ppm (**Table 1, entries 10, 12 - 16**).

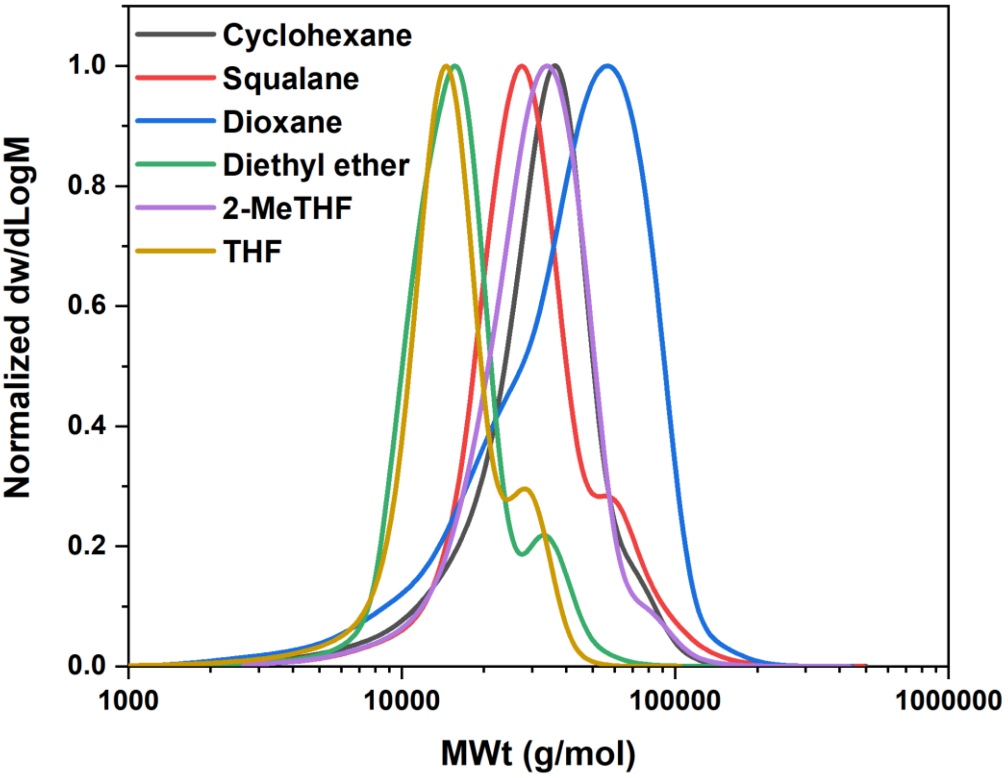

Figure S8. SEC traces of PMy, synthesized *via* anionic polymerisation in different solvents, determined by $CHCl_3$-SEC analysis (**Table 1, entries 10, 12 - 16**).

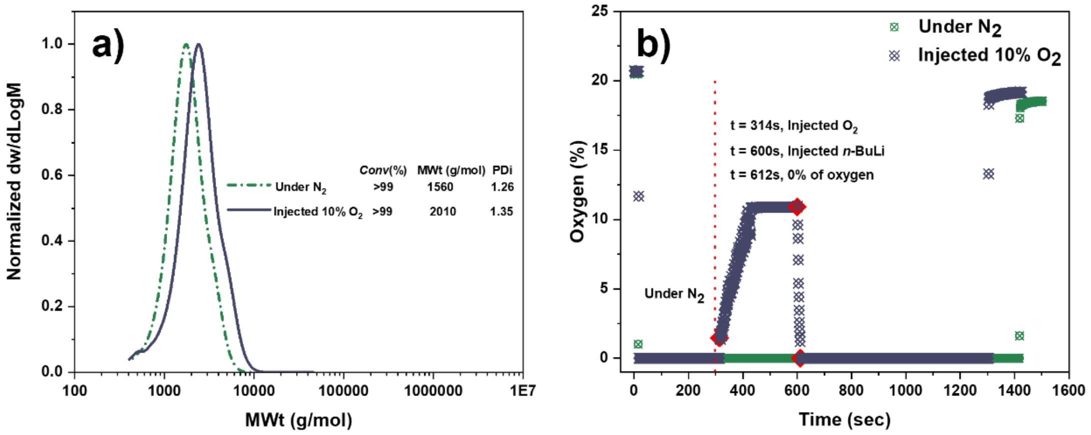

Figure S9. a). THF-SEC traces of PMy synthesized *via* anionic polymerisation with (& without) oxygen. b). graphical illustration of the oxygen consumption for the anionic polymerisation of myrcene.

Table S1. Summary of SEC data of PMy using different narrow standards for calibrations.

Sample	$MWt_{,tho}$ (g/mol)	PMMA		PS		PIP	
		MWt (g/mol)	*Đ*	MWt (g/mol)	*Đ*	MWt (g/mol)	*Đ*
PMy_{10}	1400	1730	1.20	1500	1.19	1300	1.20
PMy_{100}	13700	13700	1.28	11630	1.29	9990	1.27
PMy_{10}[a]	1400	1720	1.22	1480	1.21	1280	1.22
PMy_{100} [a]	13700	14300	1.25	12110	1.26	10400	1.24

[a] Purified PMy. Using THF as diluting solvent for SEC analysis.

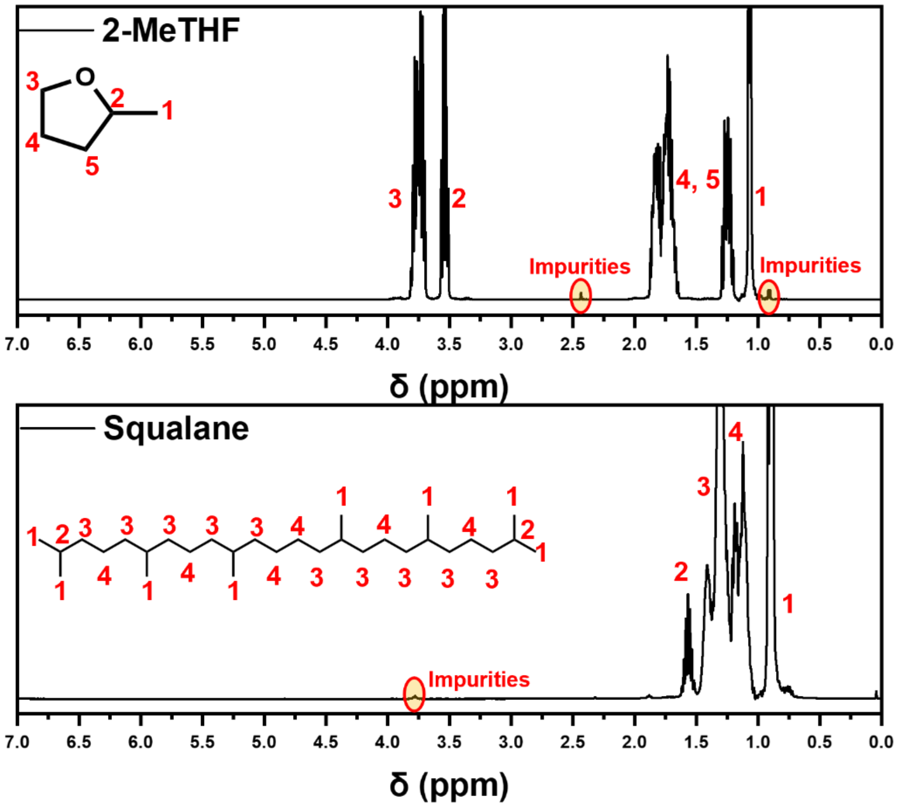

Figure S10. ^{1}H NMR spectrum (400 MHz, $CDCl_3$-*d*) of green solvents (2-methyltetrahydrofuran, top; Squalane, bottom).

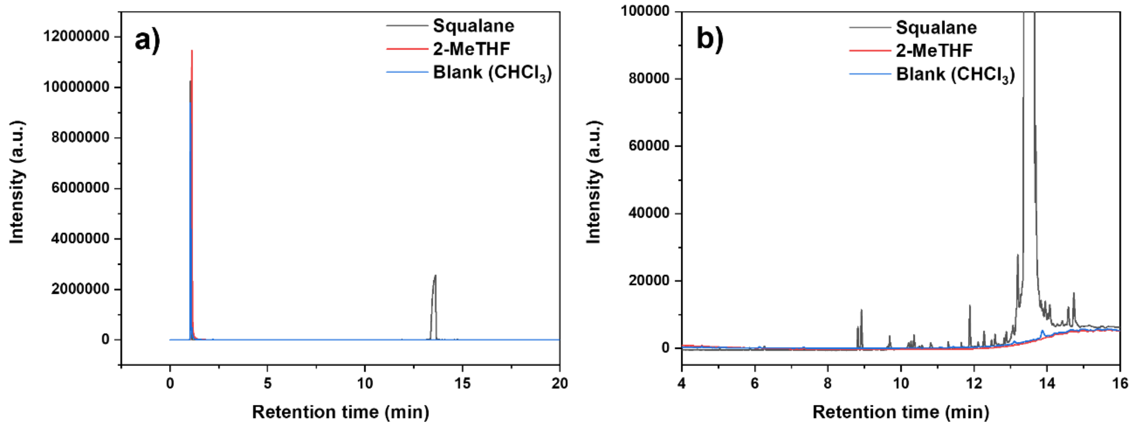

Figure S11.GC-FID spectrum of green solvents, using $CHCl_3$ (R2, Q16) as diluting solvents.

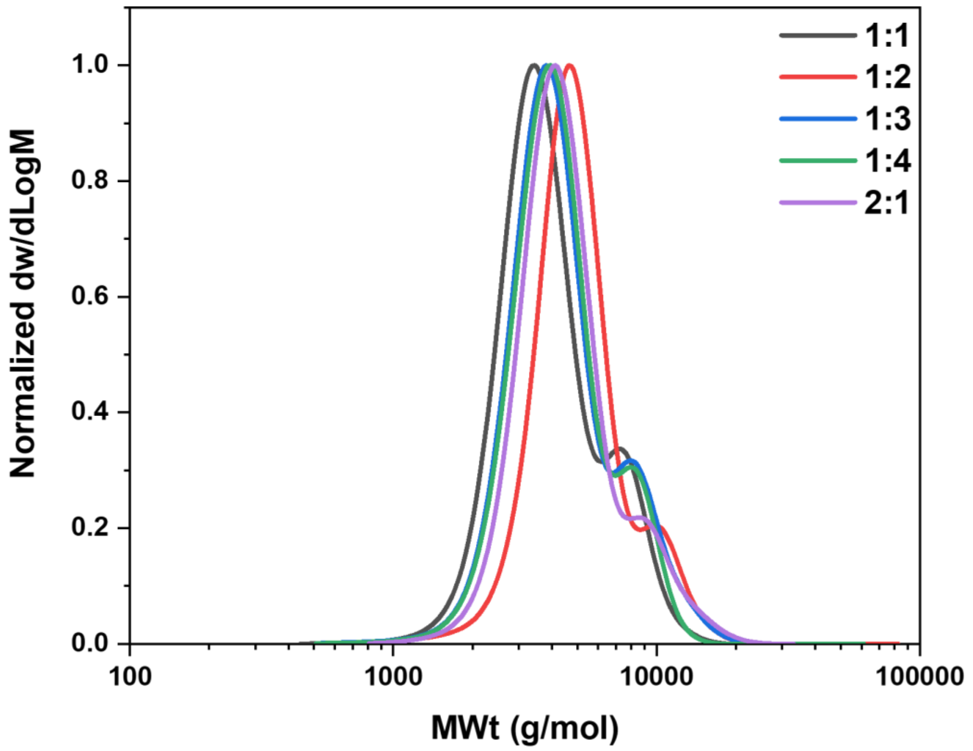

Figure S12. Molecular weight distributions of PMy synthesized *via* anionic polymerisation in THF at different solids content (caption is volume of myrcene to volume of THF), measured by $CHCl_3$-SEC, determined by $CHCl_3$-SEC analysis, using PMMA as calibration standards (**Table 1, entries 4-8**)

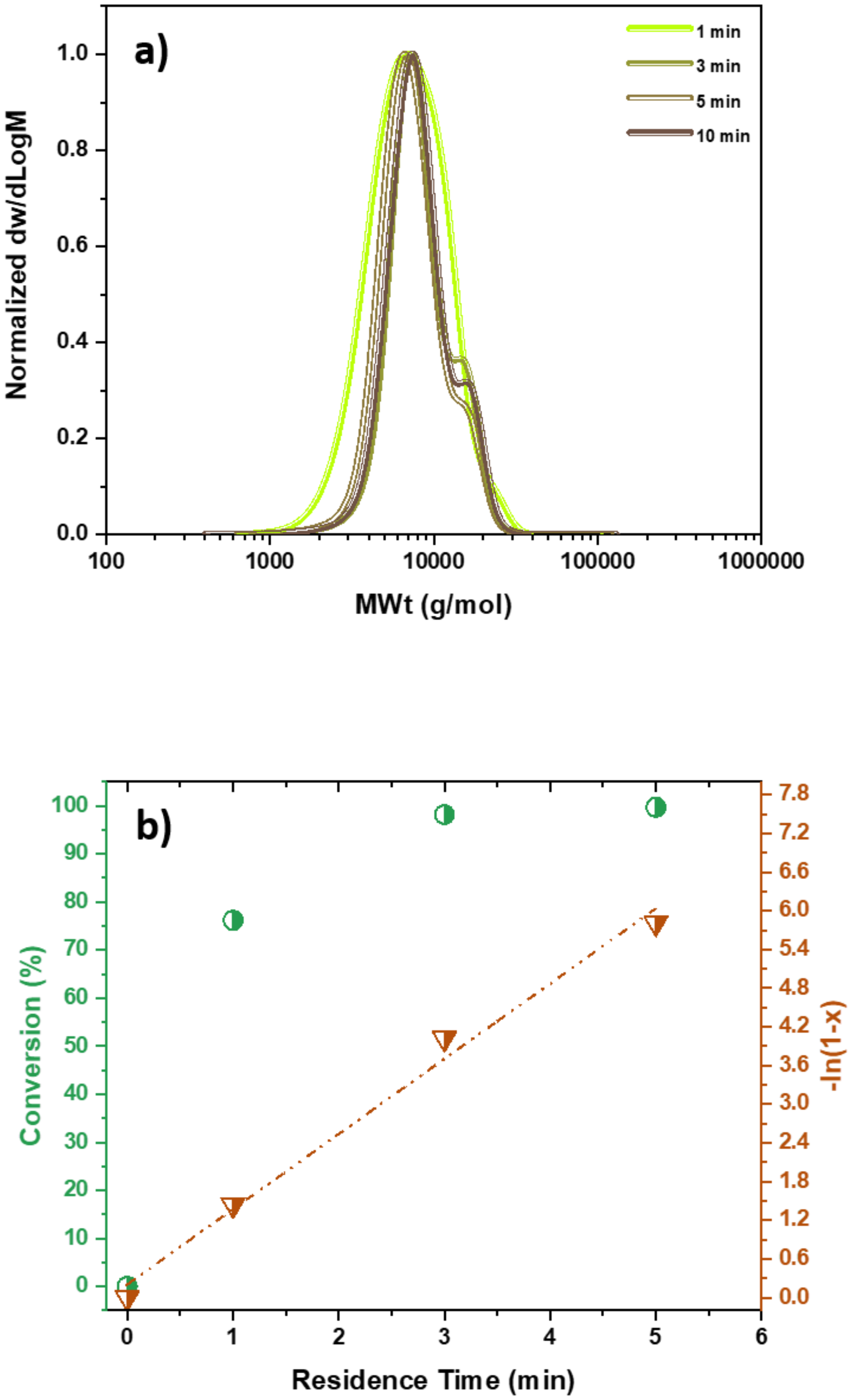
a)
1 min
3 min
5 min
10 min
Normalized dw/dLogM
MWt (g/mol)
b)
Conversion (%)
-ln(1-x)
Residence Time (min)

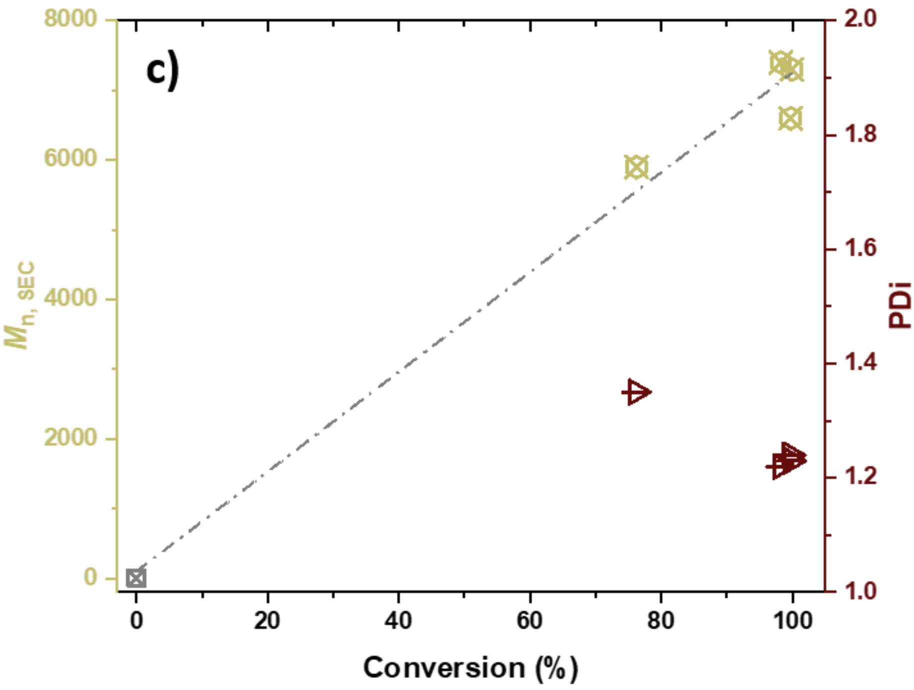

d)

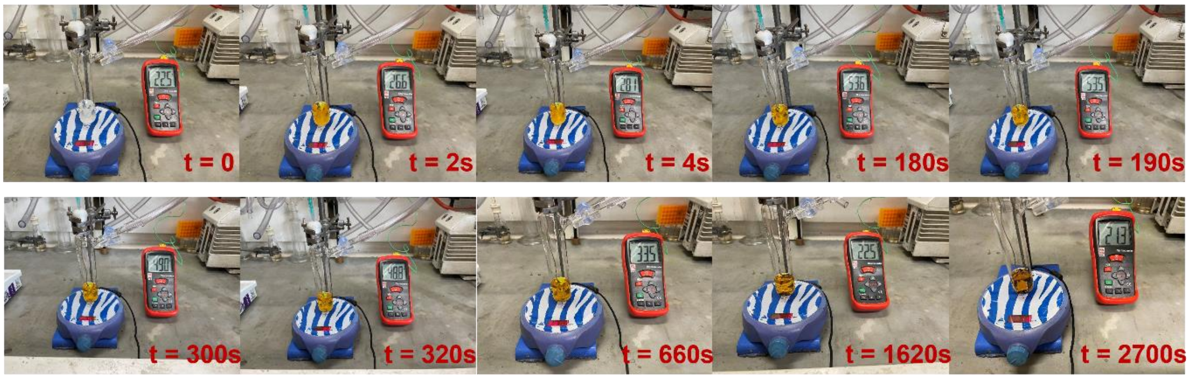

Figure S13. Kinetics studies for the anionic polymerisation of myrcene in THF with targeted DP_n = 50: a). $CHCl_3$-SEC derived molecular weight distributions showing the evolution of MWts. b). kinetics plots of –ln(1-x) (right, light brown) and conversion (left, green) versus residence time showing linear trend. c). plots of M_n versus conversion (left, light yellow) and dispersity ($Đ$) versus conversion (right, dark brown). d). Figures of polymerisation of myrcene. Conditions: [My]:[*n*-BuLi] = 50:1, V_{My}:V_{THF} = 1:2,

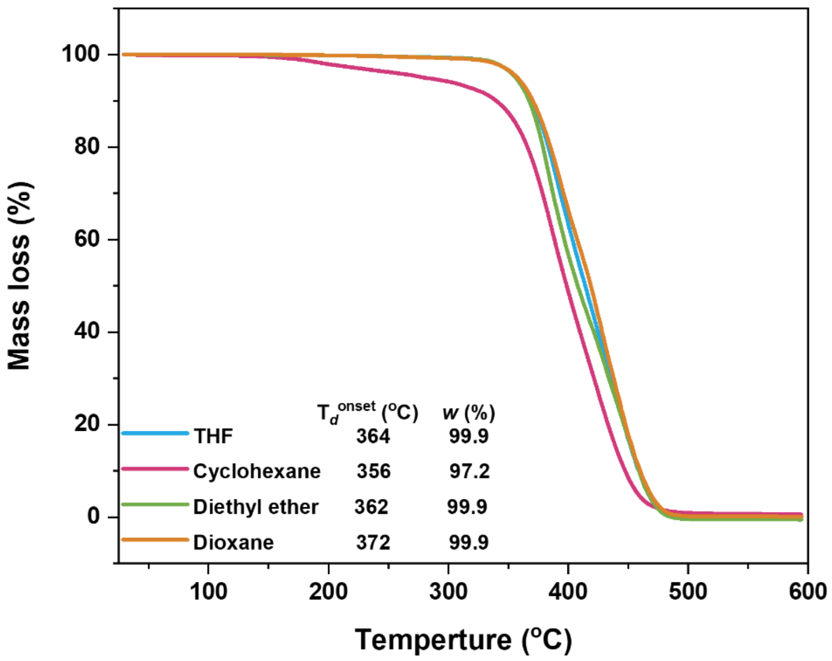

Figure S14. TGA thermomgrams of PMy in this study (Targeted DP_n = 100, **Table 1, entries 10 & 12-14**).

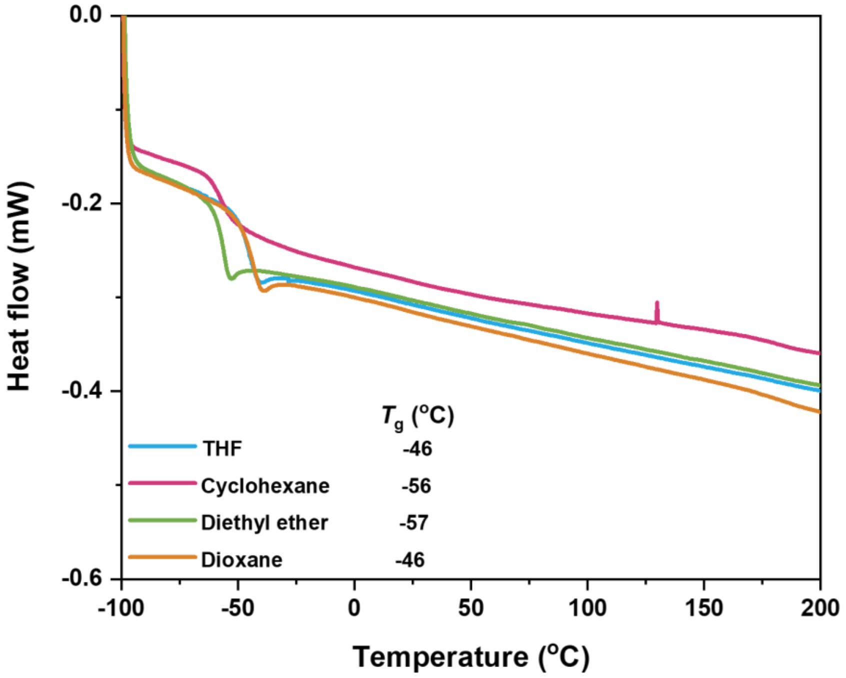

Figure S15. DSC thermograms of PMy (Targeted DP_n = 100, **Table 1, entries 10 & 12-14**)

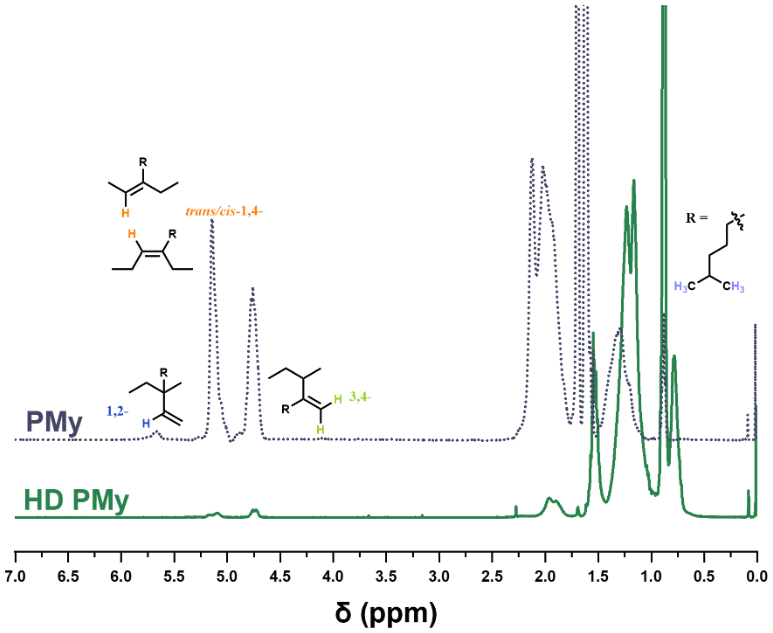

Figure S16. ^{1}H NMR (500 MHz, $CDCl_3$-*d*) of PMy_{10}hydrogenated PMy_{10}.

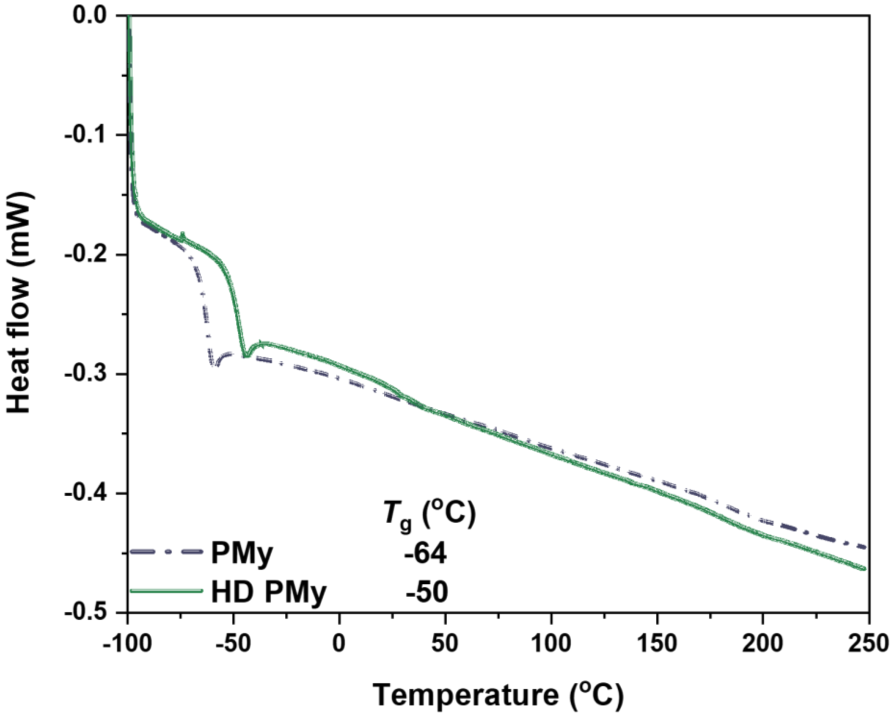

Figure S17. DSC thermograms of PMy (Targeted DP_n = 10) and hydrogenated PMy (Targeted DP_n = 10).

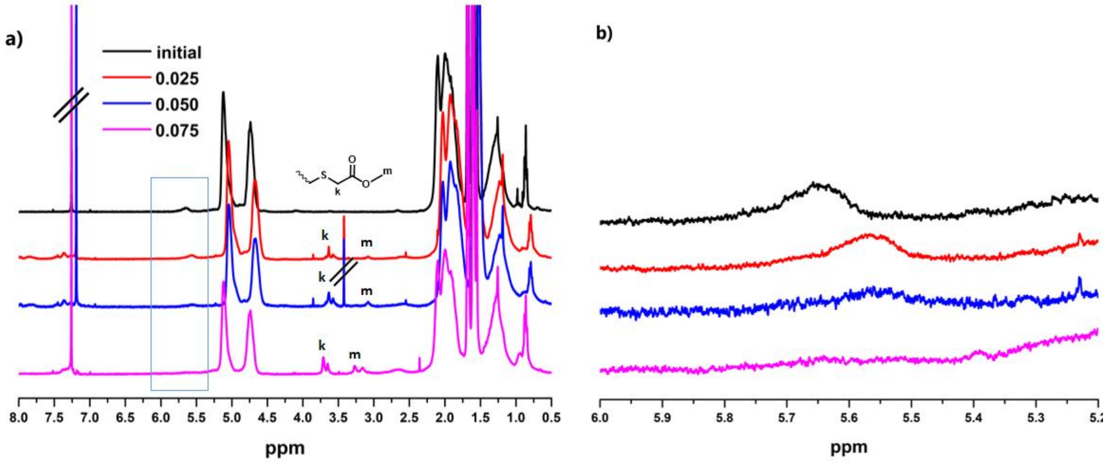

Figure S18. ^{1}H NMR (400 MHz, $CDCl_3$-*d*) spectrum of the 1,2- PMy post-modified with methyl thioglycolate.

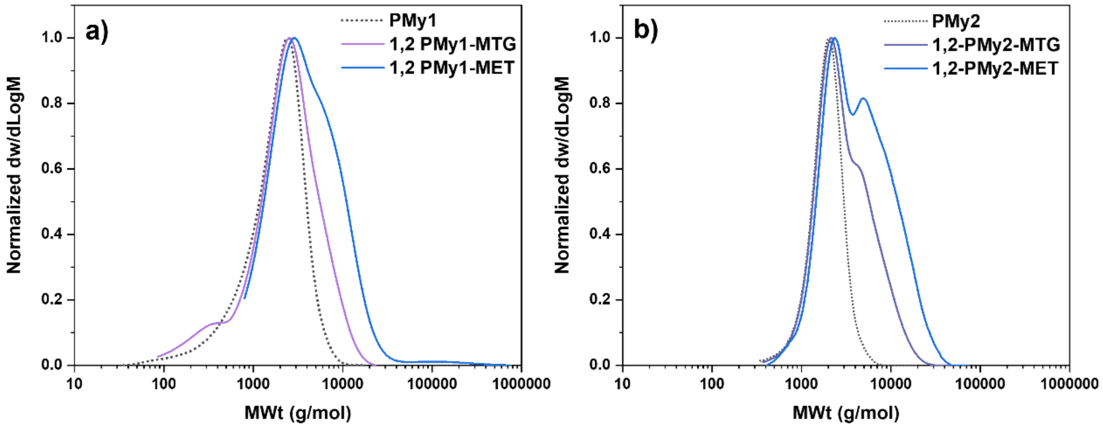

Figure S19. THF-SEC traces of PMy and its thiol-ene derivatives, calibrated with narrow molecular weight PMMA standards.

Table S2. Photofunctionalisation of 1,2-PMy with different types of thiols

Entry	Thiols	[PMy]:[thiol]:[PI][a]	Time (h)	F.D.[b] of 1,2- (%)	F.D. of 1,4- (%)
1	Methyl thiolglycolate (MTG)	1:0.075:0.05	1	100	-
2	2-Mercaptoethanol (MET)	1:0.075:0.05	1	57.1	-

3	MET		24	71.4	-
4	MET	1:0.5:0.05	1	57.1	-
5	MET	1:1:0.05	1	100	4
6	Mercaptopropionic acid (MAC)	1:0.075:0.05	1	46.2	-
7	MAC	1:0.075:0.05	24	57.1	-
8	MAC	1:0.075:0.05	48	100	-
9	MAC	1:0.25:0.05	1	71.4	-
10	MAC	1:0.5:0.05	1	100	10
11	3-Mercapto-1-hexanol (MH)	1:0.075:0.05	1	57.1	-
12	MH	1:0.075:0.05	24	71.4	-
13	MH	1:0.075:0.05	48	100	-
14	MH	1:0.5:0.05	1	71.4	-
15	MH	1:1:0.05	1	100	2
16	1-Thioglycerol (THG)	1:0.075:0.05	1	57.1	-
17	THG	1:0.075:0.05	24	71.4	-
18	THG	1:0.5:0.05	1	86	-
19	THG	1:1:0.05	1	100	0.8-

[a]Photoinitiator (PI)= 2,2-dimethoxy-2-phenylacetophenone (DMPA), reaction conditions determined by moles to per myrcene unit, UV reactor: Analytic Jena (MODEL CL-1000) with λmax = 365 nm; [b] F.D.= Functionalisation degree.

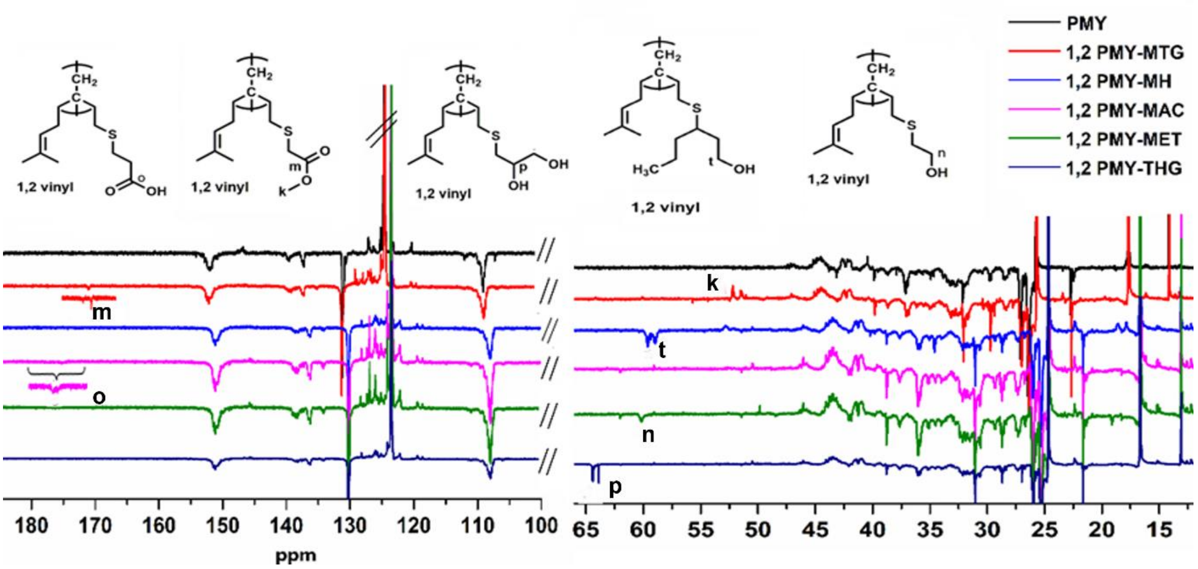

Figure S20. ^{13}C NMR (CDCl$_3$-*d*, 500 MHz) of thiol functionalised 1,2-PMy (**Table 2, entries 1-5**).

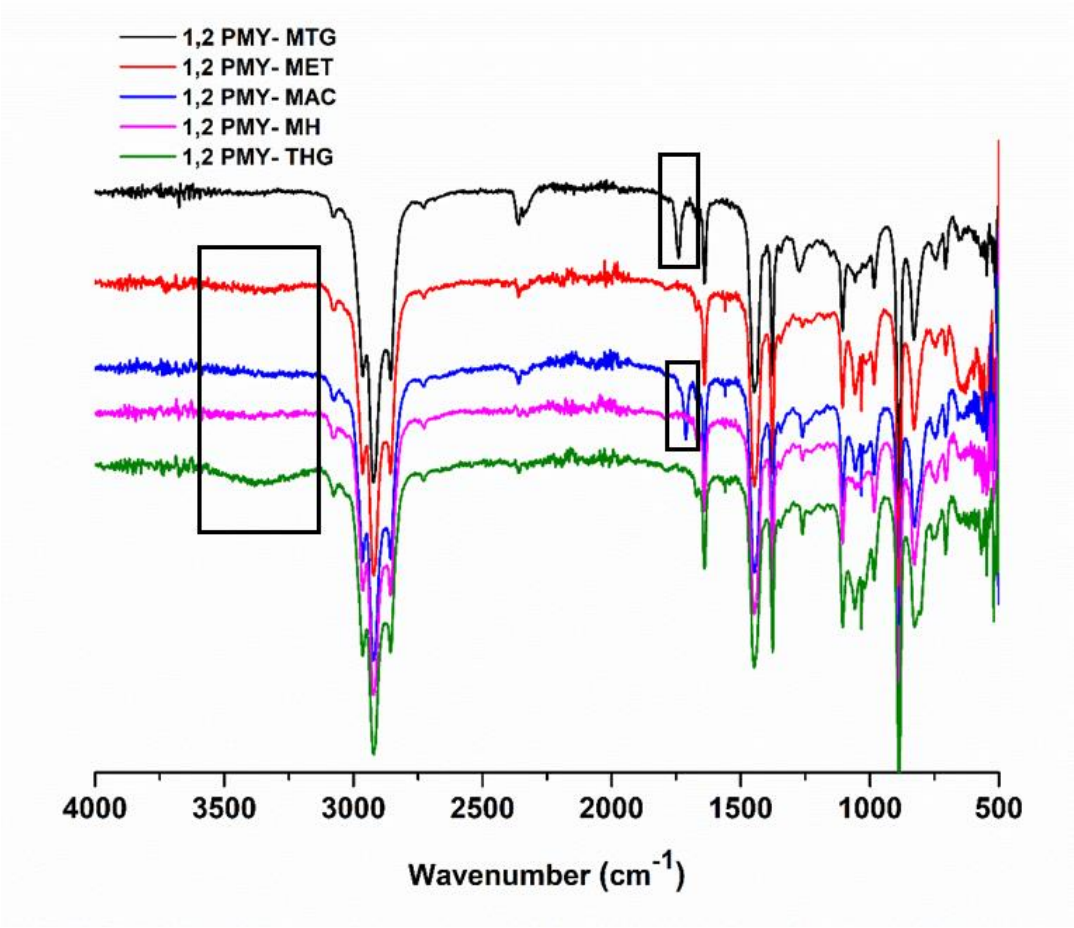

Figure S21. FT-IR spectra of different type of thiols functionalised 1,2-PMy. (Conditions: [PMy]:[thiol]:[DMPA]: = 1:0.075:0.05 in 1h) (**Table S2, entries 1, 2, 6, 11 & 16**).

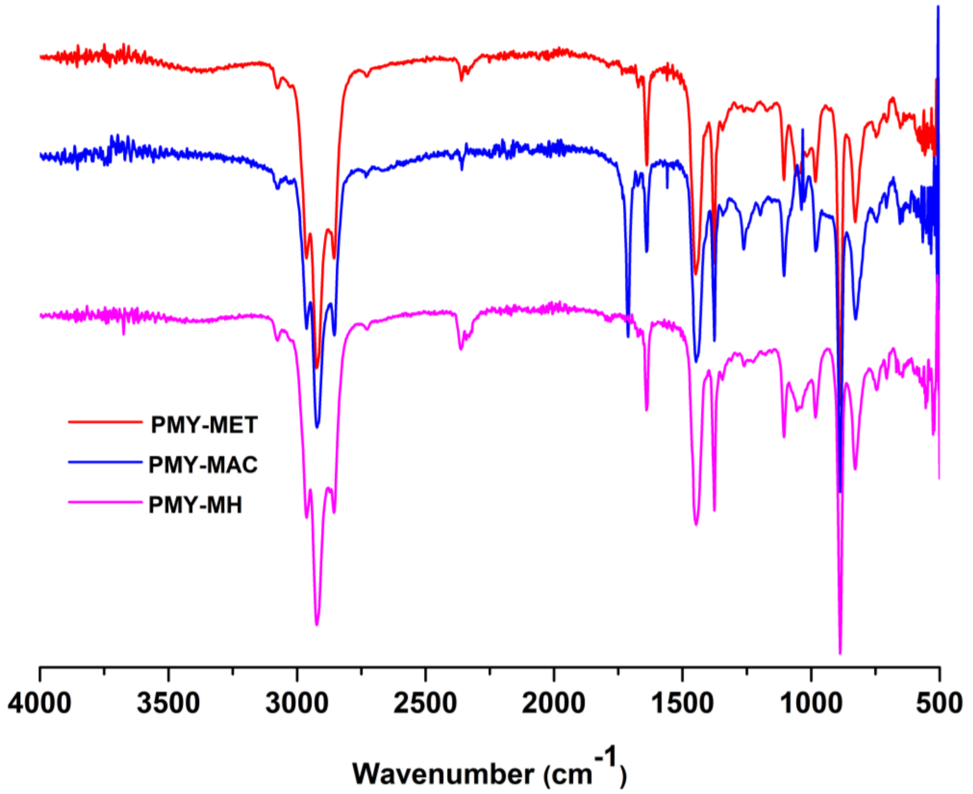

Figure S22. FT-IR spectra of different type of thiols functionalised PMy. (**Table S2**, **entry 5, 10 & 15**)

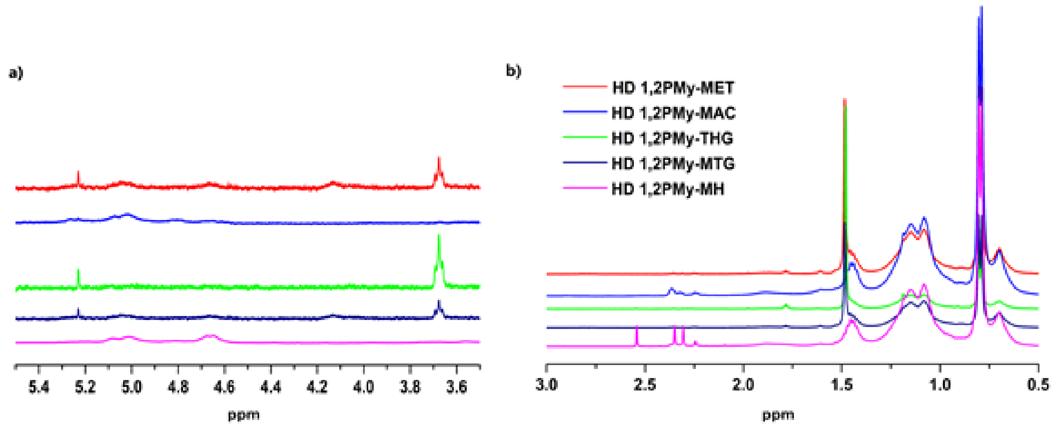

Figure S23. ^{1}H NMR (500 MHz, $CDCl_3$-*d*) of hydrogenated thiol-derivatised 1,2-PMy.

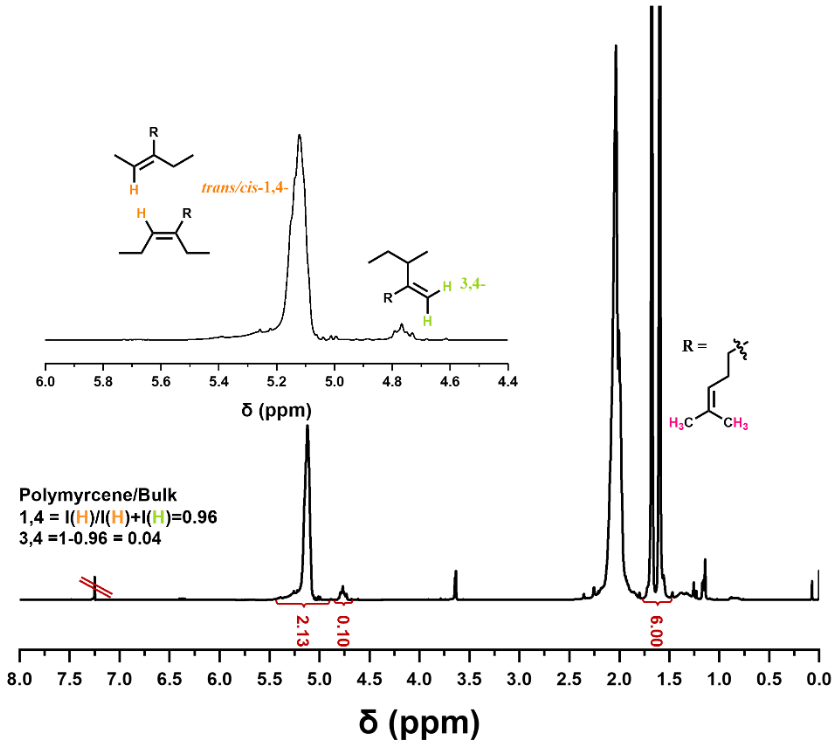

Figure S24. ^{1}H NMR spectrum (500 MHz, $CDCl_3$-*d*) of PMy synthesized *via* free radical polymerisation; determination of stereochemistry (**Table 1, entry 1**).

a)

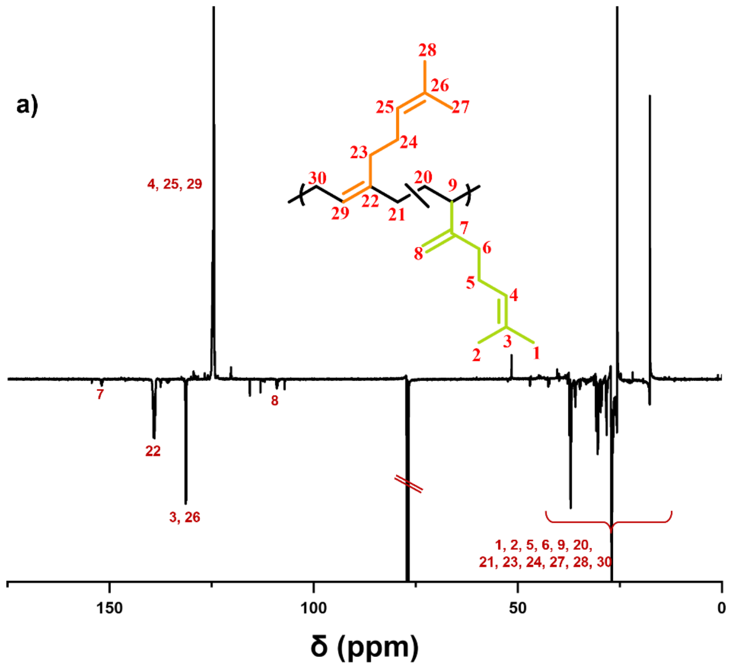
4, 25, 29
7
22
3, 26
8
1, 2, 5, 6, 9, 20,
21, 23, 24, 27, 28, 30
150
100
50
0
δ (ppm)

b)

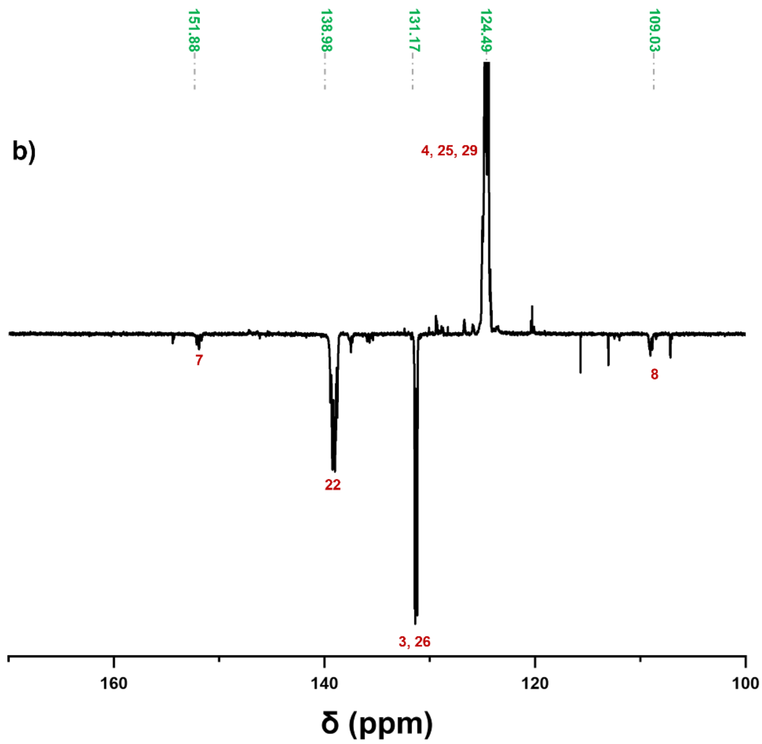
151.88
138.98
131.17
124.49
109.03
4, 25, 29
7
22
3, 26
8
160
140
120
100
δ (ppm)

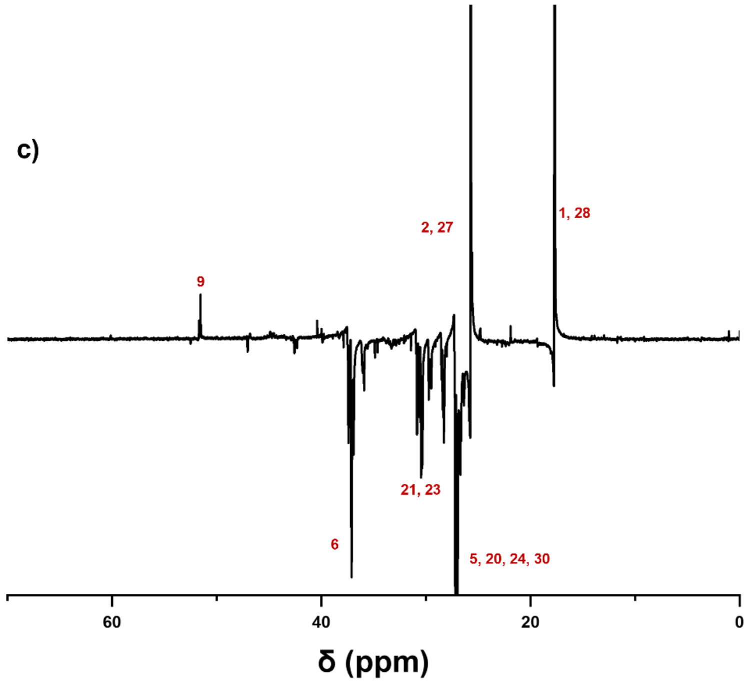

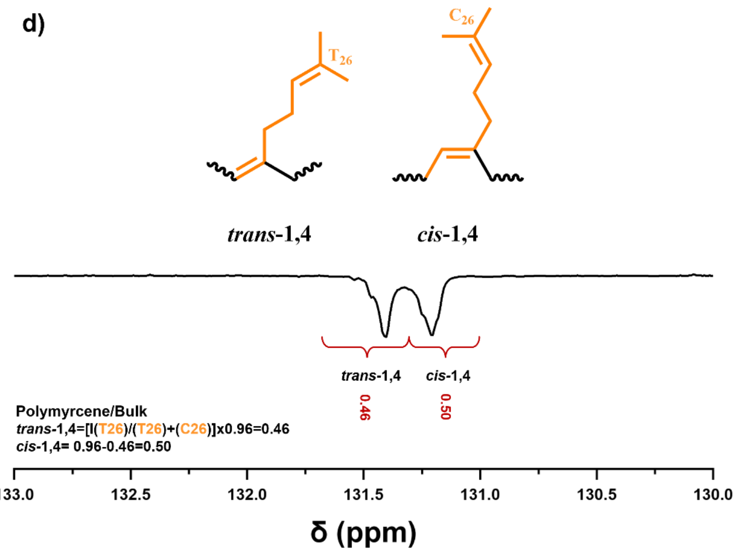

Figure S25. a). ^{13}C NMR spectrum (500 MHz, $CDCl_3$-*d*) of PMy synthesized *via* free radical polymerisation; determination of stereochemistry b) Expanded ^{13}C NMR spectrum of a) in the range of 170-100 ppm c) Expanded ^{13}C NMR spectrum of a) in the range of 70-0 ppm d) Expanded ^{13}C NMR spectrum of a) in the range of 133-130 ppm (**Table 1, entry 1**) .

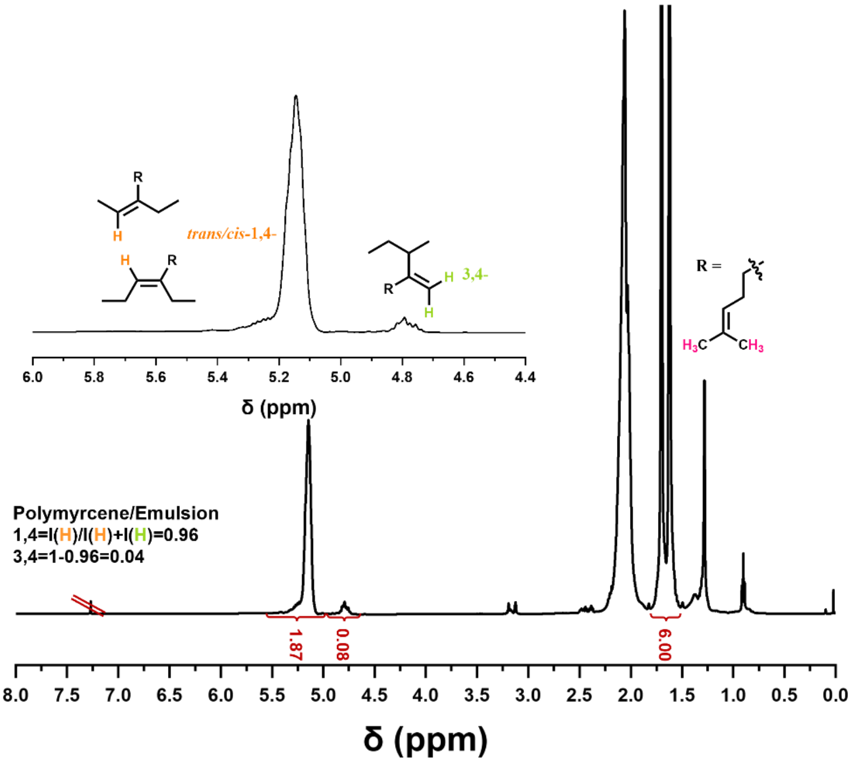

Figure S26. ^{1}H NMR spectra (500 MHz, $CDCl_3$-*d*) of PMy synthesized *via* emulsion polymerisation; determination of stereochemistry (**Table 1, entry 2**).

a)

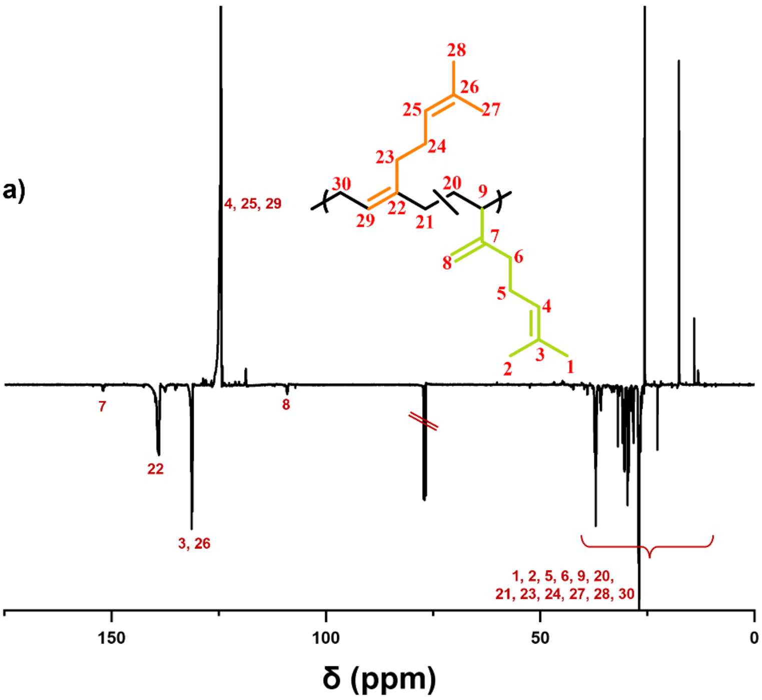
4, 25, 29
7
22
3, 26
8
1, 2, 5, 6, 9, 20,
21, 23, 24, 27, 28, 30
150
100
50
0
δ (ppm)

b)

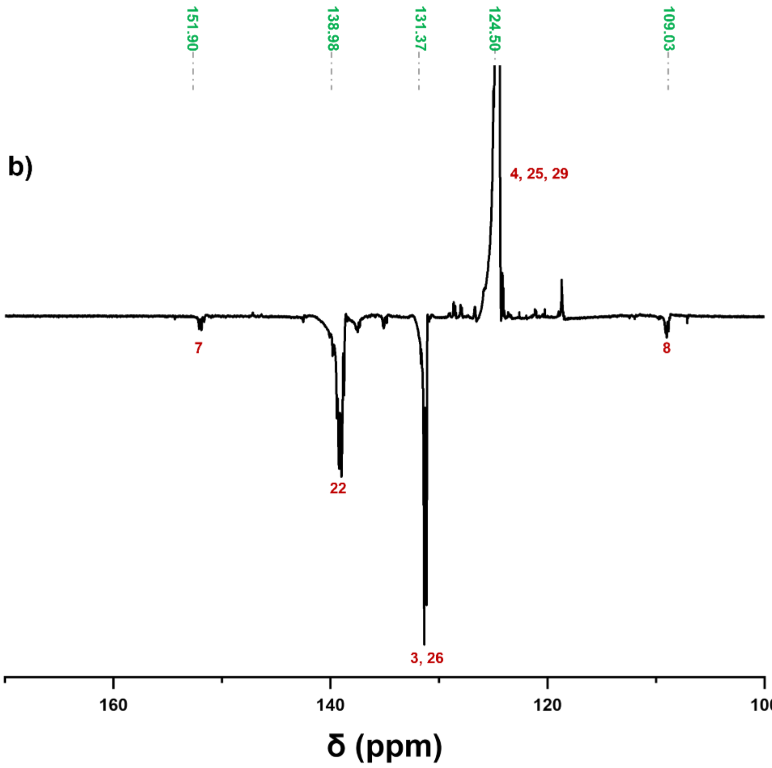
151.90
138.98
131.37
124.50
109.03
4, 25, 29
7
22
3, 26
8
160
140
120
100
δ (ppm)

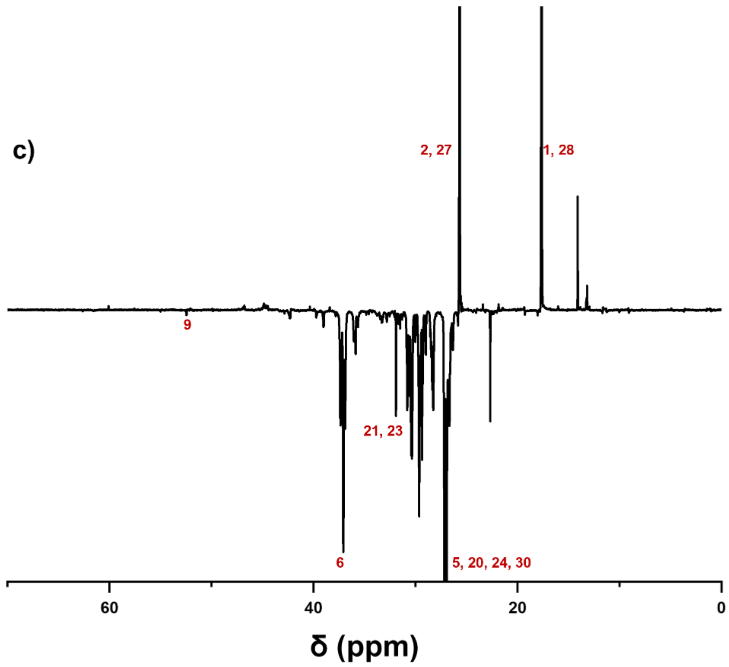

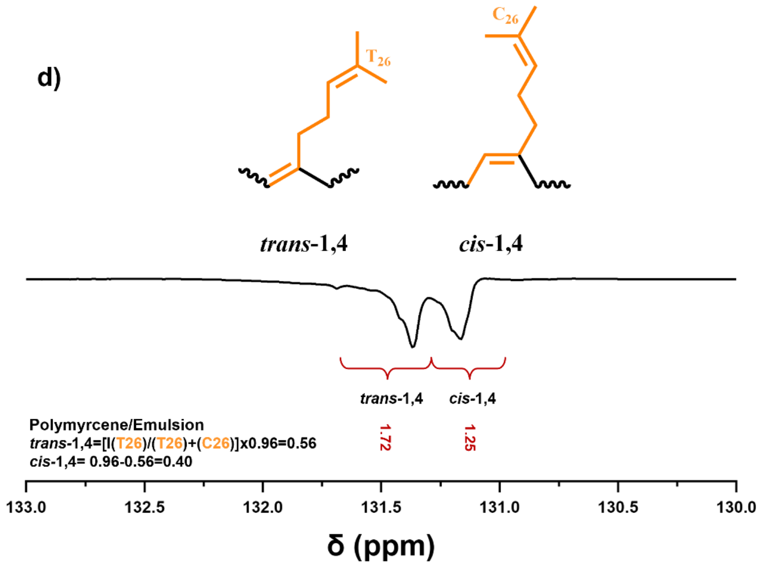

Figure S27. a). ^{13}C NMR spectrum (500 MHz, $CDCl_3$-*d*) of PMy synthesized *via* emulsion polymerisation; determination of stereochemistry b) Expanded ^{13}C NMR spectrum of a) in the range of 170-100 ppm c) Expanded ^{13}C NMR spectrum of a) in the range of 70-0 ppm d) Expanded ^{13}C NMR spectrum of a) in the range of 133-130 ppm (**Table 1, entry 2**).

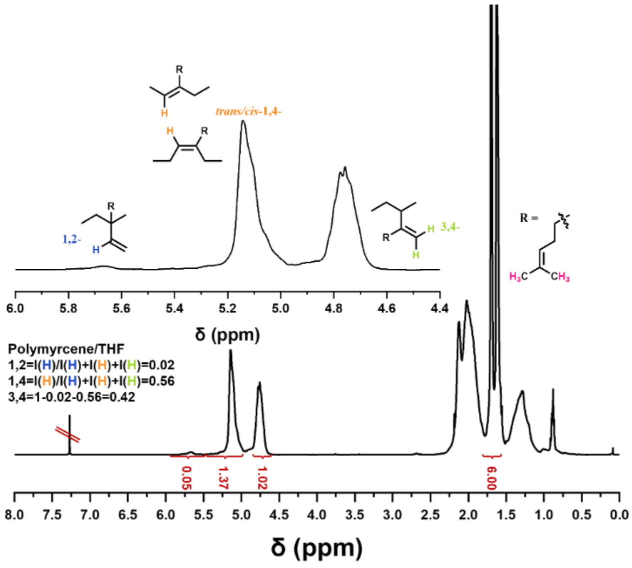

Figure S28. ^{1}H NMR spectra (500 MHz, $CDCl_3$-*d*) of PMy synthesized *via* anionic polymerisation in THF with targeted DP_n = 10, V_{My}:V_{THF} = 1:2; determination of stereochemistry (**Table 1, entry 3**).

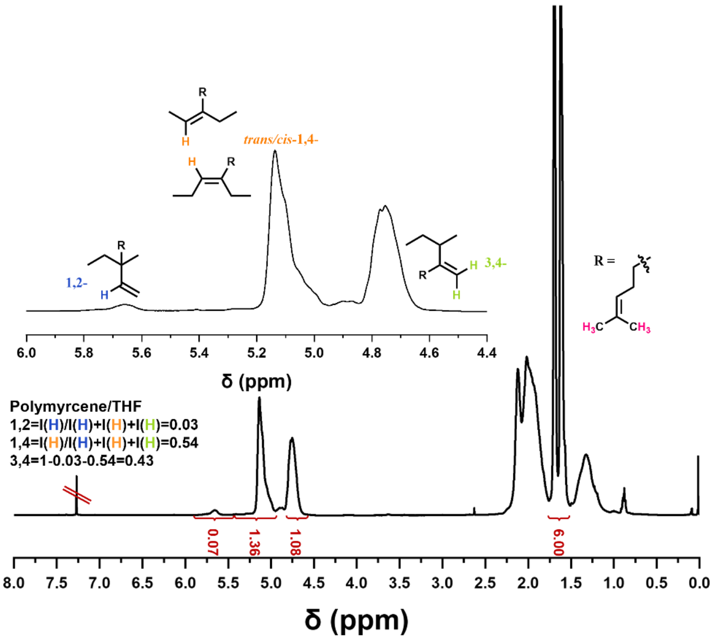

Figure S29. ^{1}H NMR spectra (500 MHz, $CDCl_3$-*d*) of PMy synthesized *via* anionic polymerisation in THF with targeted DP_n = 25, V_{My}:V_{THF} = 1:2; determination of stereochemistry(**Table 1, entry 4**) .

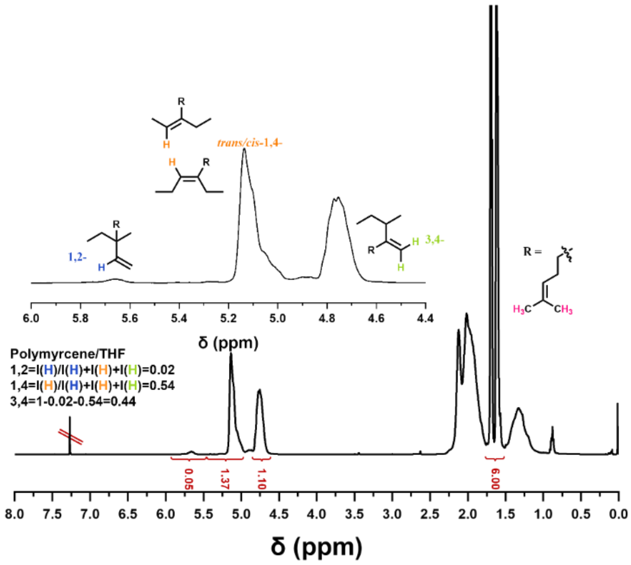

Figure S30. ^{1}H NMR spectra (500 MHz, $CDCl_3$-*d*) of PMy synthesized *via* anionic polymerisation in THF with targeted DP_n = 25, V_{My}:V_{THF} = 2:1; determination of stereochemistry (**Table 1, entry 5**).

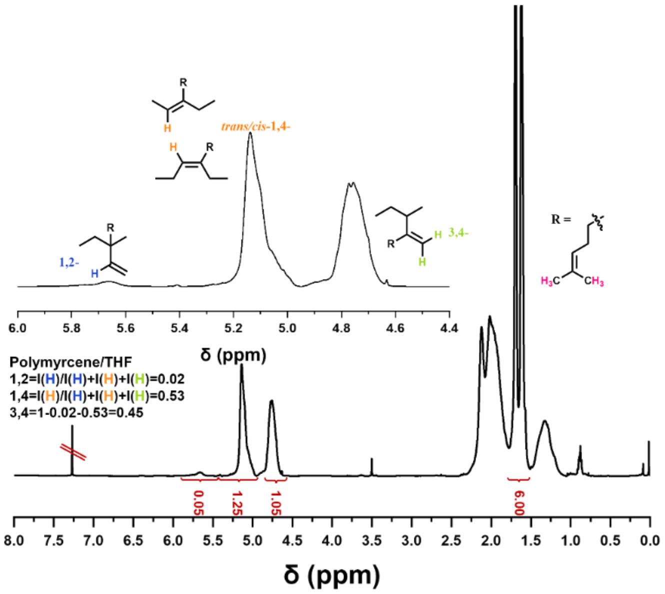

Figure S31. ^{1}H NMR spectra (500 MHz, $CDCl_3$-*d*) of PMy synthesized *via* anionic polymerisation in THF with targeted DP_n = 25, V_{My}:V_{THF} = 1:1; determination of stereochemistry (**Table 1, entry 6**).

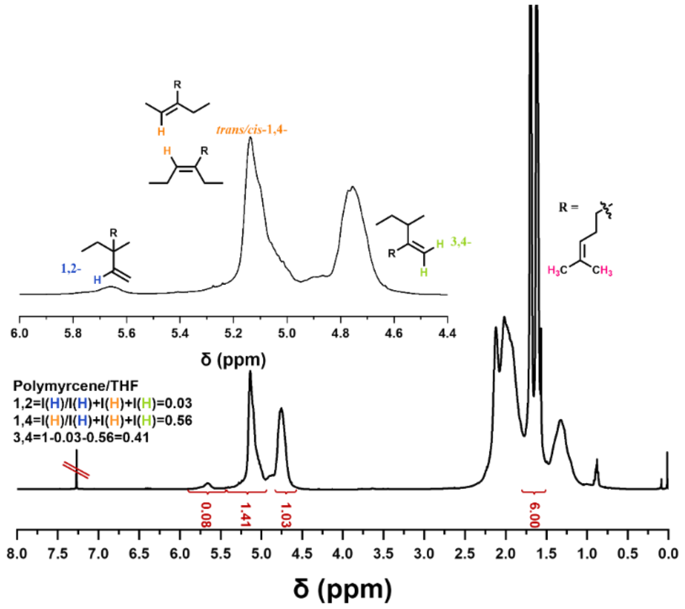

Figure S32. ^{1}H NMR spectra (500 MHz, $CDCl_3$-*d*) of PMy synthesized *via* anionic polymerisation in THF with targeted DP_n = 25, V_{My}:V_{THF} = 1:3; determination of stereochemistry (**Table 1, entry 7**).

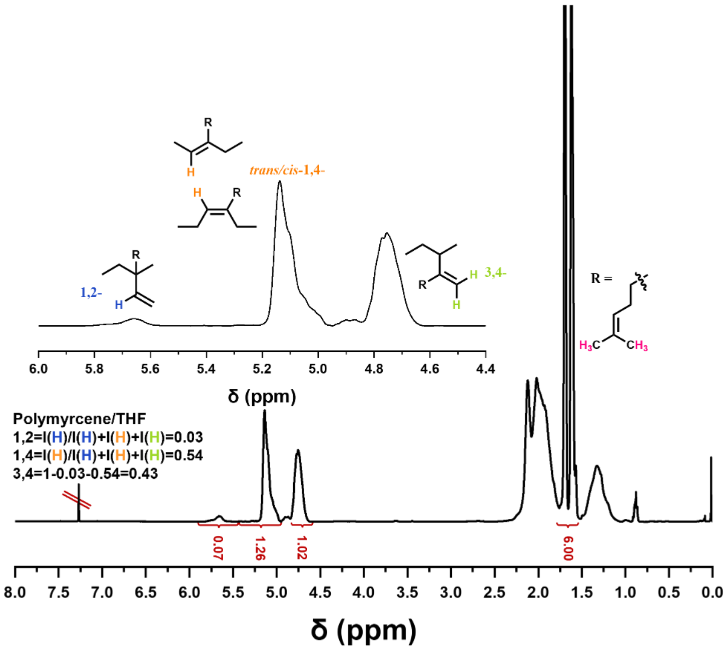

Figure S33. ^{1}H NMR spectra (500 MHz, $CDCl_3$-*d*) of PMy synthesized *via* anionic polymerisation in THF with targeted DP_n = 25, V_{My}:V_{THF} = 1:4; determination of stereochemistry (**Table 1, entry 8**).

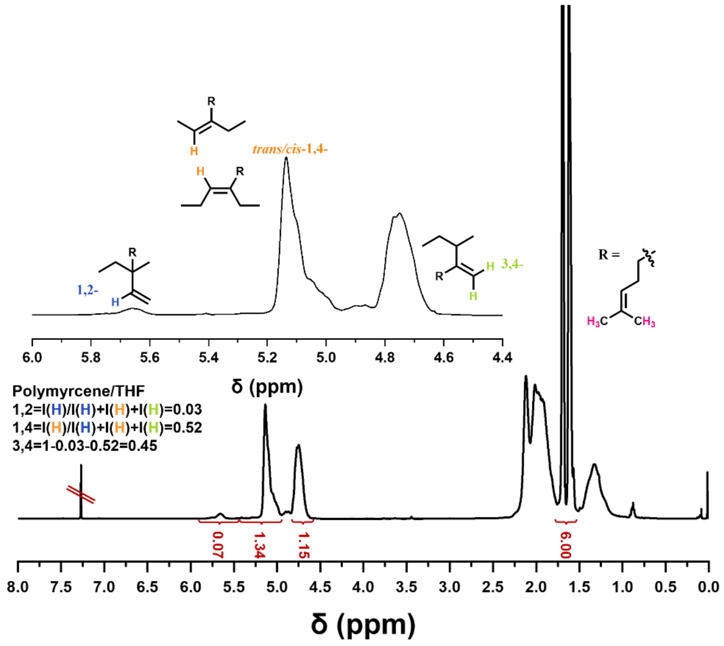

Figure S34. ^{1}H NMR spectra (500 MHz, $CDCl_3$-*d*) of PMy synthesized *via* anionic polymerisation in THF with targeted DP_n = 50, V_{My}:V_{THF} = 1:2; determination of stereochemistry(**Table 1, entry 9**) .

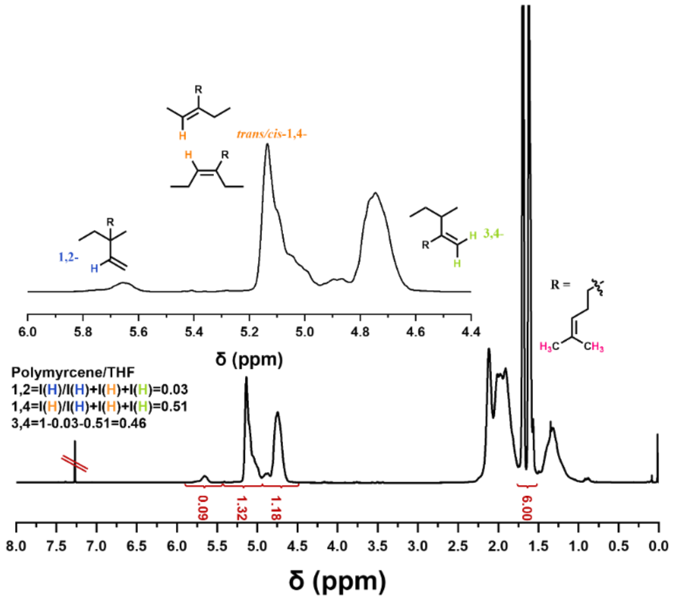

Figure S35. ^{1}H NMR spectra (500 MHz, $CDCl_3$-*d*) of PMy synthesized *via* anionic polymerisation in THF with targeted DP_n = 200, V_{My}:V_{THF} = 1:2; determination of stereochemistry (**Table 1, entry 11**).

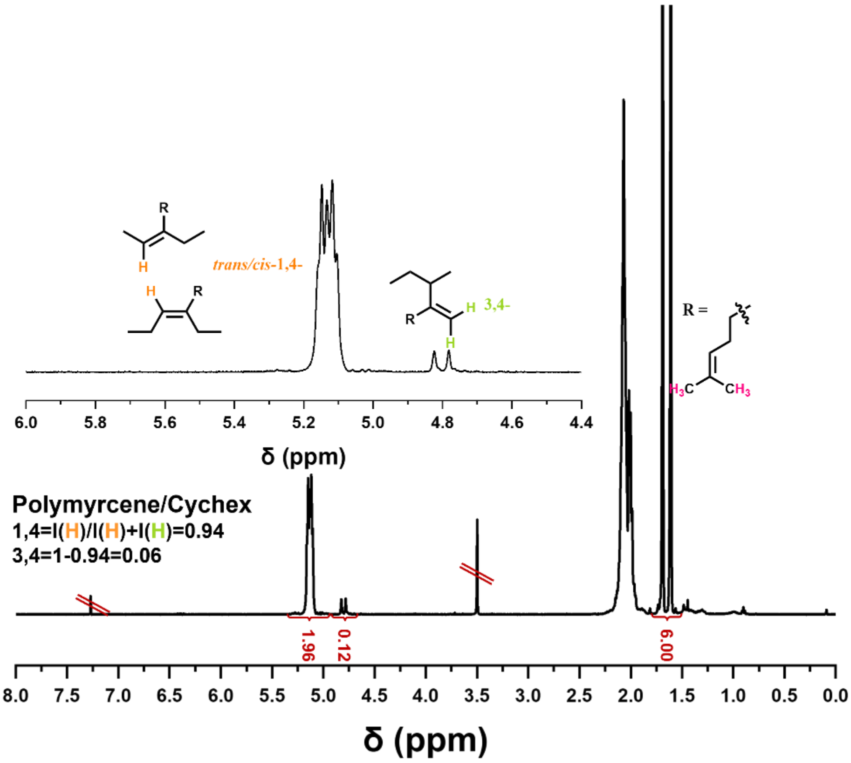

Figure S36. ^{1}H NMR spectra (500 MHz, $CDCl_3$-*d*) of PMy synthesized *via* anionic polymerisation in cyclohexane with targeted DP_n = 100, V_{My}:V_{THF} = 1:2; determination of stereochemistry (**Table 1, entry 12**).

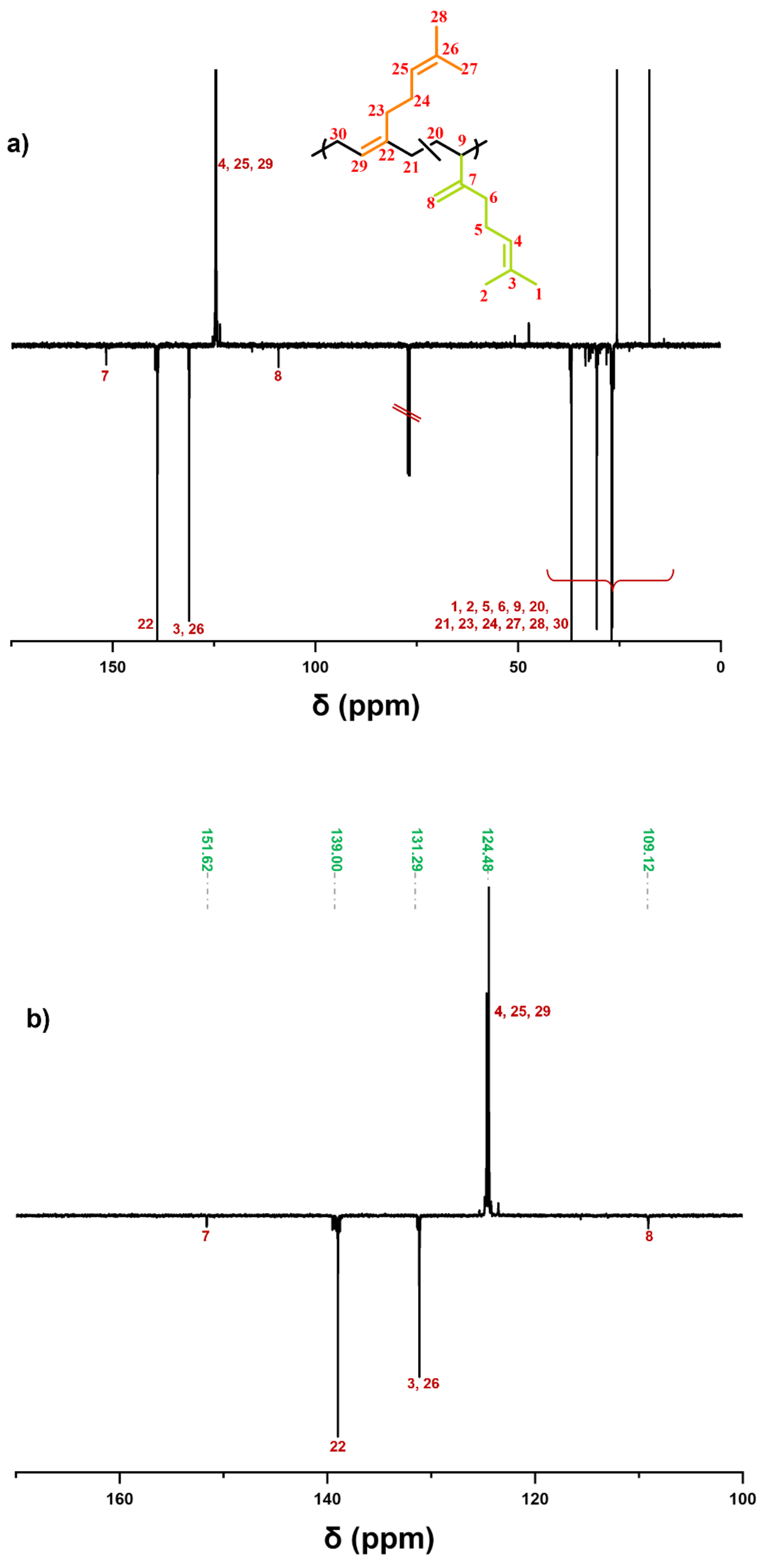
a)
4, 25, 29
7
8
22
3, 26
1, 2, 5, 6, 9, 20,
21, 23, 24, 27, 28, 30
150
100
50
0
δ (ppm)
b)
151.62
139.00
131.29
124.48
109.12
4, 25, 29
7
8
3, 26
22
160
140
120
100
δ (ppm)

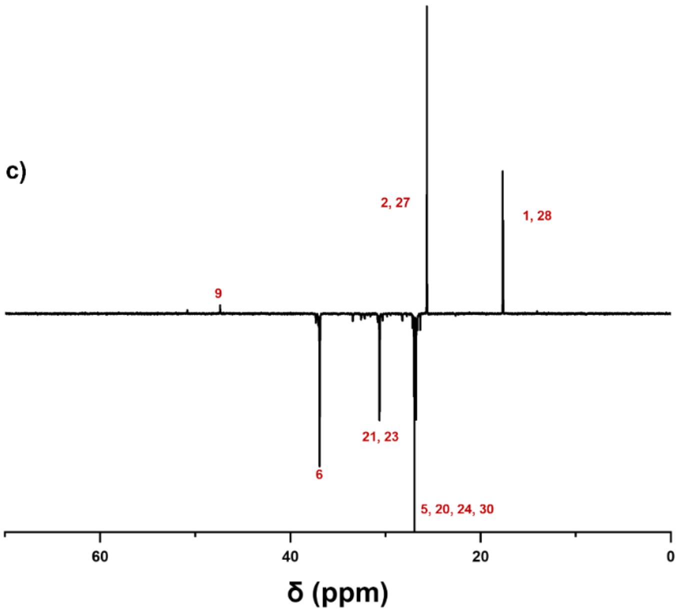

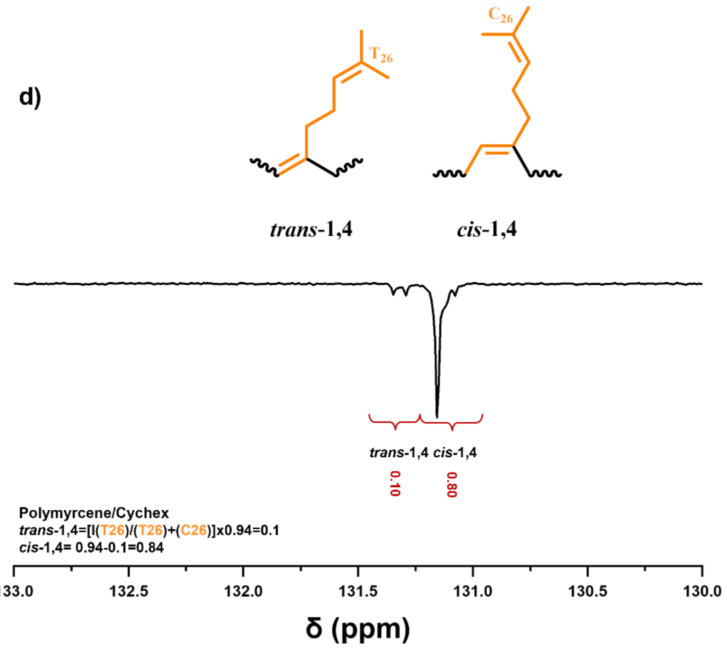

Figure S37. a). ^{13}C NMR spectrum (500 MHz, $CDCl_3$-*d*) of PMy synthesized *via* anionic polymerisation in cyclohexane with targeted DP_n = 100, V_{My}:V_{THF} = 1:2; determination of stereochemistry b) Expanded ^{13}C NMR spectrum of a) in the range of 170-100 ppm c) Expanded ^{13}C NMR spectrum of a) in the range of 70-0 ppm d) Expanded ^{13}C NMR spectrum of a) in the range of 133-130 ppm (**Table 1, entry 12**).

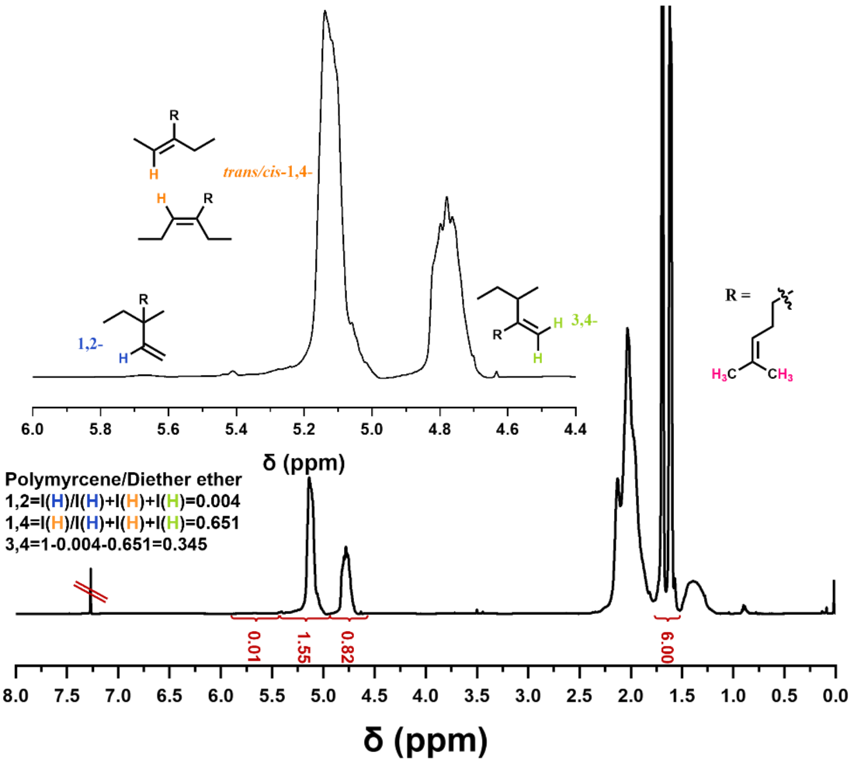

Figure S38. ^{1}H NMR spectra (500 MHz, $CDCl_3$-*d*) of PMy synthesized *via* anionic polymerisation in diethyl ether with targeted DP_n = 100, V_{My}:V_{THF} = 1:2; determination of stereochemistry (**Table 1, entry 13**).

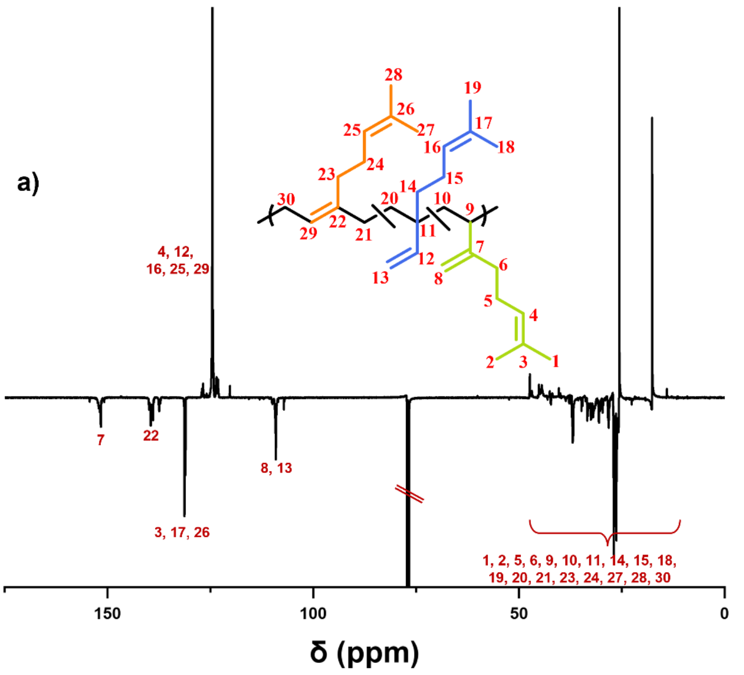
a)
4, 12, 16, 25, 29
7
22
3, 17, 26
8, 13
1, 2, 5, 6, 9, 10, 11, 14, 15, 18, 19, 20, 21, 23, 24, 27, 28, 30
150
100
50
0
δ (ppm)

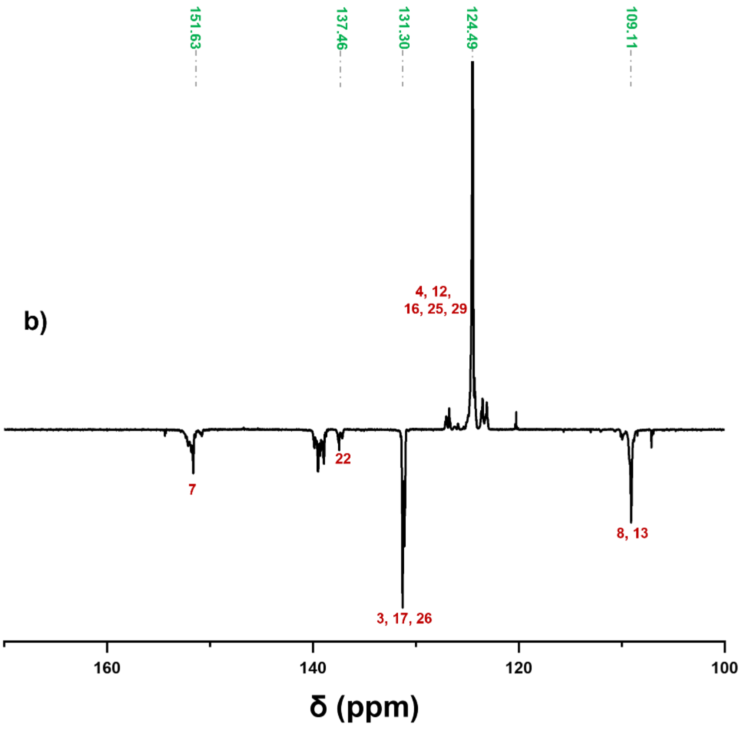
b)
151.63
137.46
131.30
124.49
109.11
4, 12, 16, 25, 29
7
22
3, 17, 26
8, 13
160
140
120
100
δ (ppm)

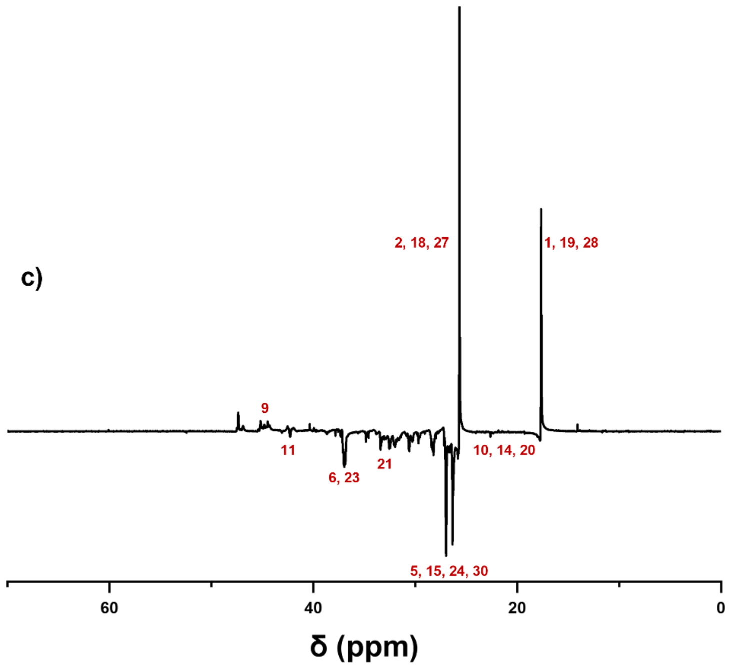

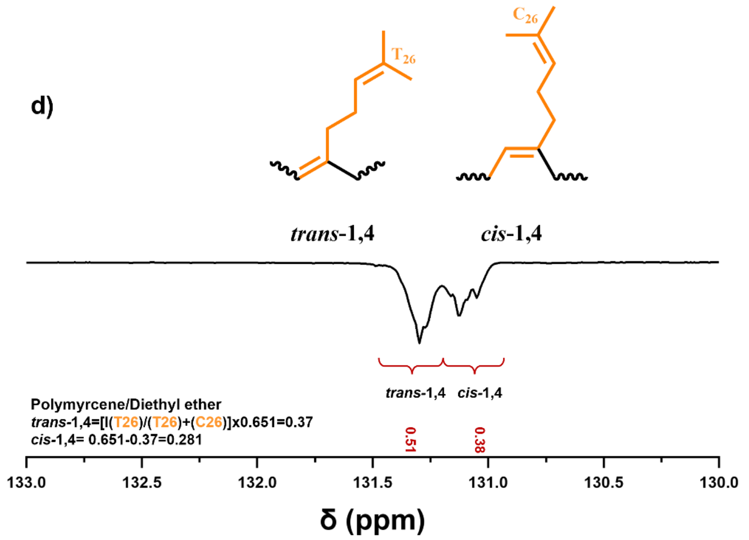

Figure S39. A). ^{13}C NMR spectrum (500 MHz, $CDCl_3$-*d*) of Pmy synthesized *via* anionic polymerisation in diethyl ether with targeted DP_n = 100, V_{My}:V_{THF} = 1:2; determination of stereochemistry. B) Expanded ^{13}C NMR spectrum of a) in the range of 170-100 ppm c) Expanded ^{13}C NMR spectrum of a) in the range of 70-0 ppm d) Expanded ^{13}C NMR spectrum of a) in the range of 133-130 ppm (**Table 1, entry 13**).

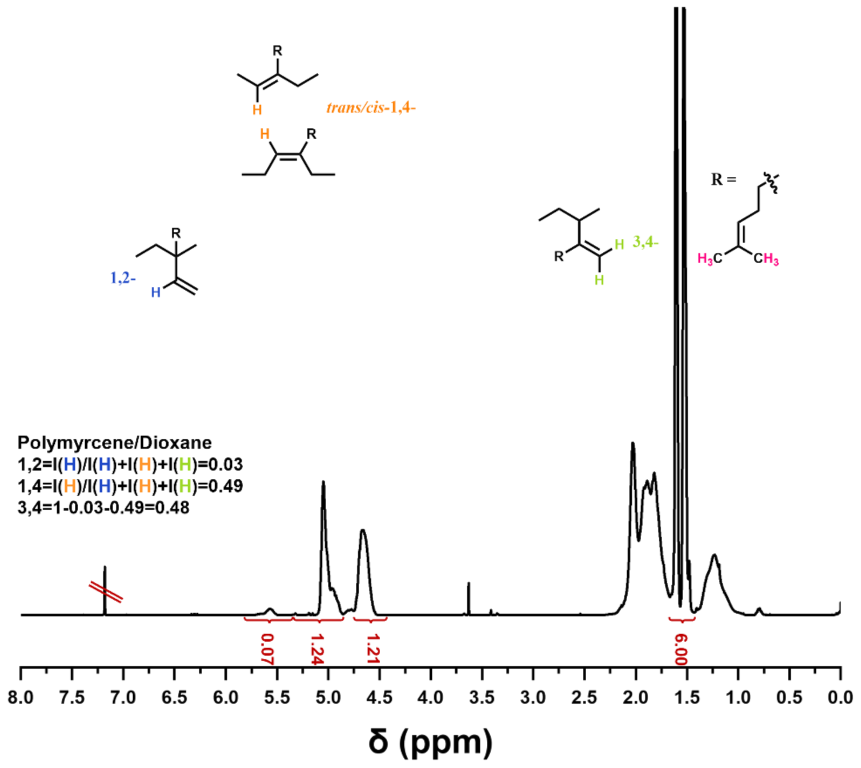

Figure S40. ^{1}H NMR spectra (500 MHz, $CDCl_3$-*d*) of PMy synthesized *via* anionic polymerisation in dioxane with targeted DP_n = 100, V_{My}:V_{THF} = 1:2; determination of stereochemistry (**Table 1, entry 14**).

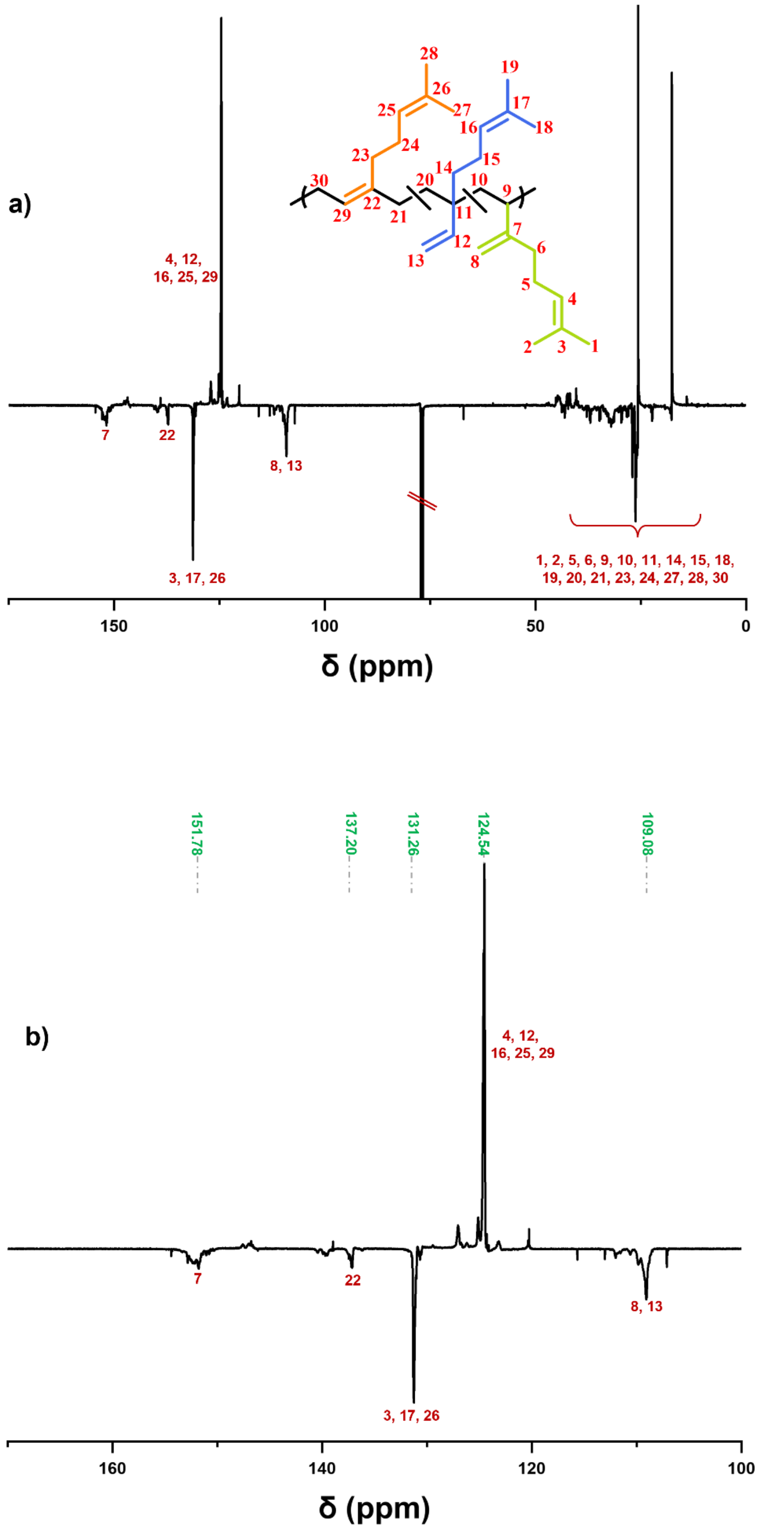

a)
4, 12,
16, 25, 29
7
22
8, 13
3, 17, 26
1, 2, 5, 6, 9, 10, 11, 14, 15, 18,
19, 20, 21, 23, 24, 27, 28, 30
150
100
50
0
δ (ppm)
b)
151.78
137.20
131.26
124.54
109.08
4, 12,
16, 25, 29
7
22
8, 13
3, 17, 26
160
140
120
100
δ (ppm)

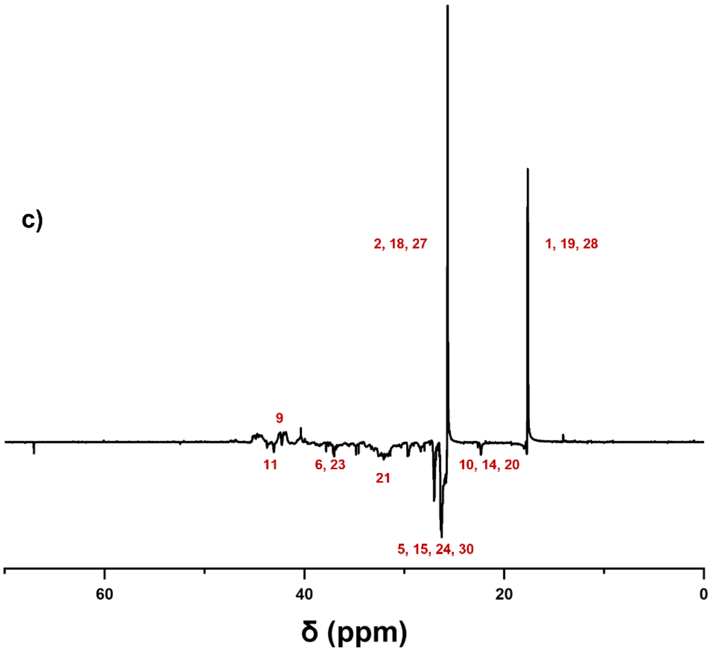

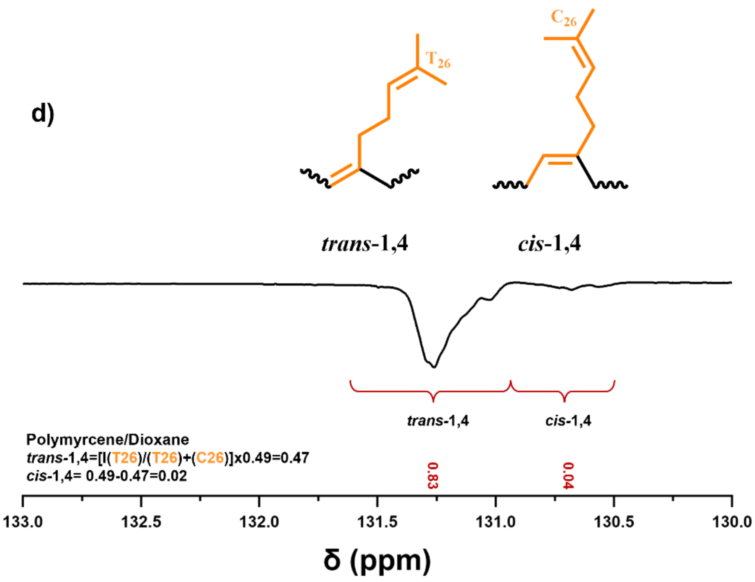

Figure S41. a). ^{13}C NMR spectrum (500 MHz, $CDCl_3$-*d*) of PMy synthesized *via* anionic polymerisation in dioxane with targeted DP_n = 100, V_{My}:V_{THF} = 1:2; determination of stereochemistry. B) Expanded ^{13}C NMR spectrum of a) in the range of 170-100 ppm c) Expanded ^{13}C NMR spectrum of a) in the range of 70-0 ppm d) Expanded ^{13}C NMR spectrum of a) in the range of 133-130 ppm (**Table 1, entry 14**).

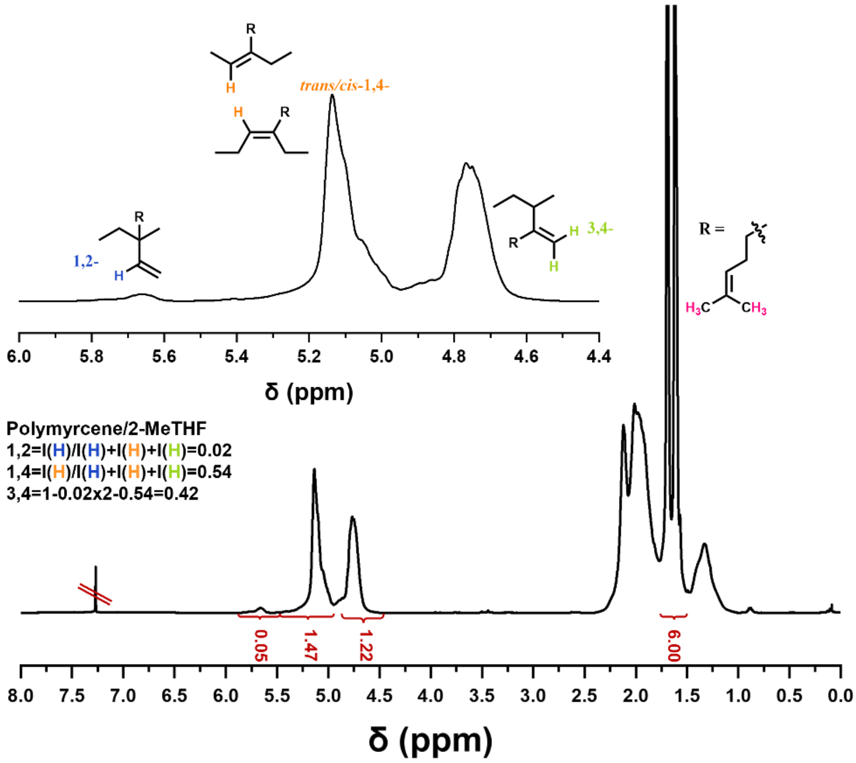

Figure S42. ^{1}H NMR spectra (500 MHz, $CDCl_3$-*d*) of PMy synthesized *via* anionic polymerisation in 2-methyltetrahydrofuran (2-MeTHF) with targeted DP_n = 100, V_{My}:V_{THF} = 1:2; determination of stereochemistry (**Table 1, entry 15**).

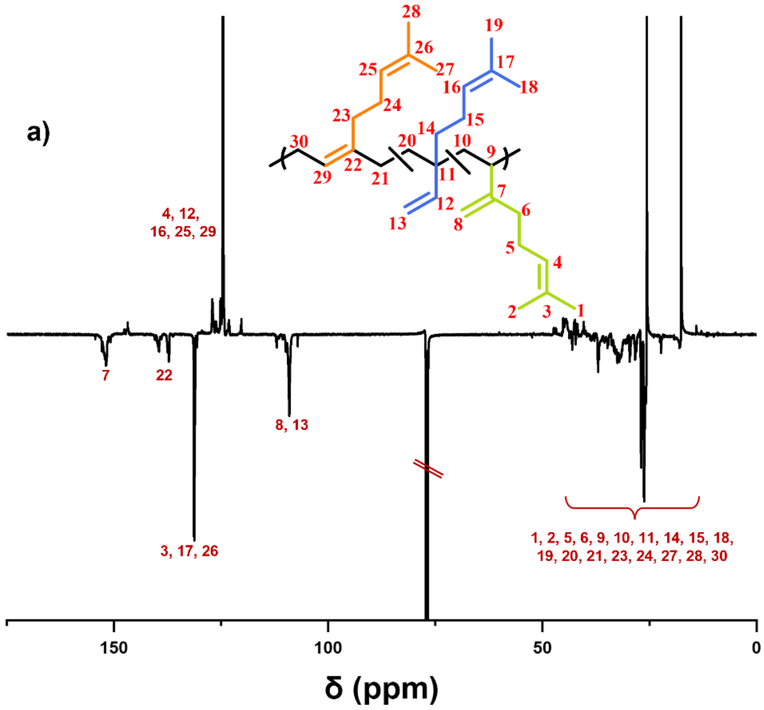
a)
4, 12,
16, 25, 29
7
22
8, 13
3, 17, 26
1, 2, 5, 6, 9, 10, 11, 14, 15, 18,
19, 20, 21, 23, 24, 27, 28, 30
150
100
50
0
δ (ppm)

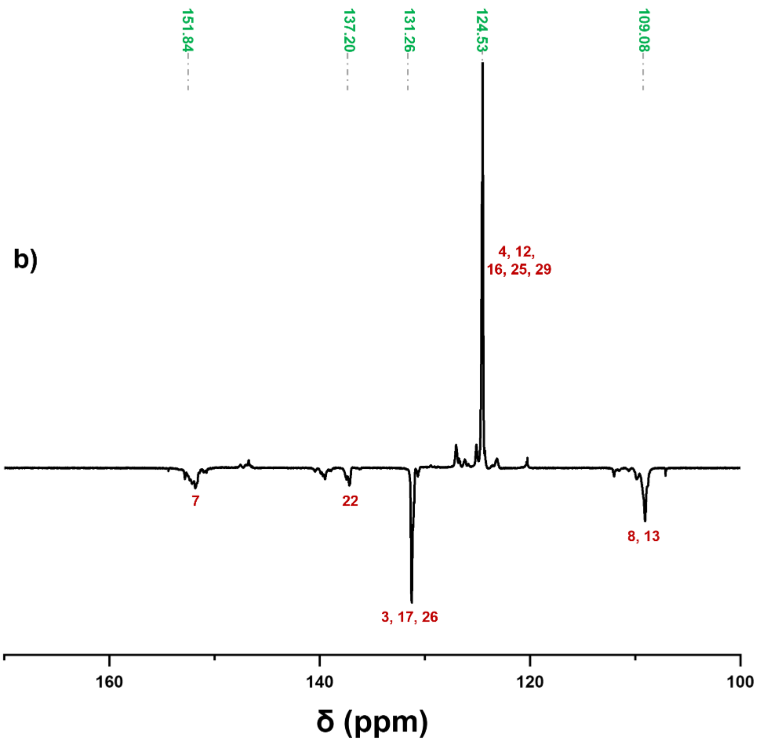
b)
151.84
137.20
131.26
124.53
109.08
4, 12,
16, 25, 29
7
22
8, 13
3, 17, 26
160
140
120
100
δ (ppm)

c)

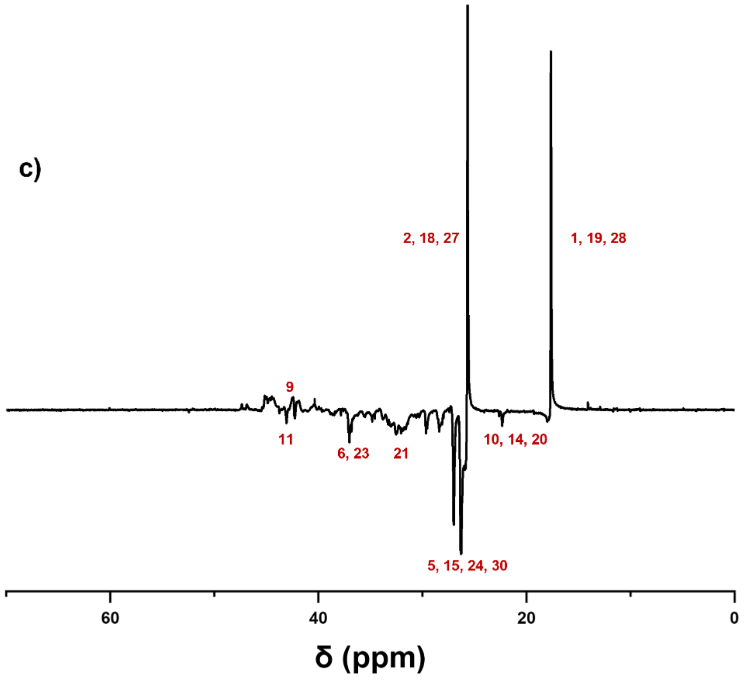

d)

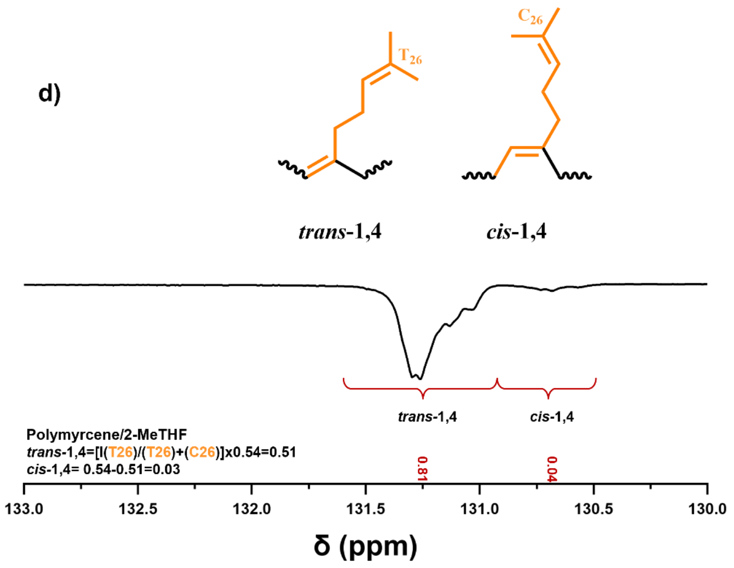

Figure S43. a). ^{13}C NMR spectrum (500 MHz, $CDCl_3$-*d*) of PMy synthesized *via* anionic polymerisation in 2-methyltetrahydrofuran (2-MeTHF) with targeted DP_n=100, V_{My}:V_{THF} = 1:2; determination of stereochemistry b) Expanded ^{13}C NMR spectrum of a) in the range of 170-100 ppm c) Expanded ^{13}C NMR spectrum of a) in the range of 70-0 ppm d) Expanded ^{13}C NMR spectrum of a) in the range of 133-130 ppm (**Table 1, entry 15**).

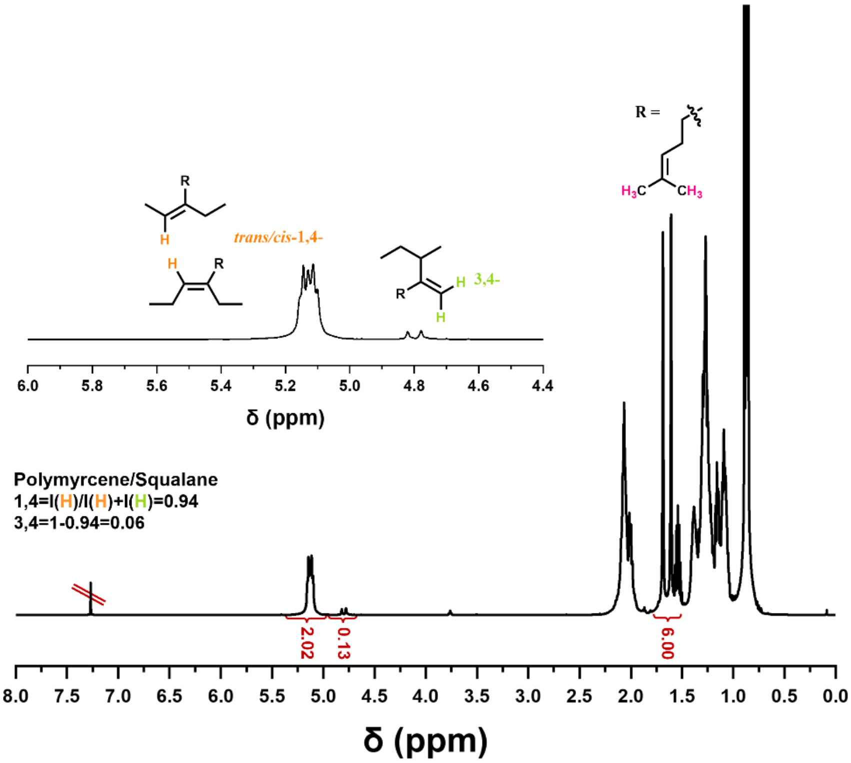

Figure S44. ^{1}H NMR spectra (500 MHz, $CDCl_3$-*d*) of PMy synthesized *via* anionic polymerisation in squalane with targeted DP_n = 100, V_{My}:V_{THF} = 1:2; determination of stereochemistry (**Table 1, entry 16**).

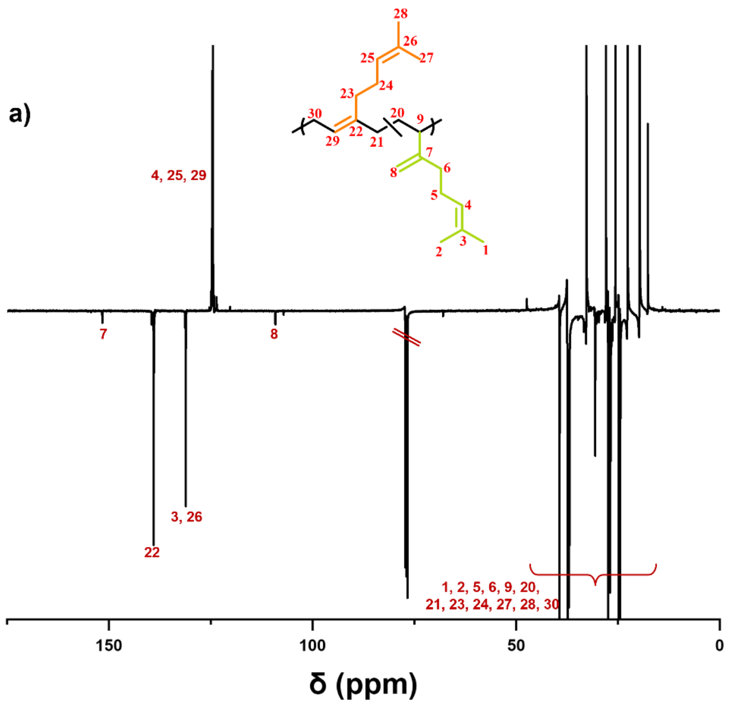
a)
4, 25, 29
7
8
22
3, 26
1, 2, 5, 6, 9, 20,
21, 23, 24, 27, 28, 30
150
100
50
0
δ (ppm)

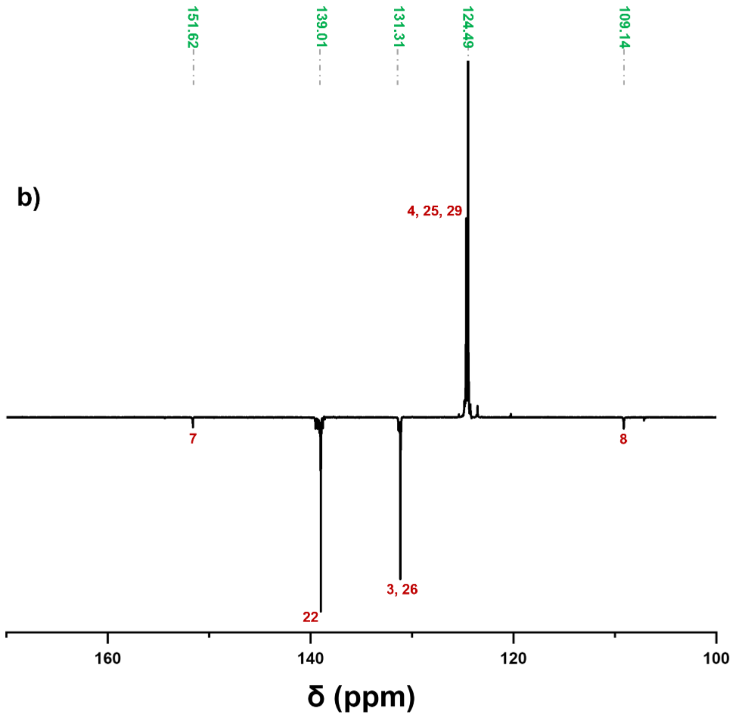
151.62
139.01
131.31
124.49
109.14
b)
4, 25, 29
7
8
22
3, 26
160
140
120
100
δ (ppm)

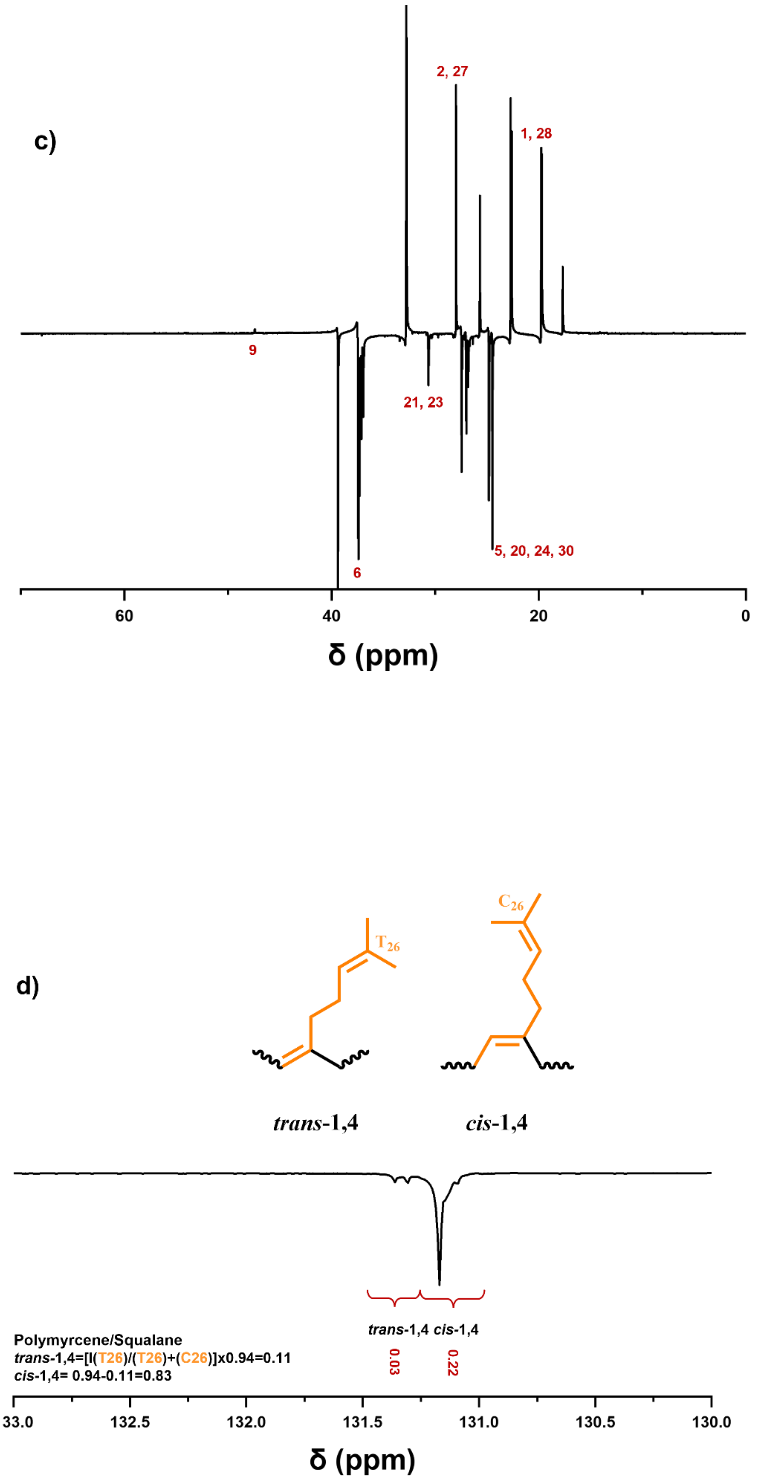

Figure S45. a). ^{13}C NMR spectrum (500 MHz, $CDCl_3$-*d*) of PMy synthesized *via* anionic polymerisation in squalane with targeted DP_n = 100, V_{My}:V_{THF} = 1:2; determination of stereochemistry b) Expanded ^{13}C NMR spectrum of a) in the range of 170-100 ppm c) Expanded ^{13}C NMR spectrum of a) in the range of 70-0 ppm d) Expanded ^{13}C NMR spectrum of a) in the range of 133-130 ppm (**Table 1, entry 16**).

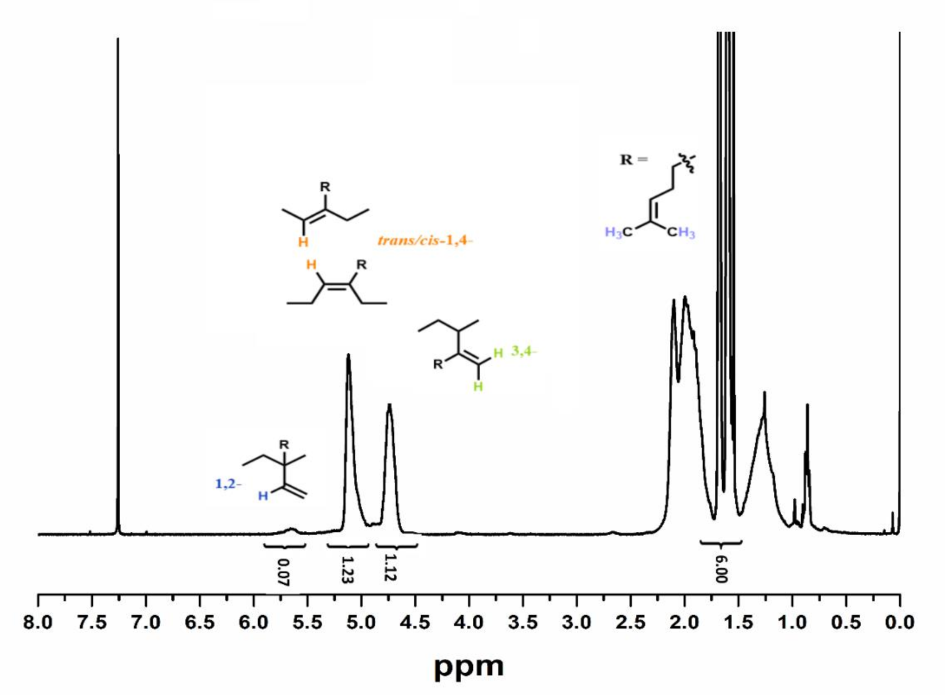

Figure S46. ^{1}H NMR spectrum (500 MHz, $CDCl_3$-*d*) of PMy synthesized *via* anionic polymerisation in THF (**Table 1, entry 3**).

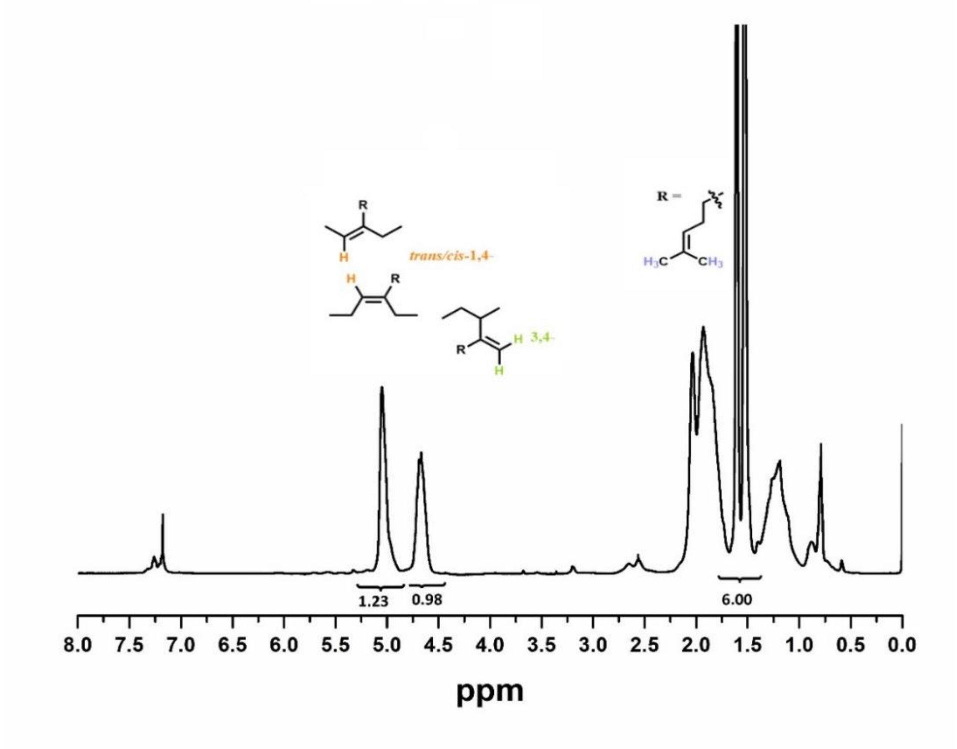

Figure S47. ^{1}H NMR spectrum (500 MHz, $CDCl_3$-*d*) of 3-mercaptopropionic acid functionalised PMy. Conditions: [PMy]:[MAC]:[DMPA]: = 1:0.075:0.05, reaction time 48h. F.D of 1,2-PMy >99%, F.D of 1,4-PMy = 0%, F.D of 3,4-PMy = (1.12-0.07-0.98)/(1.12-0.07) = 6% (**Table S2, entry 6**).

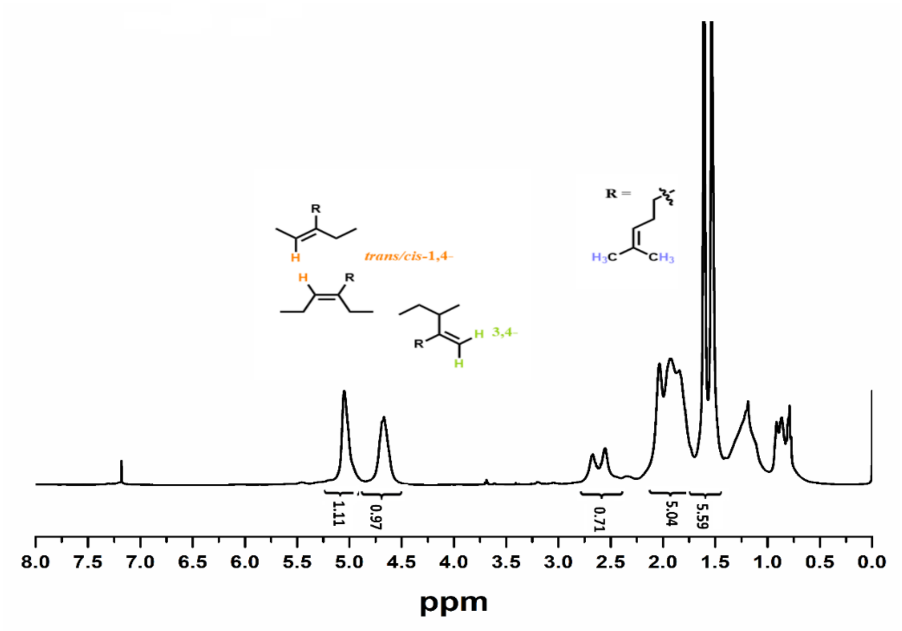

Figure S48. ^{1}H spectrum (500 MHz, $CDCl_3$-*d*) of 3-mercaptopropionic acid functionalised PMy. Conditions: [PMy]:[MAC]:[DMPA]:1:0.5:0.05, reaction time: 1h. F.D of 1,2-PMy >99%, F.D of 1,4-PMy = 9.7%, F.D of 3,4-PMy = (1.12-0.07-0.97)/(1.12-0.07) = 8% (**Table S2 entry 10**).

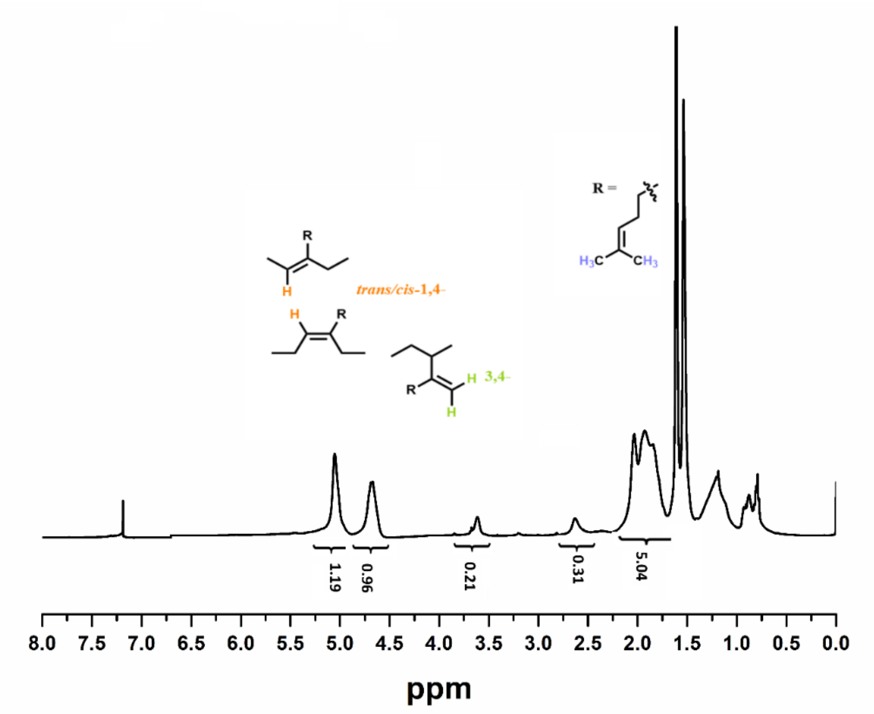

Figure S49. ^{1}H spectrum (500 MHz, $CDCl_3$-*d*) of 2-mercaptoethanol functionalised PMy. Conditions: [PMy]:[MET]:[DMPA]: = 1:1:0.05, reaction time: 1h. F.D of 1,2-PMy >99%, F.D of 1,4-PMy = 3.2%, F.D of 3,4-PMy = (1.12-0.07-0.96)/(1.12-0.07) = 9% (**Table S2, entry 5**).

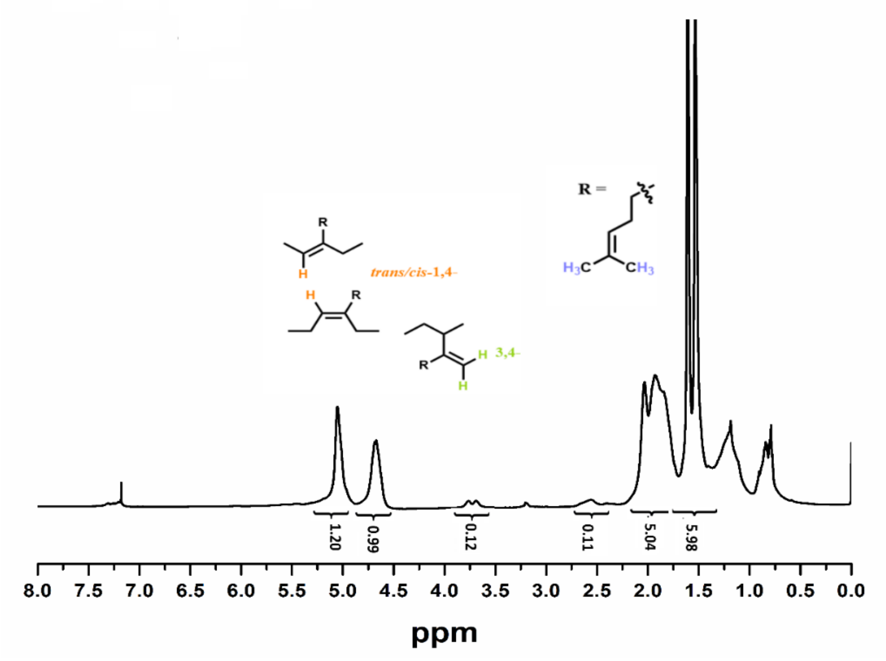

Figure S50. ^{1}H spectrum (500 MHz, $CDCl_3$-*d*) of 3-mercapto-1-hexanol functionalised PMy. Conditions: [PMy]:[MH]:[DMPA]: = 1:1:0.05, reaction time 1h F.D of 1,2-PMy >99%, F.D of 1,4-PMy = 2.4%, F.D of 3,4-PMy = (1.12-0.07-0.99)/(1.12-0.07) = 6% (**Table S2, entry 15**).

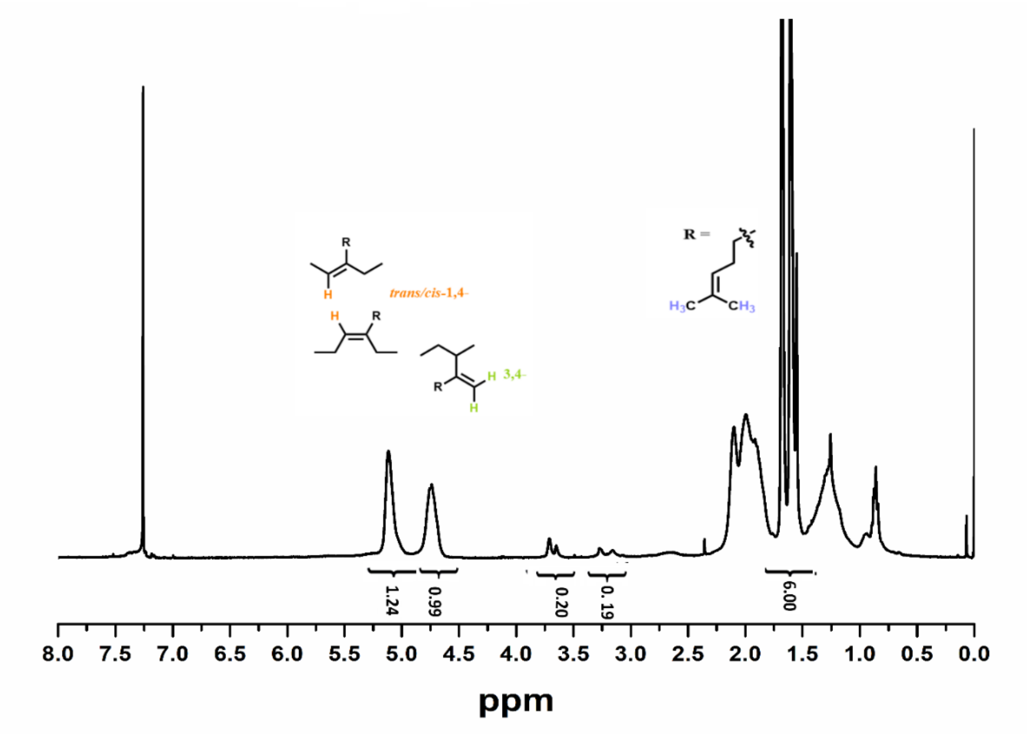

Figure S51. ^{1}H spectrum (500 MHz, $CDCl_3$-*d*) of methyl thioglycolate functionalised PMy. Conditions: [PMy]:[MTG]:[DMPA]: = 1:0.075:0.05, reaction time: 1h. F.D of 1,2-PMy >99%, F.D of 1,4-PMy = 0%, F.D of 3,4-PMy = (1.12-0.07-0.99)/(1.12-0.07) = 6% (**Table S2, entry 1**).

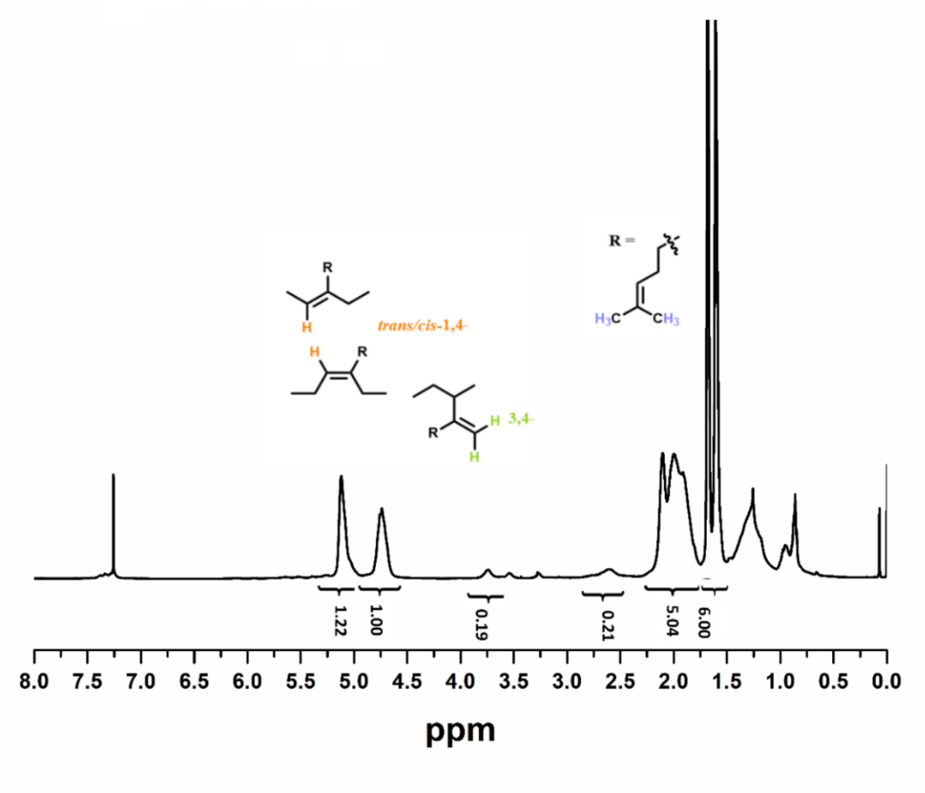

Figure S52. ^{1}H spectrum (500 MHz, $CDCl_3$-*d*) of 1-thioglycerol functionalised PMy. Conditions: [PMy]:[THG]:[DMPA]: = 1:1:0.05, reaction time: 1h. F.D of 1,2-PMy >99%, F.D of 1,4-PMy = 0.8%, F.D of 3,4-PMy = (1.12-0.07-1)/(1.12-0.07) = 5% (**Table S2, entry 19**).

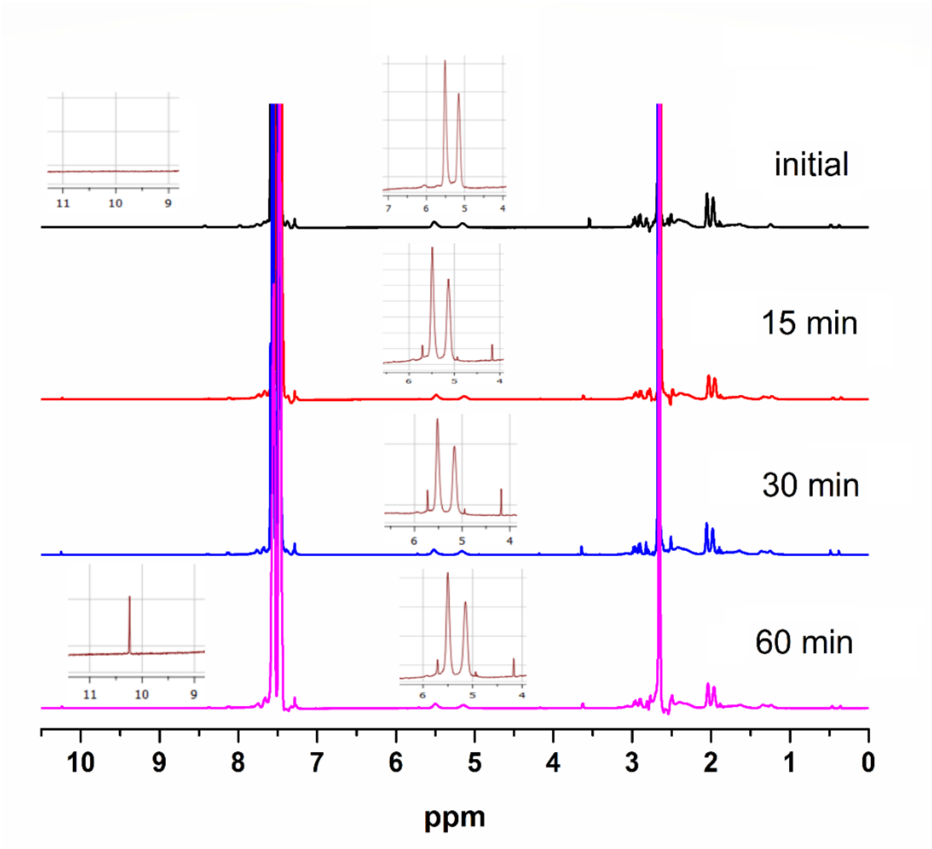

Figure S53. Functionalisation of PMy with 3-mercaptopropionic acid in the presence of DMPA *via* a thiol-ene click reaction ([PMy]:[MAC]:[DMPA] = 1:0.5:0.05); ^{1}H NMR spectra (500 MHz, $CDCl_3$-*d*) of samples obtained at different reaction times 0-1h.

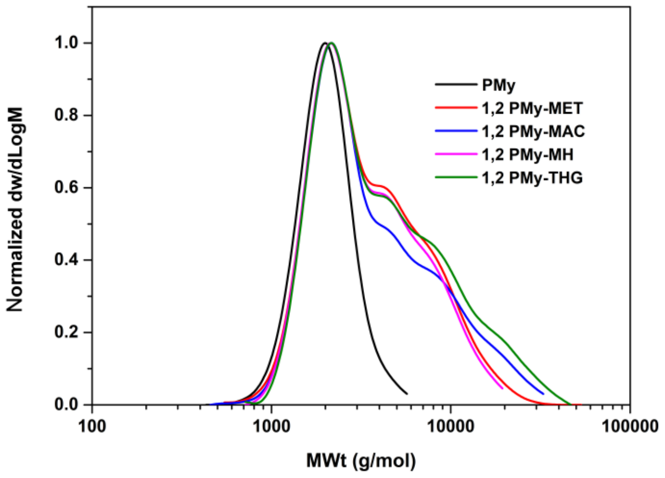

Figure S54. THF-SEC traces of PMy and thiol-ene derivatives after 1h, narrow PMMA standard; Condition: [PMy]:[Thiol]:[DMPA] = 1:0.075:0.05 (**Table S2, entries 1, 2, 6, 11 & 16**).
